# Malononitrile-activated synthesis and anti-cholinesterase activity of styrylquinoxalin-2(1*H*)-ones†

**DOI:** 10.1039/d0ra02816a

**Published:** 2020-04-21

**Authors:** Sheena Mahajan, Nancy Slathia, Vijay K. Nuthakki, Sandip B. Bharate, Kamal K. Kapoor

**Affiliations:** Department of Chemistry, University of Jammu Jammu-180006 India k2kapoor@yahoo.com; Medicinal Chemistry Division, CSIR-Indian Institute of Integrative Medicine Canal Road Jammu-180001 India; Academy of Scientific and Innovative Research (AcSIR) Ghaziabad-201002 India

## Abstract

Herein, we report a base-free malononitrile activated condensation of 3-methylquinoxaline-2(1*H*)-one (3MQ) 1 with aryl aldehydes 3a–3ad for synthesis of styrylquinoxalin-2(1*H*)-ones (SQs) 4a–4ad with excellent yields. In this reaction, malononitrile activates the aldehyde *via* Knoevenagel condensation towards reaction with 3MQ 1 and gets liberated during the course of reaction to yield the desired SQs 4a–4ad. The SQs were evaluated for *in vitro* cholinesterase inhibition and 4n was found to display a mixed type of inhibition of AChE, which was supported by molecular modelling studies. This study has led to the discovery of a new chemotype for cholinesterase inhibition which might be useful in finding a remedy for Alzheimer's disease.

## Abstract 2

SQs displaying anti-Alzheimer activity is serendipitous. Malononitrile as a handle to facilitate nucleophilic attack has been applied for the first time for the easy access of SQs.

## Graphical abstract

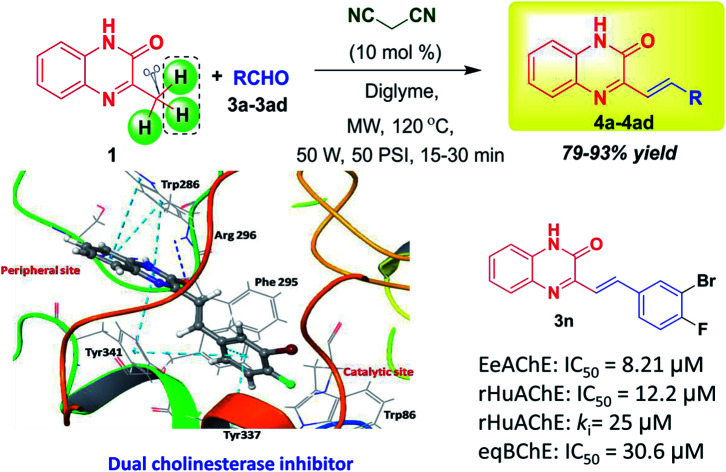

## Introduction

**Fig. 1:**
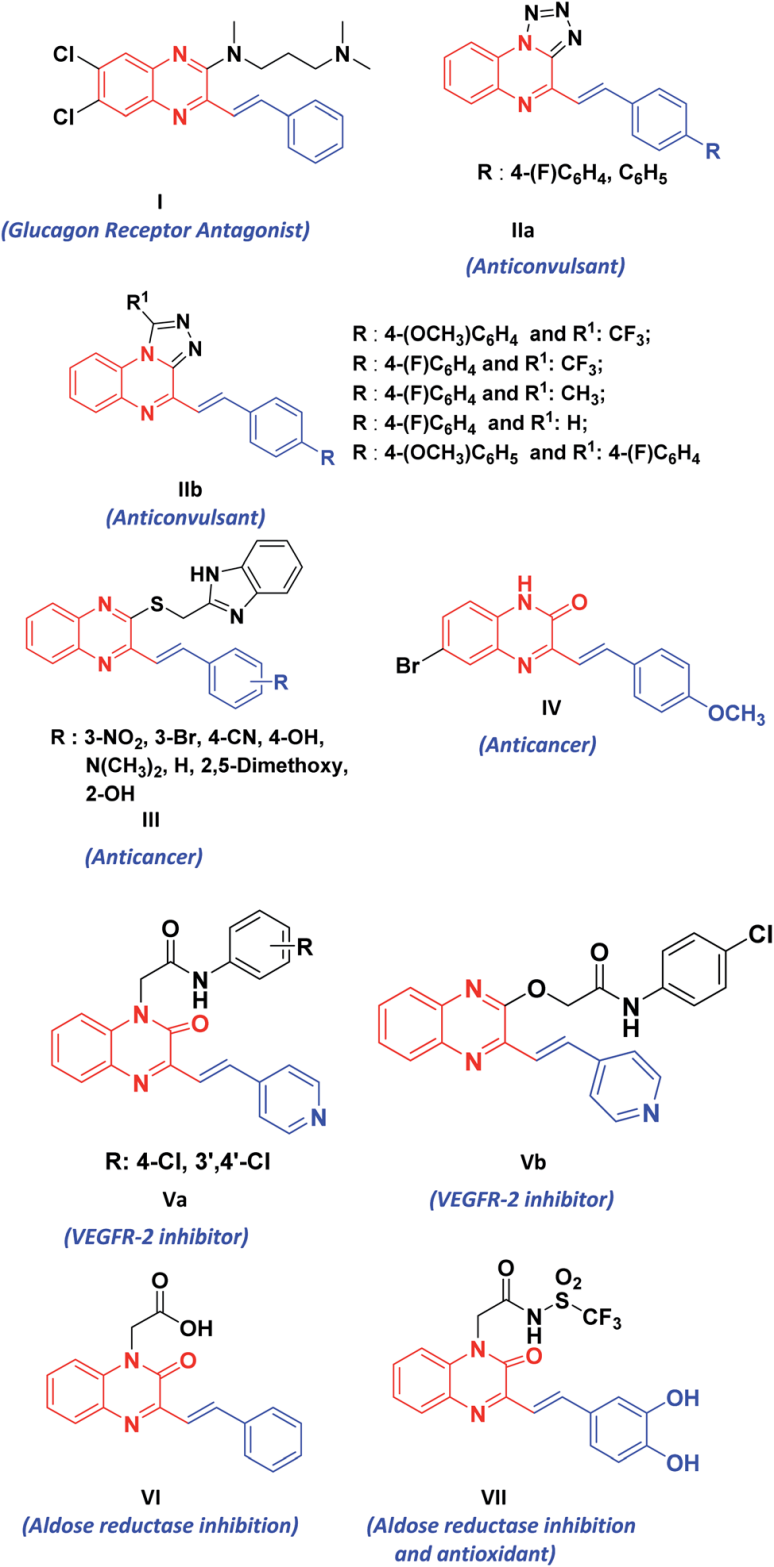
Biologically active styrylquinoxalin-2(1*H*)-ones (SQs).

Styrylquinoxalin-2(1*H*)-ones (SQs) are hybrid molecules of styryl and quinoxalinone. These are promising sources of bioactive scaffolds in the treatment of diverse physiological and pathophysiological events.^1^ Their derivatives serve as useful rigid subunits in the glucagon receptor antagonist^1*b*^ (I), macrocyclic receptors in molecular recognition^1*c*^ (II), anticancer agents^1*d*–*f*^ (III, IV), VEGFR-2 inhibitor^1*g*^ (V), aldose reductase inhibition, antioxidants^1*h*,*i*^ (VI, VII) and are also used as fluorescent probes for amyloid-beta fibrils (Fig. 1).^2^ SQs have an interesting architecture composed of quinoxalinone, a polarized olefenic bond and the presence of H-bond donors and acceptors. In addition to this, they are also known to display numerous photochemical and acid/base responsive properties^3^ due to amide–iminol tautomerism.

**Scheme 1:**
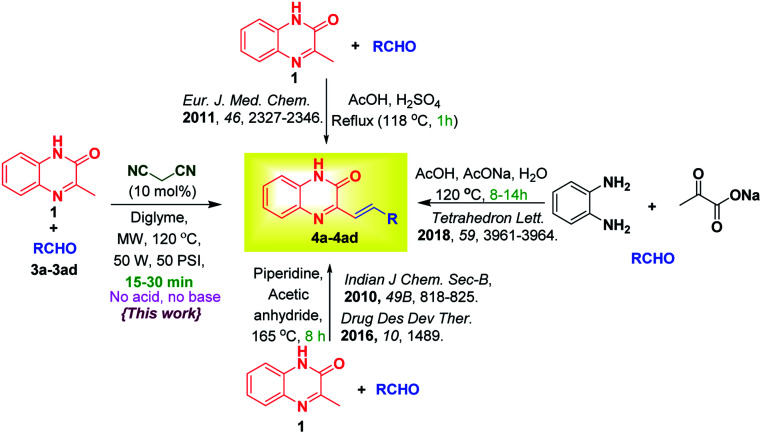
Literature reports and present method on condensation of 3MQ 1 with aldehydes 3a–3ad for synthesis of 3-substituted styrylquinoxalin-2(1*H*)-ones 4a–4ad.

The preparation of SQ mainly involves reaction between 3MQ 1 and aldehyde 3 in presence of piperidine^1*f*,4^ with or without acetic anhydride,^5^ and many a times it requires use of catalytic amount of sulphuric acid.^1*d*^ Menezes *et al.*^6^ reported multicomponent reaction of sodium pyruvate and aldehyde in 20% aqueous acetic acid containing sodium acetate for the synthesis of SQ. Various approaches and the present method are summarized in Scheme 1.

The reported methods for SQs are environmentally aggressive and makes use of hazardous reagents. With these drawbacks in mind, finding a method for easy access of SQs is in great demand. Malononitrile has been employed as an activating handle to carry out various reactions such as [4 + 2] cycloadditions^7^ and for the synthesis of fused cyclic and polycyclic compounds.^8^ So we wished to employ malononitrile handle to activate the aldehyde for synthesis of SQs.

**Fig. 2:**
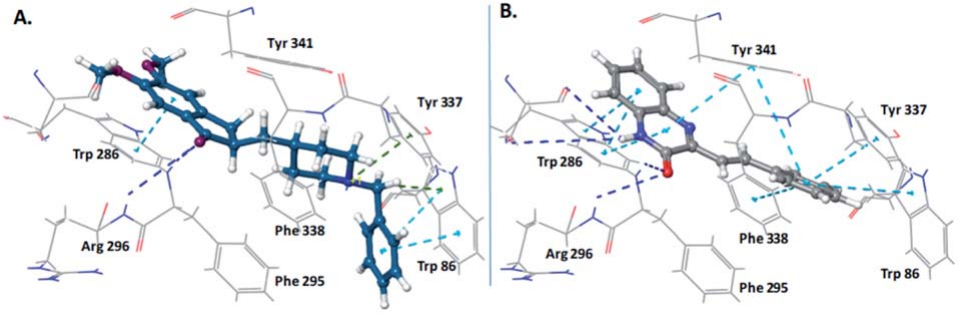
Design of SQs as AChE inhibitors. (A) Interactions of donepezil with AChE; (B) interactions of SQ scaffold with AChE catalytic site gorge.

The restoration of cholinergic neurotransmission is one of the most successful therapy for Alzheimer's disease patients. Various scaffolds^9^ have been reported to inhibit cholinesterase enzymes, but efforts for identification of new scaffolds are continually being made. Our computational studies on the donepezil-bound crystal structure of human acetylcholinesterase (4EY7) has shown that “SQ” scaffold perfectly occupies the active site gorge of acetylcholinesterase; and display key interactions with catalytic as well as peripheral site of the gorge, similar to the clinically used anti-AChE drug donepezil. In addition to this, the styryl linker connecting the quinoxalinone with aryl ring is expected to provide desired orientation of the aryl ring in the catalytic anionic site of the active site gorge of AChE. The interactions of clinically used AChE inhibitor donepezil and newly designed scaffold “SQ” are shown in Fig. 2.

Thus computational design, new synthetic strategy and biological evaluation of the series of SQs is presented in this paper.

## Results and discussion

### Synthesis of 3-substituted styrylquinoxalin-2(1*H*)-ones 4a–4ad

**Scheme 2:**
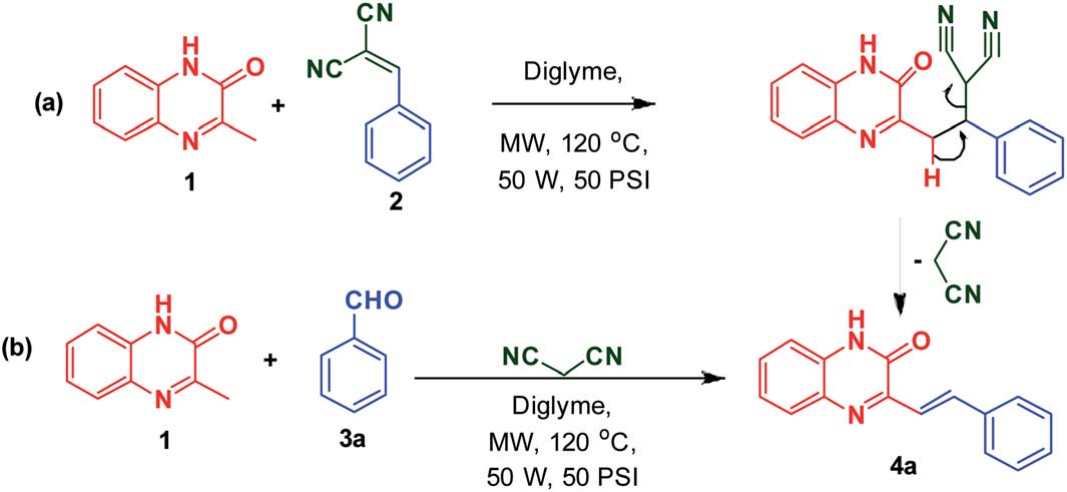
(a) Synthesis of (*E*)-3-(4-methoxystyryl)quinoxalin-2(1*H*)-one 4a from 3MQ 1 and benzylidene malononitrile 2; (b) synthesis of (*E*)-3-(4-methoxystyryl)quinoxalin-2(1*H*)-one 4a from 3MQ 1 and aldehyde 3a.

To begin with, 3MQ 1 (0.5 mmol, 0.08 g) was reacted in glass vial with benzylidene malononitrile 2 (0.5 mmol, 0.09 g) in diglyme (2 mL) under microwave irradiation (at 120 °C, 50 PSI, 50 W) and the progress of the reaction was monitored using thin layer chromatography (TLC). After 15 min, TLC observation reveals the complete disappearance of reactants. Solid obtained was filtered off and washed with ethanol to obtain pure product 4a in 92% yield (Scheme 2a). It indicates the removal of malononitrile during the course of the reaction suggesting its role as an activator.

To investigate the direct use of malononitrile as activator, 3MQ 1 and benzaldehyde 3a (1 : 1 ratio) were reacted in glass vial in presence of malononitrile (100, 50 and 10 mol%) in diglyme under microwave irradiation at 120 °C for 15 min. The formation of desired product 4a was noticed. Absence of malononitrile from the reaction mixture led to no product formation and starting materials were found intact (entry 2, Table 1). Replacing malononitrile with other active methylenes such as ethyl cyanoacetate, methyl acetoacetate, ethyl acetoacetate, 4-hydroxy coumarin did result in product formation. However malononitrile gave the best results (Table 1). Thus, malononitrile was best amongst all active methylenes investigated, yielding desired compound 4a, in 92% yield only in 15 min of reaction time (entry 1, Table 1), suggesting that malononitrile acts as best activator in the reaction.

**Table 1:** Screening of various active methylene compounds for selection of best activator for aldehyde 3a^a^

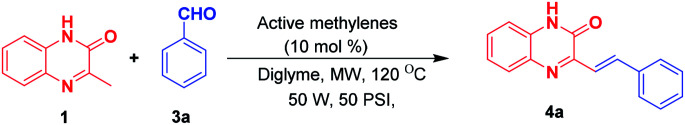			
Entry	Active methylenes	Time (in min)	% yield^b^
1	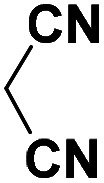	15	92
2	—	30	0
3	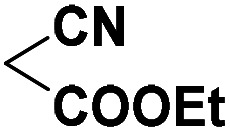	15	83
4	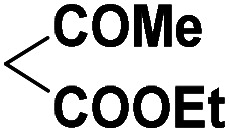	30	87
5	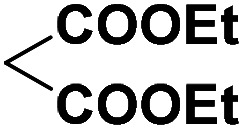	30	90
6	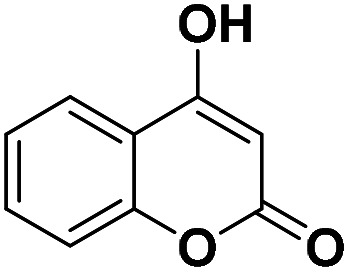	30	82

a Reaction conditions: 3MQ 1 (1.0 mmol), benzaldehyde 3a (1.0 mmol), active methylene (10 mol%), diglyme, MW (at 120 °C, 50 PSI, 50 W).

b Isolated yield.

Replacing diglyme with other solvents (toluene, 1,2-dichloroethane, 1,4-dioxane, acetonitrile, ethanol) led to the observation that the reaction occurs almost with same ease in toluene while in other solvents (1,2-dichloroethane, acetonitrile, ethanol), starting materials are recovered intact with no formation of product. In 1,4-dioxane, low yield of product (65%) was obtained. Thus we decided to use diglyme as a reaction medium since it is less toxic than other solvents such as toluene.^10^

**Scheme 3:**
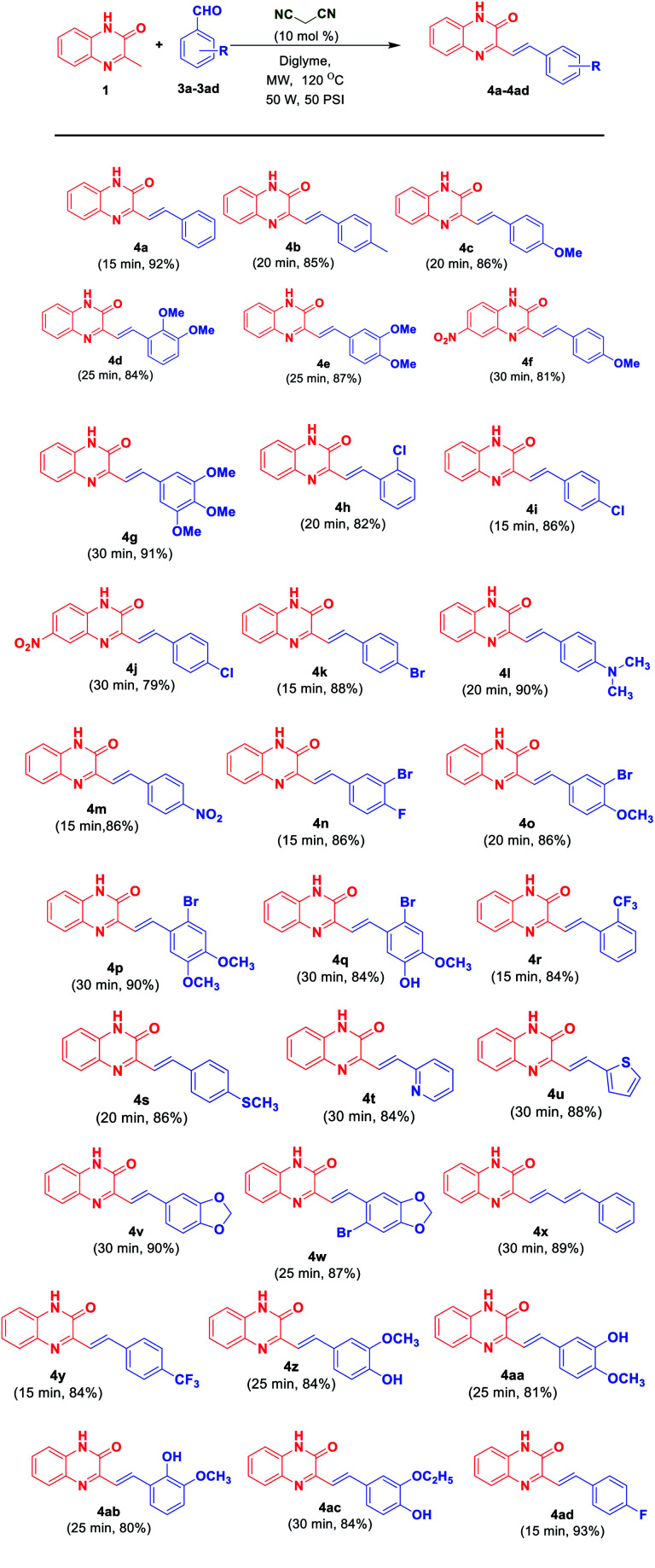
Substrate scope for synthesis of 3-substituted styrylquinoxalin-2(1*H*)-ones 4a–4ad from 3MQ 1 and aryl/hetaryl aldehydes 3a–3ad.

With the optimal reaction conditions in hand, the scope of reaction was then investigated. Diversely substituted aldehydes 3a–3ad generated corresponding SQs 4a–4ad. As evident from Scheme 3, variety of substituents, both electron donating and electron withdrawing on the phenyl moiety of 3 as well as heteroaryl aldehydes (3t, 3u) and cinnamaldehyde (3x) were well tolerated under the reaction conditions, leading to the formation of respective products in good to excellent yields (79–93% yields). It was observed that product formation in lesser time was observed with the aldehydes having electron withdrawing groups at the phenyl ring whereas reaction proceeded with long time for aldehydes having electron donating groups at the phenyl ring and in case having nitro-derivative of 3MQ as starting material. This may be attributed to the fact that the presence of electron withdrawing group at phenyl ring of aromatic aldehydes makes the carbon of double bond (attached directly to the phenyl ring) more electrophilic leading to the facile attack of the nucleophilic carbon of 3MQ. Whereas the presence of electron donating groups at phenyl ring of aromatic aldehydes makes the carbon of double bond (attached directly to the phenyl ring) less electrophilic leading to the attack of nucleophilic carbon of 3MQ in longer time. Also presence of NO_2_ group at the benzene ring of 3MQ decrease the nucleophilicity to small extent of carbon of 3MQ. Thus in that case increase in reaction time is observed to obtained the product.

**Fig. 3:**
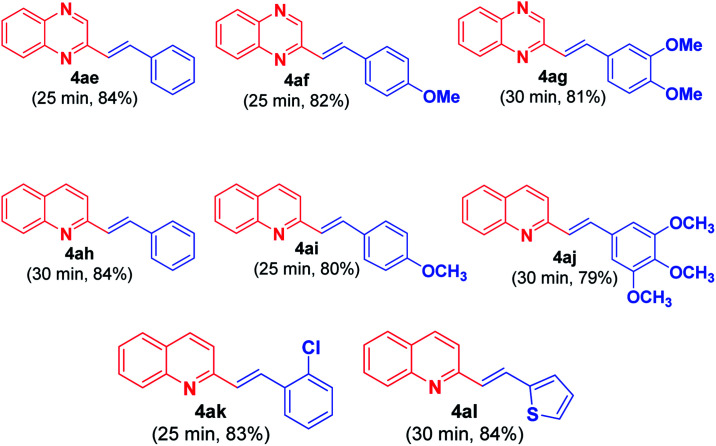
Substrate scope for synthesis of (*E*)-2-substituted styrylquinoxalines 4ae–4ag from 2MQ and aryl aldehydes 3ae–3ag and synthesis of (*E*)-2-substituted styrylquinolines 4ah–4al from 2-methylquinoline and aryl/hetaryl aldehydes 3ah–3al.

Recently, Tang *et al.*^11^ reported acetic acid/1,3-dimethylbarbituric acid-catalyzed direct alkenylation of 2-methylquinolines and 2-methylquinoxalines with aldehydes. The protocol proceeds *via* cascade Knoevenagel condensation followed by Michael addition and retro-Micheal addition to yield the desired alkenyl products. Thus the versatility of this present methodology can be appreciated from the fact that this reaction occurs with less activated methyl group in 2-methylquinoxaline (2MQ) and 2-methylquinoline (Fig. 3) in the absence of acid.

**Scheme 4:**
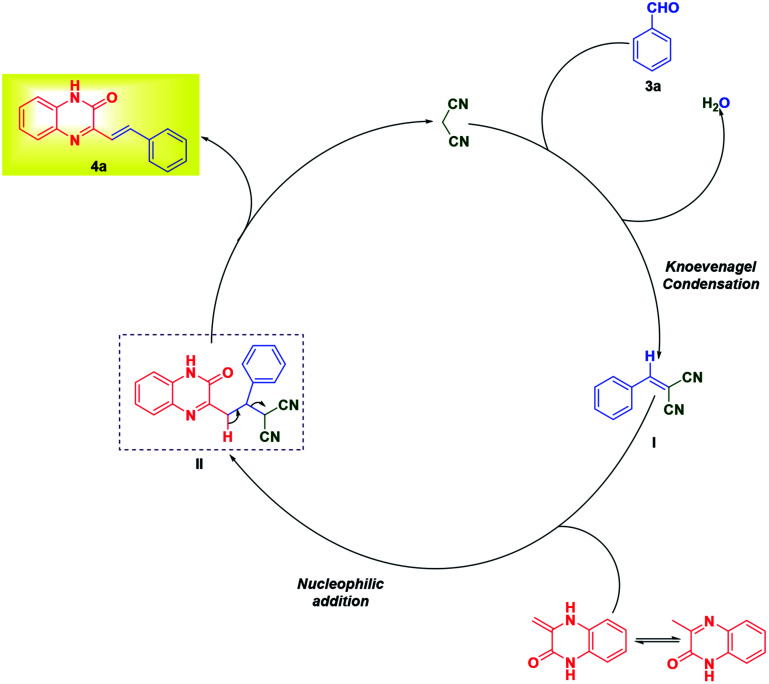
Mechanism for the synthesis of (*E*)-3styrylquinoxalin-2(1*H*)-one 4a.

The formation of product can be rationalized from the mechanism proposed in Scheme 4. Malononitrile, upon Knoevenagel condensation with aldehyde 3a gives intermediate I, which undergoes nucleophilic addition by 3MQ 1 resulting in the formation of II. The intermediate II finally on loss of malononitrile yields SQ 4a.

### *In vitro* cholinesterase inhibition

**Fig. 4:**
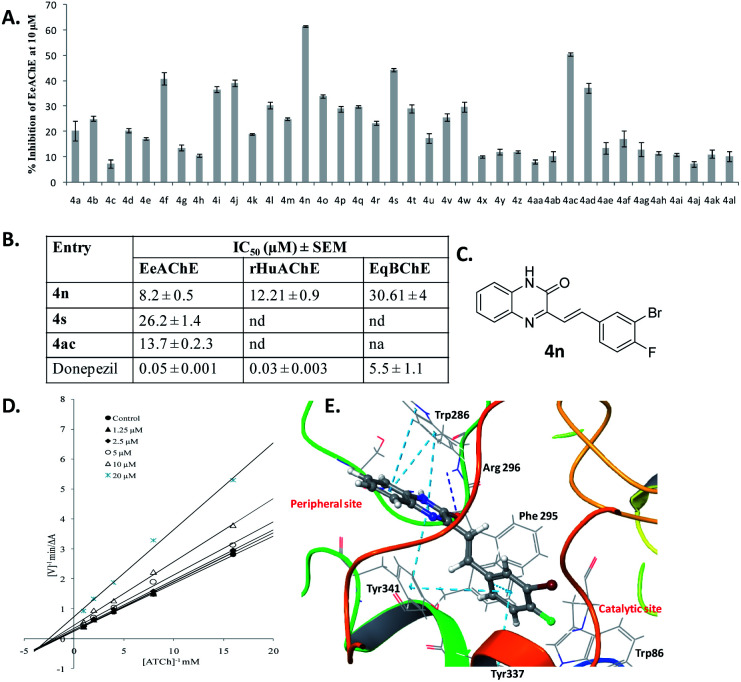
Cholinesterase inhibition activity of styrylquinoxalin-2(1*H*)-ones. (A) Percent inhibition data of all 38 styrylquinoxalin-2(1*H*)-ones against EeAChE at 10 μM. (B) IC_50_ values for inhibition of EeAChE, rHuAChE and EqBChE by 4n and 4s. (C) The chemical structure of styrylquinoxalin-2(1*H*)-one 4n. (D) The Lineweaver–Burk double reciprocal plot representing reciprocal of AChE velocity *versus* reciprocal of different substrate concentrations (0.0625–1 mmol) at five different concentrations of 4n. (E) Interactions of 4n with the active site gorge of human AChE. The light blue dotted lines indicate π–π interactions, dark blue as H-bonding and green-dotted lines as cation–π interactions.

All styrylquinoxalin-2(1*H*)-ones were evaluated for anti-cholinesterase activity against Electrophorus Electricus Acetylcholinesterase (EeAChE) following a Ellman assay.^12^ Donepezil was used as a reference standard in the assay. The percentage inhibition values for all compounds at 10 μM are depicted in Fig. 4A. Further, for IC_50_ determination against EeAChE, compounds 4n, 4s and 4ac were selected based on their inhibitory potential from the preliminary screening data. Compounds 4n, 4s and 4ac inhibited EeAChE with IC_50_ values 8.21, 26.19 and 13.68 μM, respectively. Next, we determined activity of 4n against recombinant human AChE (rHuAChE) and equine butyrylcholinesterase (EqBChE). Compound 4s showed inhibition of rHuAChE and EqBChE with IC_50_ values of 12.21 and 30.61 μM, respectively.

Next, we investigated the mechanism of AChE inhibition, using kinetic studies. AChE active site gorge contains two spatially distinct sites, catalytic anionic site (CAS) and a peripheral anionic site (PAS). The substrate (ACh and ATCh) gets hydrolyzed at CAS whereas, selective inhibitors bind with PAS with high affinity. With a view to sort out the mechanism of AChE inhibition by compound 4n, the kinetic study of enzyme activity was performed using rHuAChE. The Lineweaver–Burk double reciprocal plot shown in Fig. 4D indicate that 4n displays mixed-type of inhibition against rHuAChE. The value of inhibition rate constant (*k*_i_) was determined from the replot of slopes of double reciprocal plot *versus* inhibitor concentrations which was found to be 25.33 μM.

To further validate the obtained enzyme kinetic results and the type of inhibition, we performed docking studies of quinoxaline-2(1*H*)-one 4n with human AChE. The 3D-interaction map of 4n is depicted in Fig. 4E. The interaction pattern clearly indicates that 4n interacts with both the sites (CAS as well as PAS), with more strong interactions at PAS. The styryl ring of quinoxaline-2(1*H*)-one 4n was found oriented towards the bottom of cavity, and it displayed π–π interactions with Tyr 337 residue of anionic subsite and the Tyr 341 residue of PAS. The quinoxaline-2(1*H*)-one nucleus displayed π–π interactions with Trp 286 and Tyr 341 residues of the PAS. The carbonyl oxygen of the quinoxaline-2(1*H*)-one nucleus have shown H-bonding with Phe 295 and Arg 296 of acyl binding pocket. Thus, the observed dual interaction of 4n with CAS as well as PAS accounts for its mixed-type of inhibition.

## Experimental

### Materials and methods

All commercially available reagents were procured from Sigma-Aldrich and were used as received. Microwave-assisted reactions were performed in an oven dried closed glass vial (10 mL) using Discover™ single mode cavity microwave synthesizer (CEM Corporation). The average power and pressure of the radiation in closed glass vial was approximately 50 W and 50 PSI respectively for all reactions performed at 120 °C. The reaction temperature was monitored using external surface sensor (IR sensor). The progress of the reactions was monitored by thin-layer chromatography (TLC) using silica-gel pre-coated aluminium sheets (60 F254, Merck). The visualization of spots was effected by exposure to ultraviolet light (UV) at 365 nm and 254 nm as well as by treatment with iodine vapours. TLC plates were further treated with anisaldehyde reagent followed by heating for the visualization of products. ^1^H NMR, ^13^C NMR and ^19^F NMR spectra in DMSO-d_6_ as the solvent were recorded on Bruker AC-400 spectrometer operating at 400 MHz for ^1^H, 101 MHz for ^13^C and 376 MHz for ^19^F, with tetramethylsilane (TMS) as an internal standard. The chemical shifts (*δ*) for protons are expressed in parts per million (ppm) downfield from TMS. *J* values are expressed in hertz (Hz). The abbreviations s, br s, d, q and m in ^1^H NMR spectra refer to singlet, broad singlet, doublet, quartet and multiplet respectively. Uncorrected melting points (°C) were measured in open glass capillaries using a Perfit melting-point apparatus. The HPLC purity was checked using Shimadzu HPLC system, consisting of Purospher C_18_ (5 μ, 250 × 4.6 mm) column and a PDA detector. LC HRMS was measured using Thermoscientific – exactive consisting of Hypersil C_18_ column with mobile phase as methanol and water (0.1% formic acid). Gradient method used in it was 97% methanol and 3% water for 5 minutes with injected amount of 2 microlitre and 150 μL per minute flow rate of solvent. The source was operated in both positive and negative mode at an ion spray voltage of 3 kV with oven temperature set at 30 °C.

### General procedure for synthesis of 3-substituted styrylquinoxalin-2(1*H*)-ones 4a–4ad

A mixture of 3MQ 1 (1.0 mmol), aryl/heteroaldehyde 3 (1.0 mmol), and malononitrile (10 mol%) was taken in glass vial using diglyme (2 mL) as solvent. The reaction mixture was irradiated under microwave (120 °C, 50 PSI, 50 W) until the completion of the reaction. The obtained solid was filtered and washed with ethanol to get desired products 4a–4ad (79–93% yield).

#### (*E*)-3-Styrylquinoxalin-2(1*H*)-one (4a)^1*f*^

Yellow solid. Yield: 92% (0.23 g). Mp 229–230 °C (228–231 °C).^1*d*^^1^H NMR (400 MHz, DMSO-d_6_, ppm): *δ* 12.43 (br s, 1H), 8.07 (d, 1H, *J* = 16.2 Hz), 7.79 (d, 1H, *J* = 7.8 Hz), 7.73 (d, 1H, *J* = 7.4 Hz), 7.62 (d, 1H, *J* = 16.2 Hz), 7.50–7.41 (m, 5H), 7.34–7.30 (m, 2H). ^13^C{^1^H} NMR (101 MHz, DMSO-d_6_, ppm): *δ* 155.3, 153.4, 137.6, 136.4, 132.8, 132.1, 130.3, 129.9, 129.5, 128.8, 128.1, 124.0, 122.4, 115.7. ESI-MS: *m*/*z* 249 [M + H]^+^.

#### (*E*)-3-(4-Methylstyryl)quinoxalin-2(1*H*)-one (4b)

Yellow solid. Yield: 85% (0.22 g). Mp 235–236 °C (234–236 °C).^1*d*^^1^H NMR (400 MHz, DMSO-d_6_, ppm): *δ* 12.35 (br s, 1H), 7.92 (d, 1H, *J* = 16.2 Hz), 7.67–7.64 (m, 2H), 7.52–7.35 (m, 7H), 3.23 (s, 3H). ^13^C{^1^H} NMR (101 MHz, DMSO-d_6_, ppm): *δ* 155.3, 153.6, 139.7, 137.6, 133.8, 132.9, 132.1, 130.1, 128.8, 128.1, 124.0, 121.4, 115.7, 21.5. ESI-MS: *m*/*z* 263 [M + H]^+^.

#### (*E*)-3-(4-Methoxystyryl)quinoxalin-2(1*H*)-one (4c)^13^

Yellow solid. Yield: 86% (0.24 g). Mp 246–249 °C. ^1^H NMR (400 MHz, DMSO-d_6_, ppm): *δ* 12.44 (br s, 1H), 8.01 (d, 1H, *J* = 16.2 Hz), 7.76–7.66 (m, 3H), 7.50–7.45 (m, 2H), 7.30–7.28 (m, 2H), 6.99 (d, 2H, *J* = 8.5 Hz), 3.79 (s, 3H). ^13^C{^1^H} NMR (101 MHz, DMSO-d_6_, ppm): *δ* 160.8, 155.3, 153.6, 137.4, 132.9, 132.6, 131.9, 129.9, 129.8, 129.1, 128.6, 124.0, 119.8, 115.7, 115.0, 113.7, 55.7. ESI-MS: *m*/*z* 279 [M + H]^+^.

#### (*E*)-3-(2,3-Dimethoxystyryl)quinoxalin-2(1*H*)-one (4d)^1*f*^

Yellow solid. Yield: 84% (0.26 g). Mp 215–217 °C (215.3–217.8 °C). ^1^H NMR (400 MHz, DMSO-d_6_, ppm): *δ* 12.38 (br s, 1H), 8.19 (d, 1H, *J* = 16.4 Hz), 7.69 (d, 2H, *J* = 8.2 Hz), 7.51 (d, 1H, *J* = 16.4 Hz), 7.40–7.19 (m, 3H), 7.04–6.96 (m, 2H), 3.73 (s, 3H), 3.69 (s, 3H). ^13^C{^1^H} NMR (101 MHz, DMSO-d_6_, ppm): *δ* 155.3, 153.7, 153.3, 148.0, 132.8, 132.2, 131.8, 130.3, 130.1, 128.9, 124.9, 124.0, 123.5, 118.8, 115.7, 114.2, 61.3, 56.3. ESI-MS: *m*/*z* 309 [M + H]^+^.

#### (*E*)-3-(3,4-Dimethoxystyryl)quinoxalin-2(1*H*)-one (4e)^13^

Yellow solid. Yield: 87% (0.27 g). Mp 220–225 °C. ^1^H NMR (400 MHz, DMSO-d_6_, ppm): *δ* 12.49 (br s, 1H), 8.04 (d, 1H, *J* = 16.1 Hz), 7.75 (d, 1H, *J* = 7.6 Hz), 7.60–7.46 (m, 2H), 7.39–7.26 (m, 4H), 7.01 (d, 1H, *J* = 8.3 Hz), 3.86 (s, 3H), 3.81 (s, 3H). ^13^C{^1^H} NMR (101 MHz, DMSO-d_6_, ppm): *δ* 155.3, 153.7, 150.7, 149.5, 137.9, 132.9, 131.9, 129.9, 129.3, 128.6, 123.9, 122.3, 120.1, 115.7, 112.2, 110.3, 56.0, 56.0. ESI-MS: *m*/*z* 309 [M + H]^+^.

#### (*E*)-3-(4-Methoxystyryl)-6-nitroquinoxalin-2(1*H*)-one (4f)

Brown solid. Yield: 81% (0.26 g). Mp 262–265 °C. ^1^H NMR (400 MHz, DMSO-d_6_, ppm): *δ* 12.41 (br s, 1H), 8.22 (d, 1H, *J* = 8.9 Hz), 8.06–7.99 (m, 3H), 7.84 (d, 1H, *J* = 8.9 Hz), 7.65 (d, 1H, *J* = 7.6 Hz), 7.45–7.34 (m, 2H), 6.97 (d, 1H, *J* = 8.0 Hz), 3.80 (s, 3H). ^13^C{^1^H} NMR (101 MHz, DMSO-d_6_, ppm): *δ* 161.4, 157.0, 155.3, 155.0, 146.5, 140.0, 139.1, 130.3, 130.1, 129.5, 124.1, 123.8, 118.2, 116.6, 115.0, 111.1, 55.8. ESI-MS: *m*/*z* 324 [M + H]^+^. HPLC purity: 95.1% (*t*_R_ = 33.8 min). HRMS (ESI-TOF) *m*/*z*: [M + H]^+^ calcd for C_17_H_14_N_3_O_4_, 324.0979; found, 324.0984.

#### (*E*)-3-(3,4,5-Trimethoxystyryl)quinoxalin-2(1*H*)-one (4g)

Yellow solid. Yield: 91% (0.31 g). Mp 256–259 °C. ^1^H NMR (400 MHz, DMSO-d_6_, ppm): *δ* 12.49 (br s, 1H), 8.02 (d, 1H, *J* = 16.1 Hz), 7.75 (d, 1H, *J* = 8.1 Hz), 7.58–7.47 (m, 2H), 7.33–7.27 (m, 2H), 7.03 (s, 2H), 3.85 (s, 6H), 3.70 (s, 3H). ^13^C{^1^H} NMR (101 MHz, DMSO-d_6_, ppm): *δ* 155.3, 153.6, 153.4, 139.2, 138.0, 132.8, 132.1, 131.9, 130.2, 129.8, 128.7, 128.3, 124.1, 123.6, 121.7, 115.7, 105.5, 60.6, 56.4. ESI-MS: *m*/*z* 339 [M + H]^+^. HPLC purity: 99.7% (*t*_R_ = 31.0 min). HRMS (ESI-TOF) *m*/*z*: [M + Na]^+^ calcd for C_19_H_18_N_2_NaO_4_, 361.1159; found, 361.1170.

#### (*E*)-3-(2-Chlorostyryl)quinoxalin-2(1*H*)-one (4h)

Yellow solid. Yield: 82% (0.23 g). Mp 242–243 °C (242–244 °C).^1*f*,4*a*^^1^H NMR (400 MHz, DMSO-d_6_, ppm): ^1^H NMR (400 MHz, DMSO-d_6_, ppm): *δ* 8.33 (d, 1H, *J* = 16.2 Hz), 7.88–7.86 (m, 2H), 7.69 (d, 1H, *J* = 8.0 Hz), 7.53 (d, 1H, *J* = 16.2 Hz), 7.45–7.39 (m, 3H), 7.33–7.20 (m, 2H). ^13^C{^1^H} NMR (101 MHz, DMSO-d_6_, ppm): *δ* 155.3, 153.1, 134.3, 133.9, 132.8, 132.7, 132.4, 131.2, 130.7, 130.5, 129.1, 128.3, 127.9, 125.7, 124.0, 115.9. ESI-MS: *m*/*z* 283, 285 [M + H]^+^.

#### (*E*)-3-(4-Chlorostyryl)quinoxalin-2(1*H*)-one (4i)^14^

Yellow solid. Yield: 86% (0.24 g). Mp 259–263 °C. ^1^H NMR (400 MHz, DMSO-d_6_, ppm): *δ* 12.50 (br s, 1H), 8.04 (d, 1H, *J* = 16.2 Hz), 7.79–7.75 (m, 3H), 7.62 (d, 1H, *J* = 16.2 Hz), 7.53–7.48 (m, 3H), 7.34–7.30 (m, 2H). ^13^C{^1^H} NMR (101 MHz, DMSO-d_6_, ppm): *δ* 155.3, 153.3, 136.1, 135.4, 134.2, 132.8, 132.2, 130.5, 129.8, 129.5, 128.9, 124.1, 123.3, 115.8. ESI-MS: *m*/*z* 283, 285 [M + H]^+^.

#### (*E*)-3-(4-Chlorostyryl)-6-nitroquinoxalin-2(1*H*)-one (4j)

Yellow solid. Yield: 79% (0.26 g). Mp 266–268 °C. ^1^H NMR (400 MHz, DMSO-d_6_, ppm): *δ* 12.86 (br s, 1H), 8.49 (s, 1H), 8.32 (d, 1H, *J* = 9.4 Hz), 8.14–8.07 (m, 2H), 7.94 (d, 1H, *J* = 8.7 Hz), 7.80–7.79 (m, 1H), 7.66–7.58 (m, 1H), 7.51–7.41 (m, 2H). ^13^C{^1^H} NMR (101 MHz, DMSO-d_6_, ppm): *δ* 156.8, 154.9, 147.1, 138.7, 137.9, 136.4, 135.0, 135.0, 132.4, 130.3, 130.1, 129.9, 129.6, 122.5, 118.3, 111.2. ESI-MS: *m*/*z* 328, 330 [M + H]^+^. HPLC purity: 99.4% (*t*_R_ = 36.1 min). HRMS (ESI-TOF) *m*/*z*: [M + H]^+^ calcd for C_16_H_11_ClN_3_O_3_, 328.0483; found, 328.0499.

#### (*E*)-3-(4-Bromostyryl)quinoxalin-2(1*H*)-one (4k)

Yellow solid. Yield: 88% (0.29 g). Mp 298–299 °C (298–300 °C).^4*a*^^1^H NMR (400 MHz, DMSO-d_6_, ppm): *δ* 12.52 (br s, 1H), 8.03 (d, 1H, *J* = 16.1 Hz), 7.79 (d, 1H, *J* = 8.1 Hz), 7.72–7.70 (m, 2H), 7.66–7.62 (m, 3H), 7.53–7.50 (m, 1H), 7.34–7.30 (m, 2H). ^13^C{^1^H} NMR (101 MHz, DMSO-d_6_, ppm): *δ* 155.3, 153.2, 136.3, 135.7, 132.8, 132.4, 132.1, 130.6, 130.1, 128.9, 124.2, 123.2, 123.0, 115.8, 79.5. ESI-MS: *m*/*z* 329, 331 [M + H]^+^.

#### (*E*)-3-(4-(Dimethylamino)styryl)quinoxalin-2(1*H*)-one (4l)

Orange solid. Yield: 90% (0.26 g). Mp 242–243 °C (240–244 °C).^1*d*^^1^H NMR (400 MHz, DMSO-d_6_, ppm): *δ* 12.38 (br s, 1H), 7.97 (d, 1H, *J* = 16.0 Hz), 7.72 (d, 1H, *J* = 4.9 Hz), 7.56 (d, 1H, *J* = 8.6 Hz), 7.44–7.37 (m, 3H), 7.28–7.26 (m, 3H), 6.75 (d, 1H, *J* = 8.6 Hz), 2.98 (s, 6H). ^13^C{^1^H} NMR (101 MHz, DMSO-d_6_, ppm): *δ* 155.4, 153.9, 151.6, 138.3, 133.1, 131.7, 129.7, 129.3, 128.3, 124.0, 123.9, 116.5, 115.6, 112.5, 40.3. ESI-MS: *m*/*z* 292 [M + H]^+^.

#### (*E*)-3-(4-Nitrostyryl)quinoxalin-2(1*H*)-one (4m)^6^

Yellow solid. Yield: 86% (0.25 g). Mp 287–289 °C (288–290 °C). ^1^H NMR (400 MHz, DMSO-d_6_, ppm): *δ* 12.17 (br s, 1H), 8.11 (d, 1H, *J* = 8.2 Hz), 8.03–7.99 (m, 3H), 7.88 (d, 1H, *J* = 8.3 Hz), 7.68–7.64 (m, 2H), 7.56 (d, 1H, *J* = 7.7 Hz), 7.47–7.39 (m, 2H). ^13^C{^1^H} NMR (101 MHz, DMSO-d_6_, ppm): *δ* 155.2, 152.9, 147.7, 143.0, 135.0, 132.8, 132.4, 131.0, 129.1, 126.9, 124.5, 124.1, 115.9. ESI-MS: *m*/*z* 294 [M + H]^+^.

#### (*E*)-3-(3-Bromo-4-fluorostyryl)quinoxalin-2(1*H*)-one (4n)

Yellow solid. Yield: 86% (0.30 g). Mp 236–238 °C. ^1^H NMR (400 MHz, DMSO-d_6_, ppm): *δ* 12.52 (br s, 1H), 8.07–7.96 (m, 2H), 7.76–7.74 (m, 2H), 7.57 (d, 1H, *J* = 16.1 Hz), 7.51–7.47 (m, 1H), 7.42–7.38 (m, 1H), 7.30–7.28 (m, 2H). ^13^C{^1^H} NMR (101 MHz, DMSO-d_6_, ppm): *δ* 160.2–157.8 (d, ^1^*J*_CF_ = 242 Hz), 155.2, 153.1, 134.9, 132.9–132.7 (d, ^2^*J*_CF_ = 16.6 Hz), 132.2, 130.5, 129.4–129.3 (d, ^3^*J*_CF_ = 7.6 Hz), 128.9, 124.0, 123.8, 117.8, 117.5, 115.8, 109.3, 109.1. ESI-MS: *m*/*z* 346, 348 [M + H]^+^. HPLC purity: 99.8% (*t*_R_ = 35.4 min). HRMS (ESI-TOF) *m*/*z*: [M + H]^+^ calcd for C_16_H_11_^81^BrFN_2_O, 347.0013; found, 347.0025.

#### (*E*)-3-(3-Bromo-4-methoxystyryl)quinoxalin-2(1*H*)-one (4o)

Yellow solid. Yield: 86% (0.31 g). Mp 260–262 °C. ^1^H NMR (400 MHz, DMSO-d_6_, ppm): *δ* 12.49 (br s, 1H), 8.00–7.96 (m, 2H), 7.77–7.74 (m, 2H), 7.54–7.47 (m, 2H), 7.33–7.29 (m, 2H), 7.18 (d, 1H, *J* = 8.6 Hz), 3.90 (s, 3H). ^13^C{^1^H} NMR (101 MHz, DMSO-d_6_, ppm): *δ* 156.7, 155.3, 153.4, 135.9, 132.8, 132.3, 132.0, 130.7, 130.2, 129.2, 128.7, 124.0, 121.4, 115.7, 113.4, 111.8, 56.9. ESI-MS: *m*/*z* 359, 361 [M + H]^+^. HPLC purity: 92.5% (*t*_R_ = 34.2 min). HRMS (ESI-TOF) *m*/*z*: [M + H]^+^ calcd for C_17_H_14_^79^BrN_2_O_2_, 357.0233; found, 357.0250.

#### (*E*)-3-(2-Bromo-4,5-dimethoxystyryl)quinoxalin-2(1*H*)-one (4p)

Yellow solid. Yield: 90% (0.35 g). Mp 262–264 °C. ^1^H NMR (400 MHz, DMSO-d_6_, ppm): *δ* 12.52 (br s, 1H), 8.36 (d, 1H, *J* = 16.0 Hz), 7.77 (d, 1H, *J* = 7.8 Hz) 7.55–7.48 (m, 2H), 7.42 (s, 1H), 7.33–7.30 (m, 2H), 7.23 (s, 1H), 3.89 (s, 3H), 3.83 (s, 3H). ^13^C{^1^H} NMR (101 MHz, DMSO-d_6_, ppm): *δ* 155.2, 153.4, 151.1, 149.2, 135.7, 132.8, 132.1, 130.3, 128.8, 127.9, 124.0, 123.6, 116.5, 116.1, 115.7, 109.7, 56.5, 56.3. ESI-MS: *m*/*z* 389, 391 [M + H]^+^. HPLC purity: 91.5% (*t*_R_ = 33.3 min). HRMS (ESI-TOF) *m*/*z*: [M + H]^+^ calcd for C_18_H_16_^79^BrN_2_O_3_, 387.0339; found, 387.0366.

#### (*E*)-3-(2-Bromo-5-hydroxy-4-methoxystyryl)quinoxalin-2(1*H*)-one (4q)

Dark green solid. Yield: 84% (0.31 g). Mp 257–259 °C. ^1^H NMR (400 MHz, DMSO-d_6_, ppm): *δ* 12.50 (br s, 1H), 12.33 (s, 1H), 8.23 (d, 1H, *J* = 15.9 Hz), 7.77 (d, 1H, *J* = 7.8 Hz), 7.69 (d, 1H, *J* = 8.0 Hz), 7.51–7.47 (m, 1H), 7.39–7.32 (m, 3H), 7.19 (s, 1H), 3.83 (s, 3H). ^13^C{^1^H} NMR (101 MHz, DMSO-d_6_, ppm): *δ* 155.2, 153.3, 150.5, 147.0, 135.2, 132.8, 132.1, 128.9, 127.9, 124.1, 123.5, 122.5, 116.5, 115.7, 114.7, 113.1, 56.5. ESI-MS: *m*/*z* 375, 377 [M + H]^+^. HPLC purity: 95.4% (*t*_R_ = 31.4 min). HRMS (ESI-TOF) *m*/*z*: [M + H]^+^ calcd for C_17_H_14_^79^BrN_2_O_3_, 373.0182; found, 373.0187.

#### (*E*)-3-(2-(Trifluoromethyl)styryl)quinoxalin-2(1*H*)-one (4r)

Yellow solid. Yield: 84% (0.27 g). Mp 238–240 °C. ^1^H NMR (400 MHz, DMSO-d_6_, ppm): *δ* 12.58 (br s, 1H), 8.45 (d, 1H, *J* = 16.2 Hz), 8.10 (d, 1H, *J* = 7.6 Hz), 7.81–7.70 (m, 3H), 7.68–7.52 (m, 3H), 7.35–7.27 (m, 2H). ^13^C{^1^H} NMR (101 MHz, DMSO-d_6_, ppm): *δ* 155.2, 152.8, 134.9, 133.6, 132.2, 132.2, 131.0, 129.8, 129.1, 128.4 (q, ^1^*J*_CF_ = 275 Hz), 128.1, 127.1, 126.6 (q, ^2^*J*_CF_ = 5 Hz), 115.8. ^19^F NMR (376 MHz, DMSO-d_6_, ppm): *δ* −57.62 (s). ESI-MS: *m*/*z* 317 [M + H]^+^. HPLC purity: 97.4% (*t*_R_ = 34.9 min). HRMS (ESI-TOF) *m*/*z*: [M + H]^+^ calcd for C_17_H_12_F_3_N_2_O, 317.0896; found, 317.0905.

#### (*E*)-3-(4-(Methylthio)styryl)quinoxalin-2(1*H*)-one (4s)

Yellow solid. Yield: 86% (0.25 g). Mp 230–233 °C. ^1^H NMR (400 MHz, DMSO-d_6_, ppm): *δ* 12.51 (br s, 1H), 8.00 (d, 1H, *J* = 16.2 Hz), 7.76 (d, 1H, *J* = 7.3 Hz), 7.65 (d, 1H, *J* = 8.3 Hz), 7.56 (d, 1H, *J* = 16.2 Hz), 7.50–7.47 (m, 1H), 7.33–7.27 (m, 5H), 2.50 (s, 3H). ^13^C{^1^H} NMR (101 MHz, DMSO-d_6_, ppm): *δ* 155.3, 153.4, 140.7, 137.1, 132.8, 132.8, 131.9, 130.2, 129.8, 128.7, 128.6, 128.3, 126.3, 124.1, 123.6, 121.2, 115.7, 14.7. ESI-MS: *m*/*z* 295 [M + H]^+^. HPLC purity: 99.9% (*t*_R_ = 30.6 min). HRMS (ESI-TOF) *m*/*z*: [M + H]^+^ calcd for C_17_H_15_N_2_OS, 295.0900; found, 295.0905.

#### (*E*)-3-(2-(Pyridin-2-yl)vinyl)quinoxalin-2(1*H*)-one (4t)^1*f*^

Brown solid. Yield: 84% (0.21 g). Mp 209–211 °C (208.6–210.5 °C). ^1^H NMR (400 MHz, DMSO-d_6_, ppm): *δ* 12.26 (br s, 1H), 7.69 (d, 1H, *J* = 8.0 Hz), 7.65–7.62 (m, 1H), 7.53 (d, 1H, *J* = 7.9 Hz), 7.48–7.45 (m, 1H), 7.39–7.35 (m, 1H), 7.29–7.25 (m, 3H), 7.19–7.11 (m, 2H). ^13^C{^1^H} NMR (101 MHz, DMSO-d_6_, ppm): *δ* 159.7, 155.4, 155.1, 149.3, 136.6, 132.4, 132.1, 132.0, 131.9, 129.8, 128.3, 123.5, 123.3, 115.7, 115.5. ESI-MS: *m*/*z* 250 [M + H]^+^.

#### (*E*)-3-(2-(Thiophen-2-yl)vinyl)quinoxalin-2(1*H*)-one (4u)^1*f*^

Brown solid. Yield: 88% (0.22 g). Mp 242–244 °C (241.8–243.4 °C). ^1^H NMR (400 MHz, DMSO-d_6_, ppm): *δ* 12.55 (br s, 1H), 8.23 (d, 1H, *J* = 16 Hz), 7.75 (d, 1H, *J* = 8.0 Hz), 7.71–7.66 (m, 1H), 7.51–7.45 (m, 2H), 7.33–7.27 (m, 3H), 7.16 (1H, m). ^13^C{^1^H} NMR (101 MHz, DMSO-d_6_, ppm): *δ* 155.2, 153.1, 148.2, 142.0, 132.9, 132.0, 131.0, 130.7, 130.2, 129.1, 128.7, 124.1, 121.4, 115.8. ESI-MS: *m*/*z* 255 [M + H]^+^.

#### (*E*)-3-(2-(Benzo[*d*][1,3]dioxol-5-yl)vinyl)quinoxalin-2(1*H*)-one (4v)

Dark green solid. Yield: 90% (0.26 g). Mp 340–343 °C. ^1^H NMR (400 MHz, DMSO-d_6_, ppm): *δ* 12.49 (br s, 1H), 7.99 (d, 1H, *J* = 16.1 Hz), 7.75 (d, 1H, *J* = 8.1 Hz), 7.49–7.45 (m, 2H), 7.38 (s, 1H), 7.30–7.29 (m, 2H), 7.21 (d, 1H, *J* = 8.0 Hz), 6.98 (d, 1H, *J* = 7.9 Hz), 6.09 (s, 2H). ^13^C{^1^H} NMR (101 MHz, DMSO-d_6_, ppm): *δ* 155.3, 153.6, 149.0, 148.6, 137.5, 132.9, 132.0, 131.0, 130.0, 128.6, 124.3, 124.0, 120.5, 115.7, 109.1, 106.7, 101.9. ESI-MS: *m*/*z* 293 [M + H]^+^. HPLC purity: 93.8% (*t*_R_ = 31.8 min). HRMS (ESI-TOF) *m*/*z*: [M + H]^+^ calcd for C_17_H_13_N_2_O_3_, 293.0921; found, 293.0928.

#### (*E*)-3-(2-(4-Bromobenzo[*d*][1,3]dioxol-5-yl)vinyl)quinoxalin-2(1*H*)-one (4w)

Brown solid. Yield: 87% (0.32 g). Mp 280–284 °C. ^1^H NMR (400 MHz, DMSO-d_6_, ppm): *δ* 12.48 (br s, 1H), 8.37 (d, 1H, *J* = 16.0 Hz), 7.77 (d, 1H, *J* = 8.0 Hz), 7.69 (d, 1H, *J* = 8.1 Hz), 7.55–7.45 (m, 5H), 6.14 (s, 2H). ^13^C{^1^H} NMR (101 MHz, DMSO-d_6_, ppm): *δ* 155.2, 153.3, 147.9, 147.4, 132.8, 132.3, 131.4, 130.7, 129.1, 128.2, 126.3, 124.1, 118.8, 115.7, 110.4, 103.0. ESI-MS: *m*/*z* 373, 375 [M + H]^+^. HPLC purity: 98.4% (*t*_R_ = 34.9 min). HRMS (ESI-TOF) *m*/*z*: [M + H]^+^ calcd for C_17_H_12_BrN_2_O_3_, 371.0026; found, 371.0037.

#### 3-((1*E*,3*E*)-4-Phenylbuta-1,3-dien-1-yl)quinoxalin-2(1*H*)-one (4x)^1*f*^

Yellow solid. Yield: 89% (0.24 g). Mp 253–256 °C (252.1–254.8 °C). ^1^H NMR (400 MHz, DMSO-d_6_, ppm): *δ* 12.47 (br s, 1H), 7.91–7.85 (m, 1H), 7.77–7.74 (m, 1H), 7.61 (d, 1H, *J* = 7.3 Hz), 7.50–7.46 (m, 2H), 7.41–7.38 (m, 2H), 7.33–7.25 (m, 4H), 7.18 (d, 1H, *J* = 15.4 Hz), 7.05 (d, 1H, *J* = 15.5 Hz). ^13^C{^1^H} NMR (101 MHz, DMSO-d_6_, ppm): *δ* 155.2, 153.5, 138.8, 138.3, 137.0, 133.0, 132.0, 130.2, 129.4, 129.3, 129.0, 128.7, 127.5, 126.5, 124.1, 115.7. ESI-MS: *m*/*z* 275 [M + H]^+^.

#### (*E*)-3-(4-(Trifluoromethyl)styryl)quinoxalin-2(1*H*)-one (4y)^1*f*^

Yellow solid. Yield: 84% (0.27 g). Mp 207–210 °C (206.1–208.7 °C). ^1^H NMR (400 MHz, DMSO-d_6_, ppm): *δ* 12.58 (br s, 1H), 8.12 (d, 1H, *J* = 16.3 Hz), 7.96 (d, 1H, *J* = 8.1 Hz), 7.82–7.72 (m, 5H), 7.56–7.52 (m, 1H), 7.36–7.32 (m, 2H). ^13^C{^1^H} NMR (101 MHz, DMSO-d_6_, ppm): *δ* 155.2, 153.0, 140.4, 135.6, 132.7, 132.2, 130.7, 129.5, 129.3, 129.0, 128.7, 126.2 (q, ^2^*J*_CF_ = 3 Hz), 125.1, 124.6 (q, ^1^*J*_CF_ = 219 Hz), 124.1, 115.8. ^19^F NMR (376 MHz, DMSO-d_6_, ppm): *δ* −61.16 (s). ESI-MS: *m*/*z* 317 [M + H]^+^.

#### (*E*)-3-(4-Hydroxy-3-methoxystyryl)quinoxalin-2(1*H*)-one (4z)

Yellow solid. Yield: 84% (0.25 g). Mp 235–240 °C. ^1^H NMR (400 MHz, DMSO-d_6_, ppm): *δ* 12.46 (br s, 1H), 9.61 (br s, 1H), 7.99 (d, 1H, *J* = 16.4 Hz), 7.74 (d, 1H, *J* = 8.0 Hz), 7.48–7.44 (m, 2H), 7.31–7.27 (m, 3H), 7.16 (d, 1H, *J* = 8.0 Hz), 6.83 (d, 1H, *J* = 7.6 Hz), 3.86 (s, 3H). ^13^C{^1^H} NMR (101 MHz, DMSO-d_6_, ppm): *δ* 155.3, 153.8, 149.0, 148.5, 138.2, 132.9, 131.9, 129.8, 128.5, 128.1, 124.0, 122.6, 119.1, 116.2, 115.6, 111.0, 56.0. MS: *m*/*z* 295 [M + H]^+^. HPLC purity: 99.0% (*t*_R_ = 24.8 min). HRMS (ESI-TOF) *m*/*z*: [M + H]^+^ calcd for C_17_H_14_N_2_O_3_, 295.1077; found, 295.1083.

#### (*E*)-3-(3-Hydroxy-4-methoxystyryl)quinoxalin-2(1*H*)-one (4aa)

Yellow solid. Yield: 81% (0.24 g). Mp 243–246 °C (243–245 °C).^6^^1^H NMR (400 MHz, DMSO-d_6_, ppm): *δ* 12.47 (br s, 1H), 9.28 (br s, 1H), 7.92 (d, 1H, *J* = 16.0 Hz), 7.76 (d, 1H, *J* = 8.0 Hz), 7.49–7.39 (m, 2H), 7.32–7.28 (m, 2H), 7.16–7.13 (m, 2H), 6.98 (d, 1H, *J* = 8.0 Hz), 3.82 (s, 3H). ^13^C{^1^H} NMR (101 MHz, DMSO-d_6_, ppm): *δ* 155.3, 153.6, 149.8, 147.3, 137.6, 132.9, 132.0, 129.9, 129.4, 128.6, 123.9, 121.1, 119.9, 115.7, 113.7, 112.6, 56.0. ESI-MS: *m*/*z* 295 [M + H]^+^.

#### (*E*)-3-(2-Hydroxy-3-methoxystyryl)quinoxalin-2(1*H*)-one (4ab)

Yellow solid. Yield: 80% (0.24 g). Mp 210–220 °C. ^1^H NMR (400 MHz, DMSO-d_6_, ppm): *δ* 12.47 (br s, 1H), 9.37 (br s, 1H), 8.35 (d, 1H, *J* = 16.4 Hz), 7.79 (d, 1H, *J* = 8.0 Hz), 7.64 (d, 1H, *J* = 16.4 Hz), 7.48 (t, 1H, *J* = 7.2 Hz), 7.32–7.25 (m, 3H), 6.97 (d, 1H, *J* = 8.0 Hz), 6.83 (t, 1H, *J* = 8.0 Hz), 3.84 (s, 3H). ^13^C{^1^H} NMR (101 MHz, DMSO-d_6_, ppm): *δ* 155.3, 153.9, 148.4, 146.1, 132.9, 132.9, 131.9, 130.0, 128.7, 124.0, 123.4, 121.5, 119.7, 119.4, 115.7, 112.8, 56.3. ESI-MS: *m*/*z* 295 [M + H]^+^. HPLC purity: 99.2% (*t*_R_ = 26.3 min). HRMS (ESI-TOF) *m*/*z*: [M + H]^+^ calcd for C_17_H_14_N_2_O_3_, 295.1077; found, 295.1088.

#### (*E*)-3-(3-Ethoxy-4-hydroxystyryl)quinoxalin-2(1*H*)-one (4ac)

Yellow solid. Yield: 84% (0.26 g). Mp 175–180 °C. ^1^H NMR (400 MHz, DMSO-d_6_, ppm): *δ* 12.44 (br s, 1H), 9.50 (br s, 1H), 7.98 (d, 1H, *J* = 16 Hz), 7.74 (d, 1H, *J* = 7.6 Hz), 7.48–7.43 (m, 2H), 7.31–7.28 (m, 3H), 7.15 (d, 1H, *J* = 8.0 Hz), 6.85 (d, 1H, *J* = 8.0 Hz), 4.12 (q, 2H, *J* = 6.8 Hz, 6.8 Hz), 1.37 (t, 3H, *J* = 6.8 Hz). ^13^C{^1^H} NMR (101 MHz, DMSO-d_6_, ppm): *δ* 155.3, 153.8, 149.2, 147.6, 138.2, 132.9, 131.9, 129.8, 128.5, 128.0, 123.9, 122.5, 119.0, 116.3, 115.6, 112.3, 64.3, 15.2 MS: *m*/*z* 309 [M + H]^+^. HPLC purity: 95.9% (*t*_R_ = 26.4 min). HRMS (ESI-TOF) *m*/*z*: [M + H]^+^ calcd for C_18_H_16_N_2_O_3_, 309.1234; found, 309.1242.

#### (*E*)-3-(4-Fluorostyryl)quinoxalin-2(1*H*)-one (4ad)

Yellow solid. Yield: 93% (0.25 g). Mp 240–242 °C (239.1–241.4 °C).^1*f*^^1^H NMR (400 MHz, DMSO-d_6_, ppm): *δ* 12.47 (br s, 1H), 8.05 (d, 1H, *J* = 16.4 Hz), 7.82–7.76 (m, 3H), 7.57 (d, 1H, *J* = 16.4 Hz), 7.50 (t, 1H, *J* = 7.6 Hz), 7.33–7.24 (m, 4H). ^13^C{^1^H} NMR (101 MHz, DMSO-d_6_, ppm): *δ* 164.4, 161.9, 155.2, 153.4, 136.3, 133.1, 133.1, 132.8, 132.1, 130.4, 130.3, 130.3, 128.8, 124.0, 122.3, 116.5, 116.3, 115.7. MS: *m*/*z* 267 [M + H]^+^.

### General procedure for synthesis of 2-substituted styrylquinoxalines 4ae–4ag

A mixture of 2MQ (1.0 mmol), arylaldehyde 3 (1.0 mmol), and malononitrile (10 mol%) was taken in glass vial using diglyme (2 mL) as solvent. The reaction mixture was irradiated under microwave (120 °C, 50 PSI, 50 W) until the completion of the reaction. The obtained solid was filtered and washed with ethanol to get desired products 4ae–4ag (81–84% yield).

#### (*E*)-2-Styrylquinoxaline (4ae)

Brown solid. Yield: 84% (0.19 g). Mp 104–106 °C (103–105 °C).^15^^1^H NMR (400 MHz, CDCl_3_, ppm): *δ* 9.08 (s, 1H), 8.10 (d, 1H, *J* = 16.4 Hz), 7.90 (d, 1H, *J* = 16.4 Hz), 7.81–7.69 (m, 5H), 7.48–7.38 (m, 4H). ^13^C{^1^H} NMR (101 MHz, CDCl_3_, ppm): *δ* 150.7, 144.5, 142.5, 141.6, 136.5, 136.0, 130.4, 129.3, 129.3, 129.2, 129.0, 127.5, 125.3. MS: *m*/*z* 233 [M + H]^+^.

#### (*E*)-2-(4-Methoxystyryl)quinoxaline (4af)

Yellow solid. Yield: 82% (0.21 g). Mp 122–123 °C (121–122 °C).^16^^1^H NMR (400 MHz, CDCl_3_, ppm): *δ* 9.05 (s, 1H), 8.08 (t, 2H, *J* = 6.8 Hz), 7.86 (d, 1H, *J* = 16.0 Hz), 7.80–7.70 (m, 3H), 7.64 (d, 1H, *J* = 8.8 Hz), 7.30–7.26 (m, 2H), 6.98 (d, 1H, *J* = 8.4 Hz), 3.89 (s, 3H). ^13^C{^1^H} NMR (101 MHz, CDCl_3_, ppm): *δ* 160.6, 151.0, 144.5, 142.5, 141.4, 136.1, 130.3, 129.1, 129.0, 129.0, 128.8, 123.1, 114.4, 55.4. MS: *m*/*z* 263 [M + H]^+^.

#### (*E*)-2-(3,4-Dimethoxystyryl)quinoxaline (4ag)

Yellow solid. Yield: 81% (0.24 g). Mp 155–157 °C (154–156 °C).^17^^1^H NMR (400 MHz, CDCl_3_, ppm): *δ* 9.07 (s, 1H), 8.07 (t, 2H, *J* = 8.8 Hz), 7.85–7.70 (m, 3H), 7.30–7.22 (m, 3H), 6.93 (d, 1H, *J* = 8.0 Hz), 3.99 (s, 3H), 3.96 (s, 3H). ^13^C{^1^H} NMR (101 MHz, CDCl_3_, ppm): *δ* 150.9, 150.3, 149.3, 144.3, 142.5, 141.4, 136.3, 130.4, 129.1, 129.0, 123.4, 121.7, 111.1, 109.0, 56.0, 55.9. MS: *m*/*z* 293 [M + H]^+^.

### General procedure for synthesis of 2-substituted styrylquinolines 4ah–4al

A mixture of 2-methylquinoline (1.0 mmol), aryl/heteroaldehyde 3 (1.0 mmol), and malononitrile (10 mol%) was taken in glass vial using diglyme (2 mL) as solvent. The reaction mixture was irradiated under microwave (120 °C, 50 PSI, 50 W) until the completion of the reaction. The obtained solid was filtered and washed with ethanol to get desired products 4ah–4al (79–84% yield).

#### (*E*)-2-Styrylquinoline (4ah)

White solid. Yield: 84% (0.19 g). Mp 98–100 °C (99–101 °C).^18^ (400 MHz, DMSO-d_6_, ppm): *δ* 8.36 (d, 1H, *J* = 8.4 Hz), 8.01 (d, 1H, *J* = 8.4 Hz), 7.95 (d, 1H, *J* = 8.4 Hz), 7.89 (d, 1H, *J* = 9.2 Hz), 7.84–7.75 (m, 4H), 7.58–7.53 (m, 2H), 7.49–7.43 (m, 2H), 7.38–7.34 (m, 1H). ^13^C{^1^H} NMR (101 MHz, DMSO-d_6_, ppm): *δ* 156.1, 148.1, 137.0, 136.7, 134.6, 130.3, 129.4, 129.2, 129.1, 128.3, 127.7, 127.5, 126.7, 120.4. MS: *m*/*z* 232 [M + H]^+^.

#### (*E*)-2-(4-Methoxystyryl)quinoline (4ai)

White solid. Yield: 80% (0.21 g). Mp 127–129 °C (126–128 °C).^15^ (400 MHz, DMSO-d_6_, ppm): *δ* 8.33 (d, 1H, *J* = 8.4 Hz), 7.99–7.92 (m, 2H), 7.85–7.82 (m, 2H), 7.78–7.69 (m, 4H), 7.54 (t, 1H, *J* = 7.2 Hz), 7.35 (d, 1H, *J* = 16.4 Hz), 7.01 (d, 1H, *J* = 8.0 Hz), 3.81 (s, 3H). ^13^C{^1^H} NMR (101 MHz, DMSO-d_6_, ppm): *δ* 160.3, 156.4, 148.1, 136.8, 134.3, 130.2, 129.3, 129.2, 129.0, 128.3, 127.3, 126.9, 126.4, 120.2, 114.8, 55.7. MS: *m*/*z* 262 [M + H]^+^.

#### (*E*)-2-(3,4,5-Trimethoxystyryl)quinoline (4aj)

Yellow solid. Yield: 79% (0.25 g). Mp 139–141 °C (138–140 °C).^18^ (400 MHz, DMSO-d_6_, ppm): *δ* 8.37–8.35 (m, 1H), 7.96–7.76 (m, 5H), 7.56–7.48 (m, 2H), 7.10 (s, 2H), 3.88 (s, 3H), 3.71 (s, 3H), 3.37 (s, 3H). ^13^C{^1^H} NMR (101 MHz, DMSO-d_6_, ppm): *δ* 156.2, 153.6, 148.1, 138.5, 137.0, 134.8, 132.4, 130.3, 129.0, 128.6, 128.3, 127.4, 126.6, 120.3, 105.1, 60.6, 56.4. MS: *m*/*z* 322 [M + H]^+^.

#### (*E*)-2-(2-Chlorostyryl)quinoline (4ak)

White solid. Yield: 83% (0.22 g). Mp 80–82 °C (79–81 °C).^18^ (400 MHz, DMSO-d_6_, ppm): *δ* 8.39 (d, 1H, *J* = 8.8 Hz), 8.13 (d, 1H, *J* = 16 Hz), 8.04–8.02 (m, 2H), 7.97 (d, 1H, *J* = 8.0 Hz), 7.85 (d, 1H, *J* = 8.4 Hz), 7.77 (t, 1H, *J* = 7.2 Hz), 7.61–7.55 (m, 3H), 7.45–7.37 (m, 2H). ^13^C{^1^H} NMR (101 MHz, DMSO-d_6_, ppm): *δ* 155.4, 148.1, 137.3, 134.4, 133.4, 132.1, 130.6, 130.5, 130.4, 129.4, 129.3, 128.3, 128.2, 127.9, 127.7, 127.0, 121.1. MS: *m*/*z* 266, 268 [M + H]^+^.

#### (*E*)-2-(2-(Thiophen-2-yl)vinyl)quinoline (4al)

Brown solid. Yield: 84% (0.20 g). Mp 101–103 °C (100–102 °C).^18^ (400 MHz, DMSO-d_6_, ppm): *δ* 8.34 (d, 1H, *J* = 8.4 Hz), 8.04 (d, 1H, *J* = 16 Hz), 7.98–7.93 (m, 2H), 7.84 (d, 1H, *J* = 8.8 Hz), 7.75 (t, 1H, *J* = 7.2 Hz), 7.62 (d, 1H, *J* = 5.2 Hz), 7.56 (t, 1H, *J* = 7.6 Hz), 7.45–7.44 (m, 1H), 7.20–7.14 (m, 2H). ^13^C{^1^H} NMR (101 MHz, DMSO-d_6_, ppm): *δ* 155.6, 148.1, 141.9, 137.0, 130.4, 129.4, 129.0, 128.8, 128.3, 128.1, 127.7, 127.6, 127.5, 126.6, 120.5. MS: *m*/*z* 238 [M + H]^+^.

### *In vitro* AChE and BChE inhibition assay

The inhibitory potential of all synthesized compounds on EeAChE was determined using modified Ellman assay,^19^ as described in our publications.^20^ The activity of best compound 4n was also determined on recombinant human AChE. The kinetic study of interaction of 4n [using five different concentrations of the substrate (0.0625 mmol to 1 mmol) for each concentration of 4n] with rHuAChE was performed using similar assay protocol as described earlier. Each experiment was performed in triplicate. Lineweaver–Burk double reciprocal plot was plotted from [*V*] and [*S*] values. Slopes of these reciprocal plots were then plotted against the concentration of the inhibitor and *k*_i_ was determined as the ratio of the replot intercept to the replot slope.

### Molecular modelling

The crystal structure of human AChE (PDB ID 4EY7),^21^ human BChE (PDB ID 6EP4)^12^ were retrieved from protein data bank and were used for molecular modelling studies under default settings from Glide. The docking was performed as described earlier.^20^

## Conclusions

In conclusion, herein we have devised a new base-free simple and efficient malononitrile-activated condensation of 3MQ with aryl aldehydes for synthesis of SQs in excellent yields. Using this protocol, 38 SQs were prepared. Reaction displayed excellent substrate scope and prepared compounds were found to possess weak to moderate activity against cholinesterase enzymes, opening up a new chemotype for dual inhibition of these enzymes. Further exploration of this scaffold is warranted to discover new anti-Alzheimer lead candidate.

## Conflicts of interest

There are no conflicts to declare.

## Supplementary Material

RA-010-D0RA02816A-s001
