# Supplementary material for: Malononitrile-activated synthesis and anti-cholinesterase activity of styrylquinoxalin-2(1H)-ones
Source: RSC Adv. 2020 Apr 21;10(27):15966–75. doi: 10.1039/d0ra02816a (PMC9052867; doi:10.1039/d0ra02816a)

Electronic Supplementary Material (ESI) for RSC Advances.
This journal is © The Royal Society of Chemistry 2020

Supporting Information

Malononitrile-activated Synthesis and anti-cholinesterase activity of Styrylquinoxalin-2(1*H*)-ones

Sheena Mahajan, [†a] Nancy Slathia, [†a] Vijay K. Nuthakki,[b] Sandip B. Bharate[b] and Kamal K. Kapoor[*a]

[a] Department of Chemistry
University of Jammu
Jammu- 180 006, India
E-mail: k2kapoor@yahoo.com

[b] Medicinal Chemistry Division
CSIR-Indian Institute of Integrative Medicine
Canal Road, Jammu-180001, India;
Academy of Scientific and Innovative Research (AcSIR),
Ghaziabad-201002, India

* Corresponding Author

† Sheena Mahajan and Nancy Slathia have equally contributed as a first author.

4a. ***(E)-3-styrylquinoxalin-2(1H)-one***

^{1}H NMR spectrum (400 MHz, DMSO-d_6)

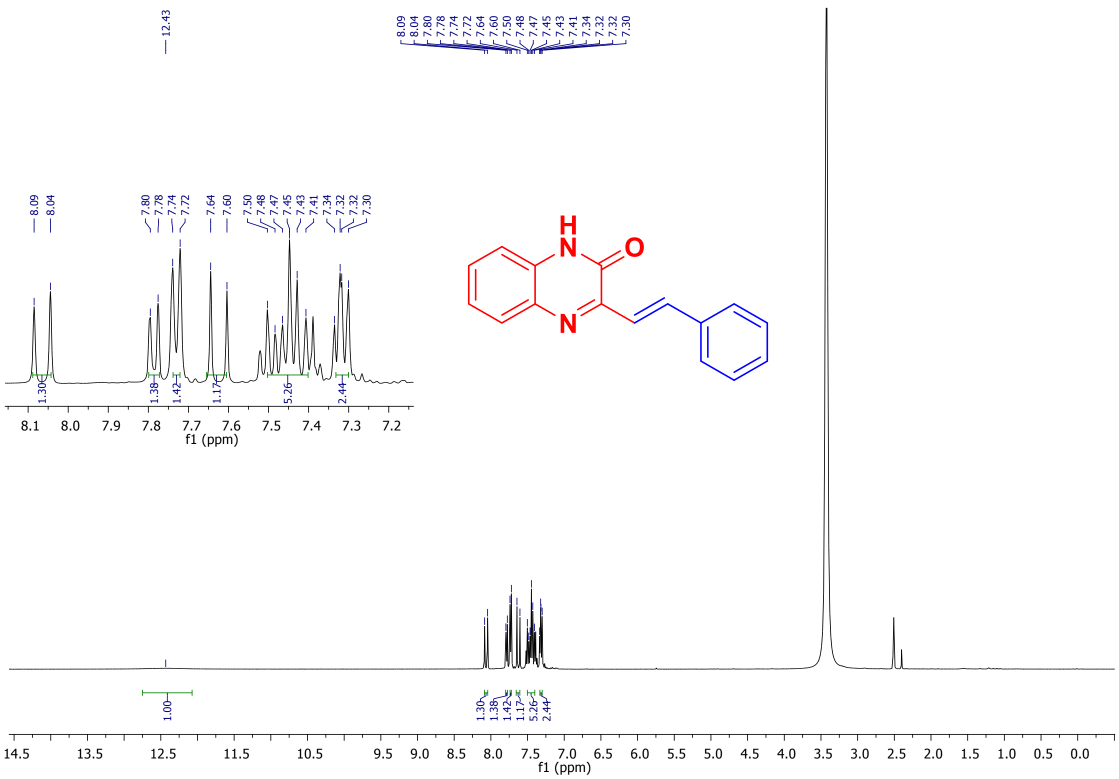

^{13}C NMR spectrum (101 MHz, DMSO-d_6)

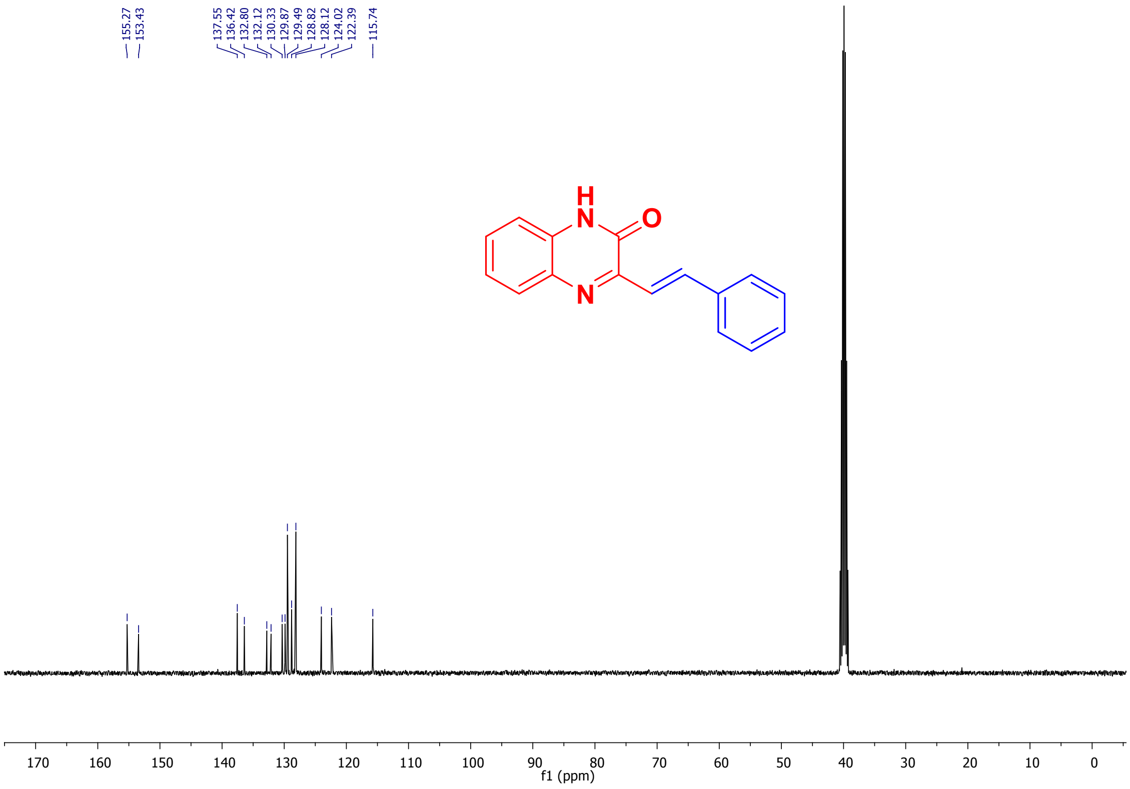

4b. ***(E)-3-(4-methylstyryl)quinoxalin-2(1H)-one***

^{1}H NMR spectrum (400 MHz, DMSO-d$_6$)

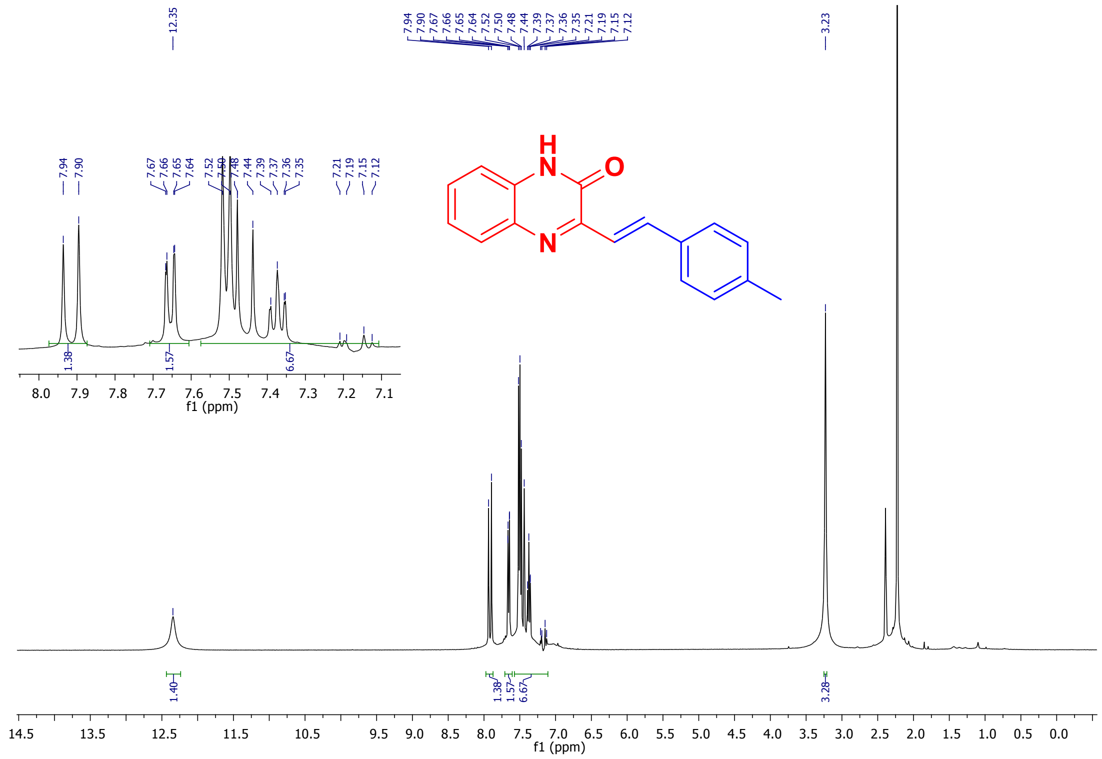

^{13}C NMR spectrum (101 MHz, DMSO-d$_6$)

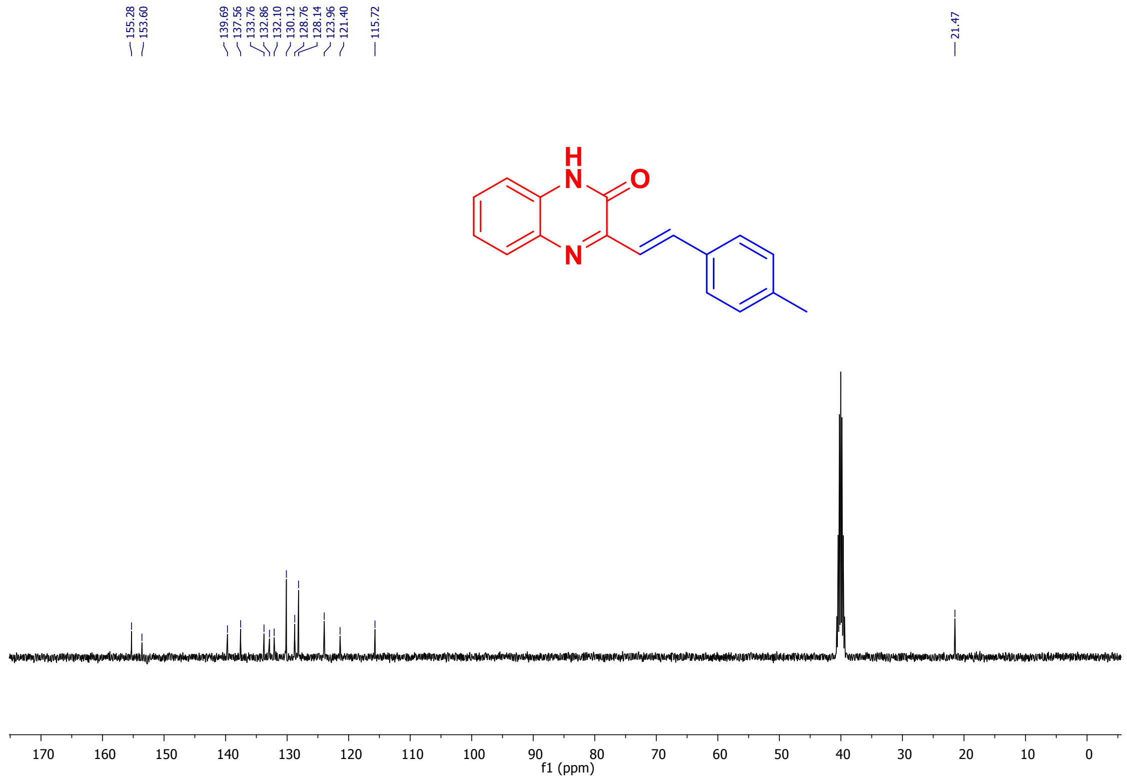

4c. ***(E)-3-(4-methoxystyryl)quinoxalin-2(1H)-one***

^{1}H NMR spectrum (400 MHz, DMSO-d_6)

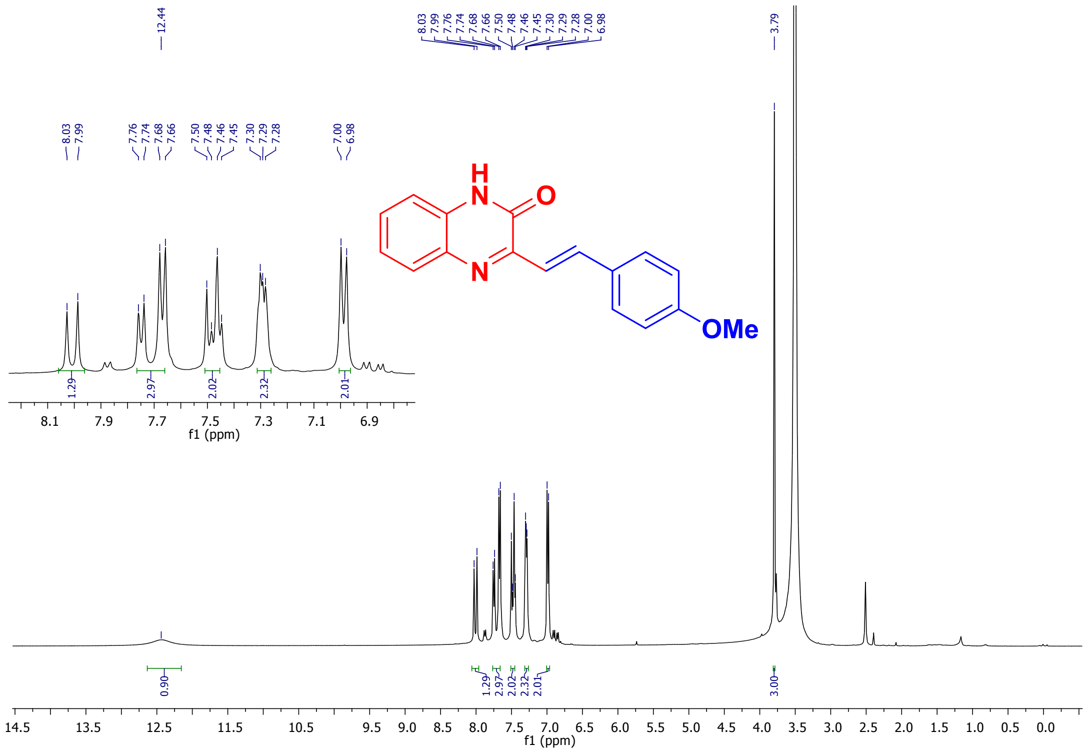

^{13}C NMR spectrum (101 MHz, DMSO-d_6)

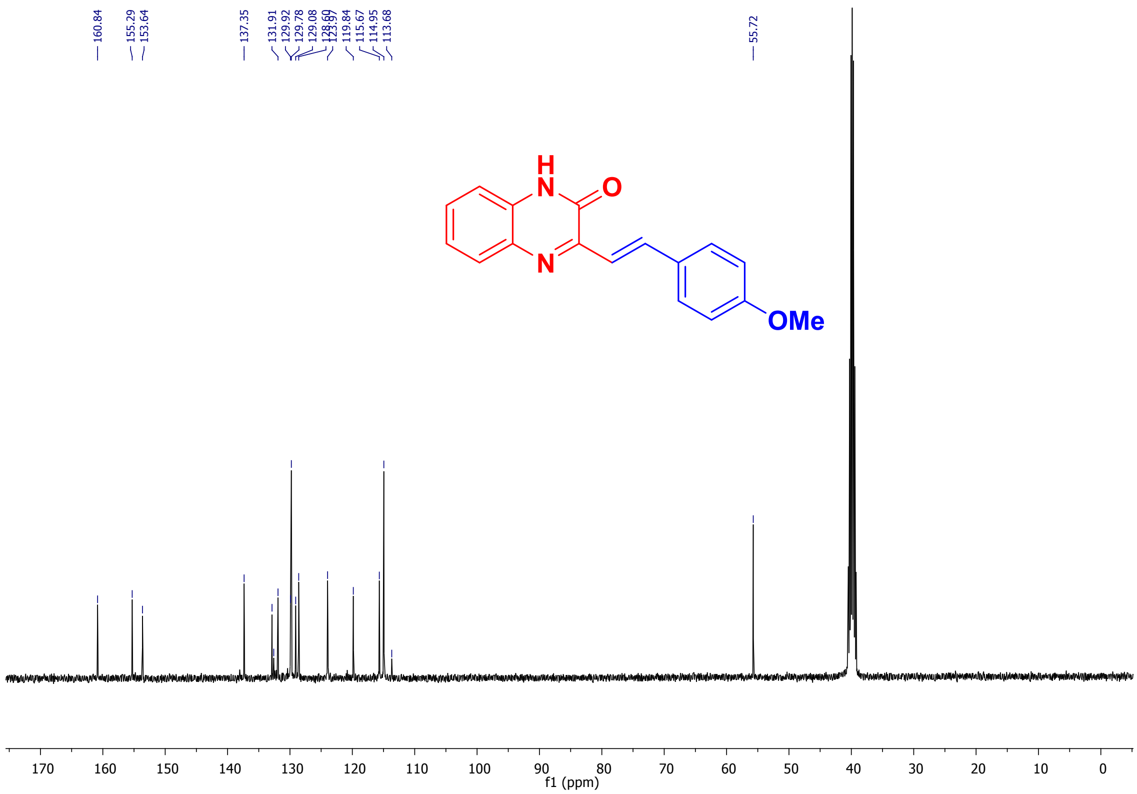

4d. ***(E)-3-(2,3-dimethoxystyryl)quinoxalin-2(1H)-one***

^{1}H NMR spectrum (400 MHz, DMSO-d$_6$)

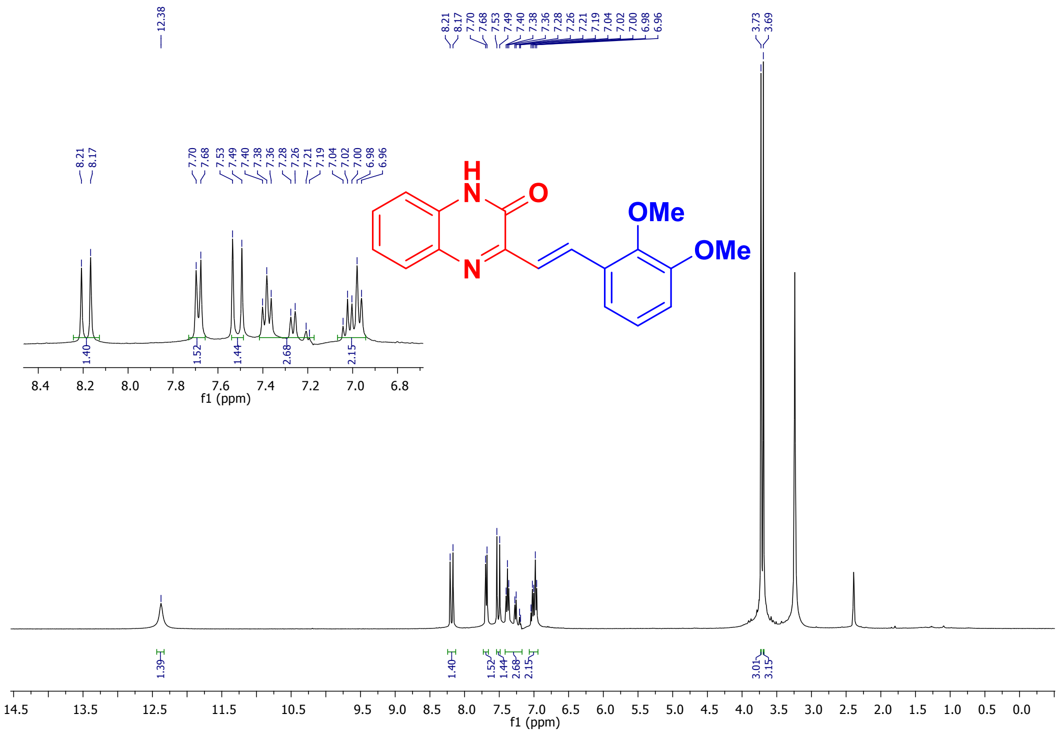

^{13}C NMR spectrum (101 MHz, DMSO-d$_6$)

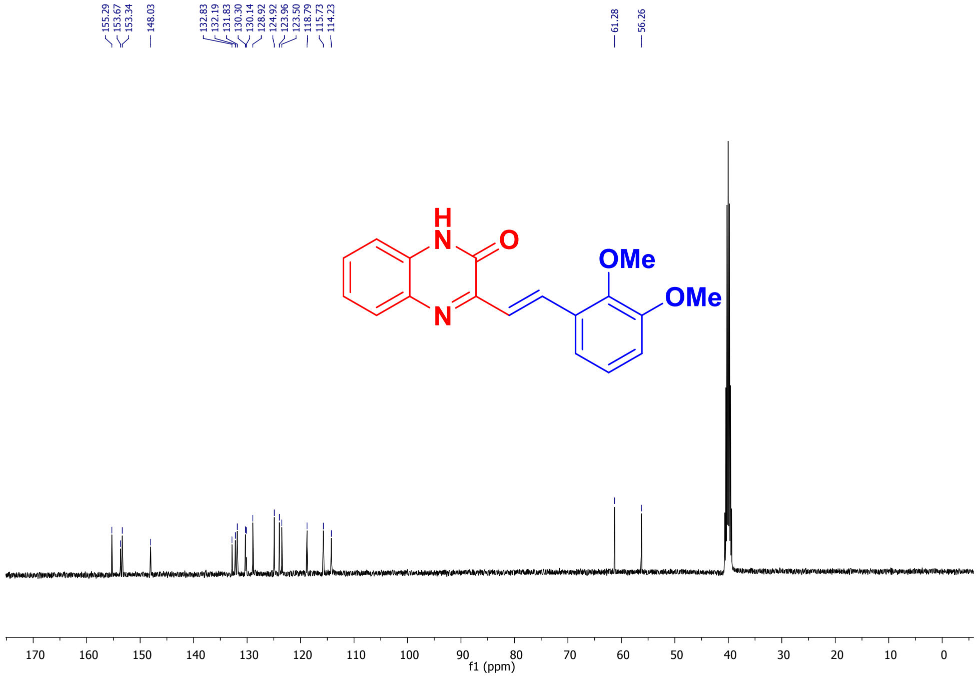

4e. ***(E)-3-(3,4-dimethoxystyryl)quinoxalin-2(1H)-one***

^{1}H NMR spectrum (400 MHz, DMSO-d$_6$)

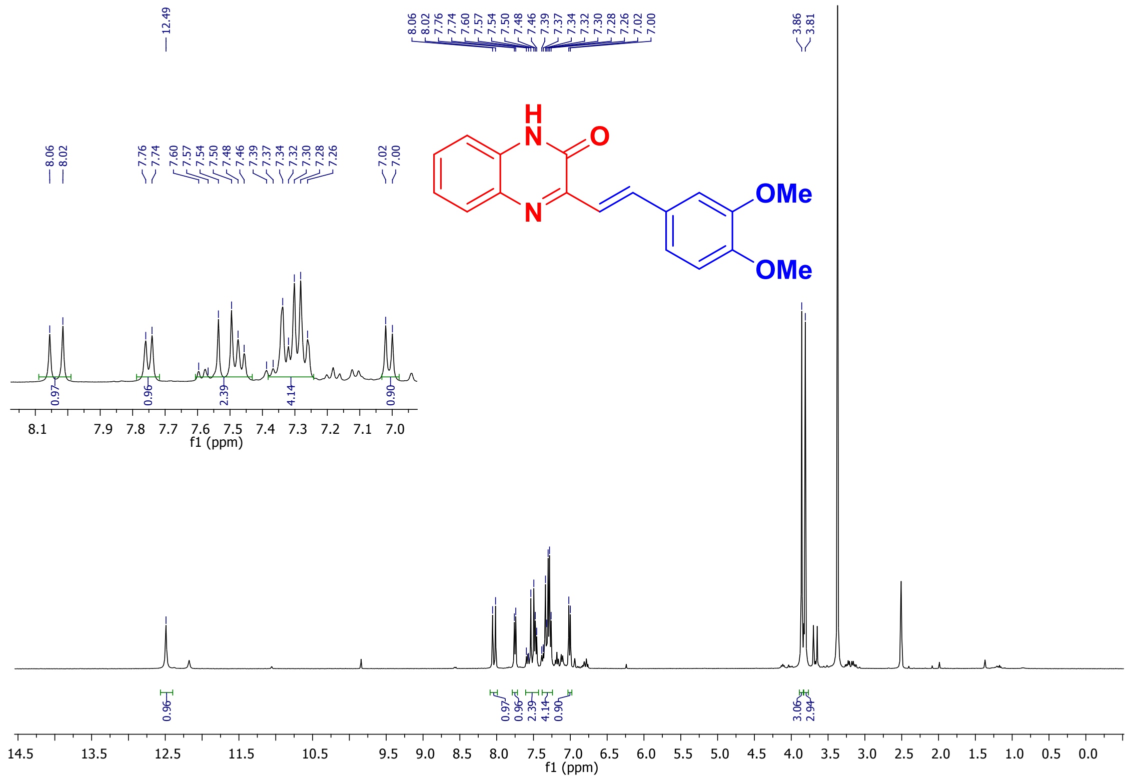

^{13}C NMR spectrum (101 MHz, DMSO-d$_6$)

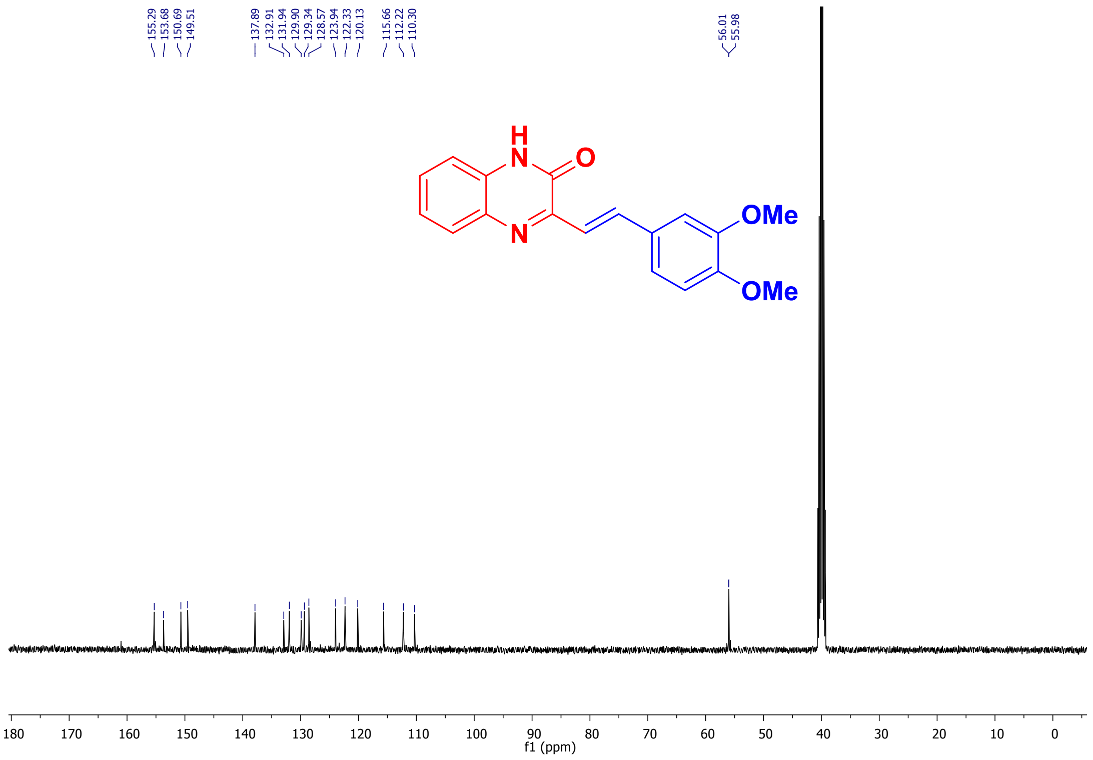

4f. ***(E)-3-(4-methoxystyryl)-6-nitroquinoxalin-2(1H)-one***

^{1}H NMR spectrum (400 MHz, DMSO-d$_6$)

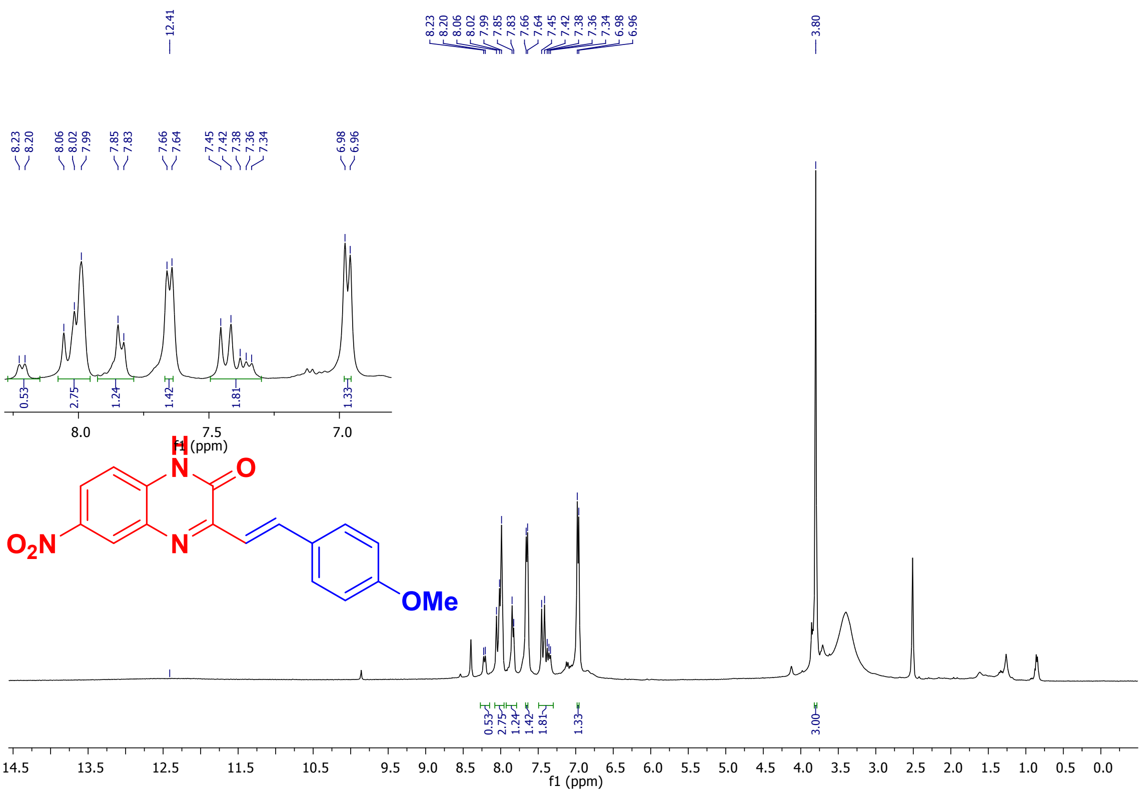

^{13}C NMR spectrum (101 MHz, DMSO-d$_6$)

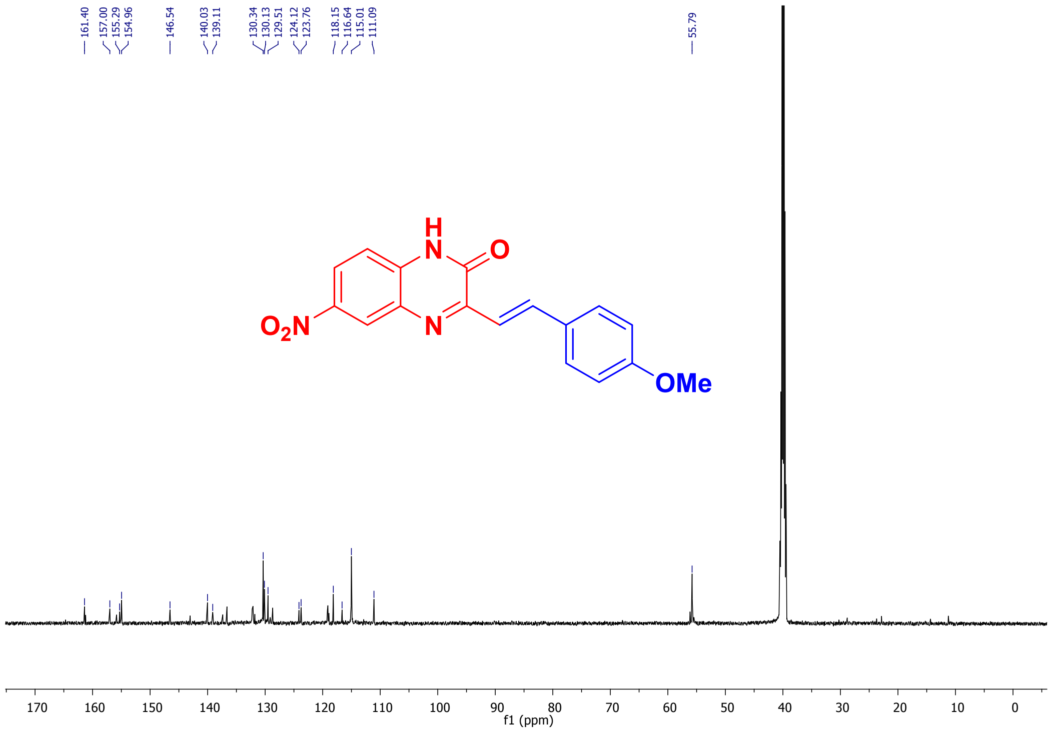

HPLC Analysis of **4f**

The HPLC purity was checked using Shimadzu HPLC system, consisting of purosphere C_{18} (5 µ, 250 × 4.6 mm) column and a PDA detector. The flow rate was 0.6 mL/min with the injection volume of 10 µL. The total run time was 45 min with gradient elution using 0.1% v/v formic acid in water (A) and mobile phase of acetonitrile (B). The gradient (WRT % v/v of A and B) was as shown in the **Table 2** :

Table 2 : Parameters used in HPLC purity check.

Time (in min.)	WRT % v/v of B	WRT % v/v of A
0	0	100
10	10	90
20	30	70
30	60	40
35	80	20
40	0	100
45	Stop	Stop

==== Shimadzu LCsolution Analysis Report ====

C:\newcgmp\purity\4F-01.lcd

Acquired by : Admin
Sample Name : 4F-01
Sample ID : 4F-01
Tray# : 1
Vail # : 21
Injection Volume : 5 uL
Data File Name : 4F-01.lcd
Method File Name : purity.lcm
Batch File Name : nancy compound purity.lcb
Report File Name : Default.lcr
Data Acquired : 25-11-2019 19:33:40
Data Processed : 25-11-2019 20:18:43

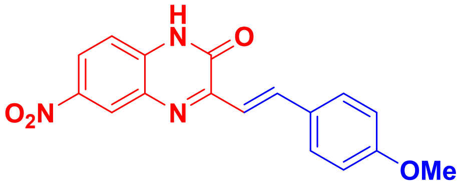

<Chromatogram>

C:\newcgmp\purity\4F-01.lcd

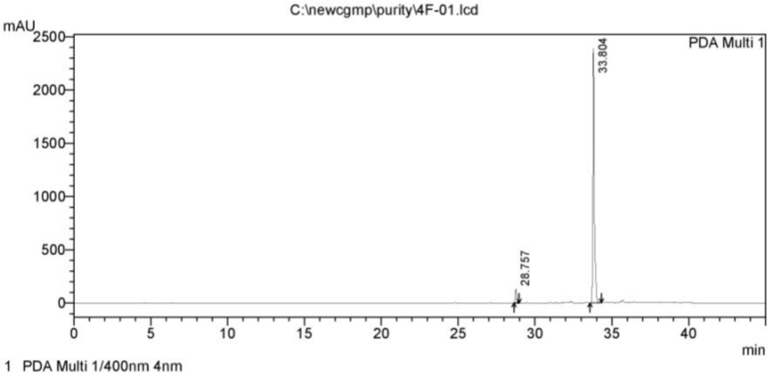

1 PDA Multi 1/400nm 4nm

PeakTable

PDA Ch1 400nm 4nm

Peak#	Ret. Time	Area	Height	Area %	Height %
1	28.757	790676	132179	4.868	5.245
2	33.804	15450172	2387966	95.132	94.755
Total		16240848	2520144	100.000	100.000

HRMS Analysis of **4f**

LC HRMS- THERMOSCIENTIFIC- EXACTIVE
C18 COLUMN- Hypersil
MOBILE PHASE- methanol and water (0.1% formic acid)
Gradient method : 97% methanol and 3% water for 5 minutes.
Injected amount : 2Microlitre
Flow rate of solvent 150µl /minute
The source was operated in both positive and negative mode at an ion spray voltage of 3KV
Oven temperature was set to 30^{0}C

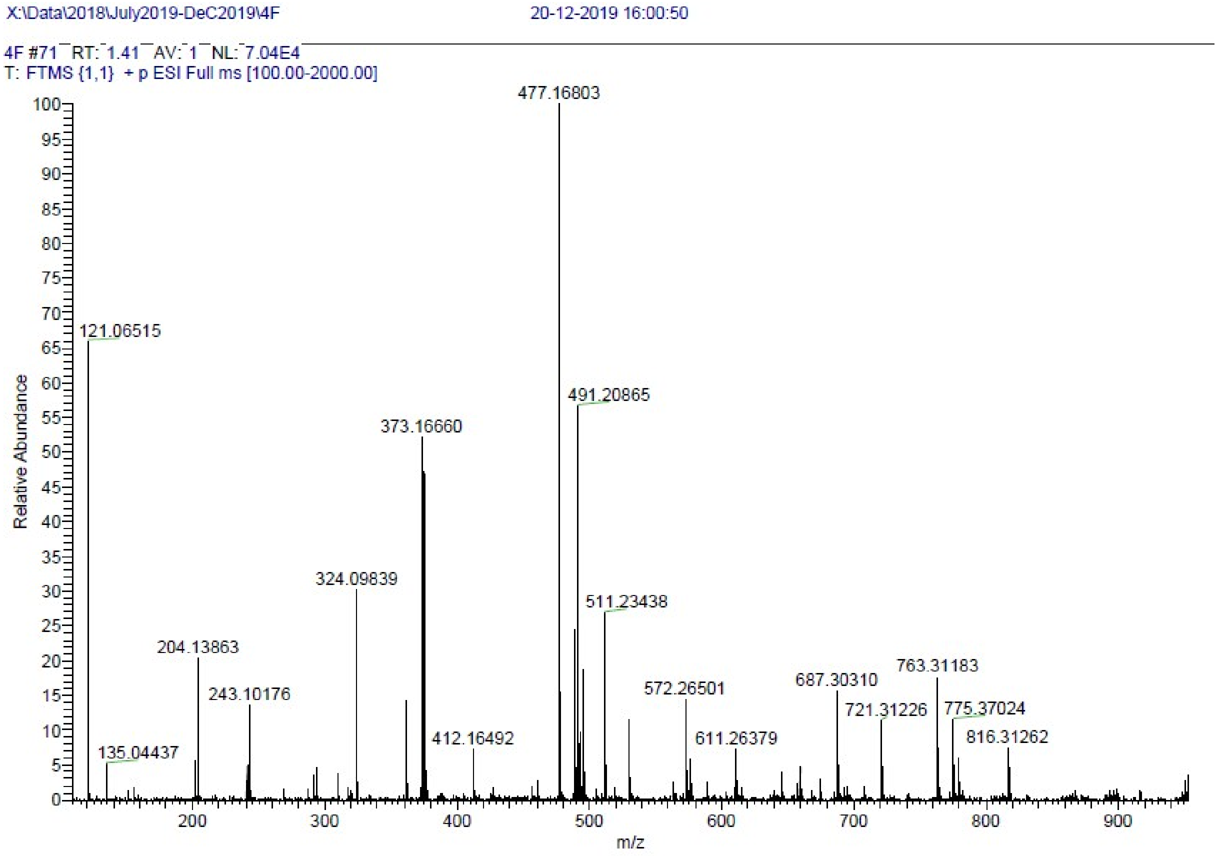

X:\Data\2018\July2019-DeC2019\4F 20-12-2019 16:00:50

4F #71 RT: 1.41 AV: 1 NL: 2.13E4
T: FTMS {1,1} + p ESI Full ms [100.00-2000.00]

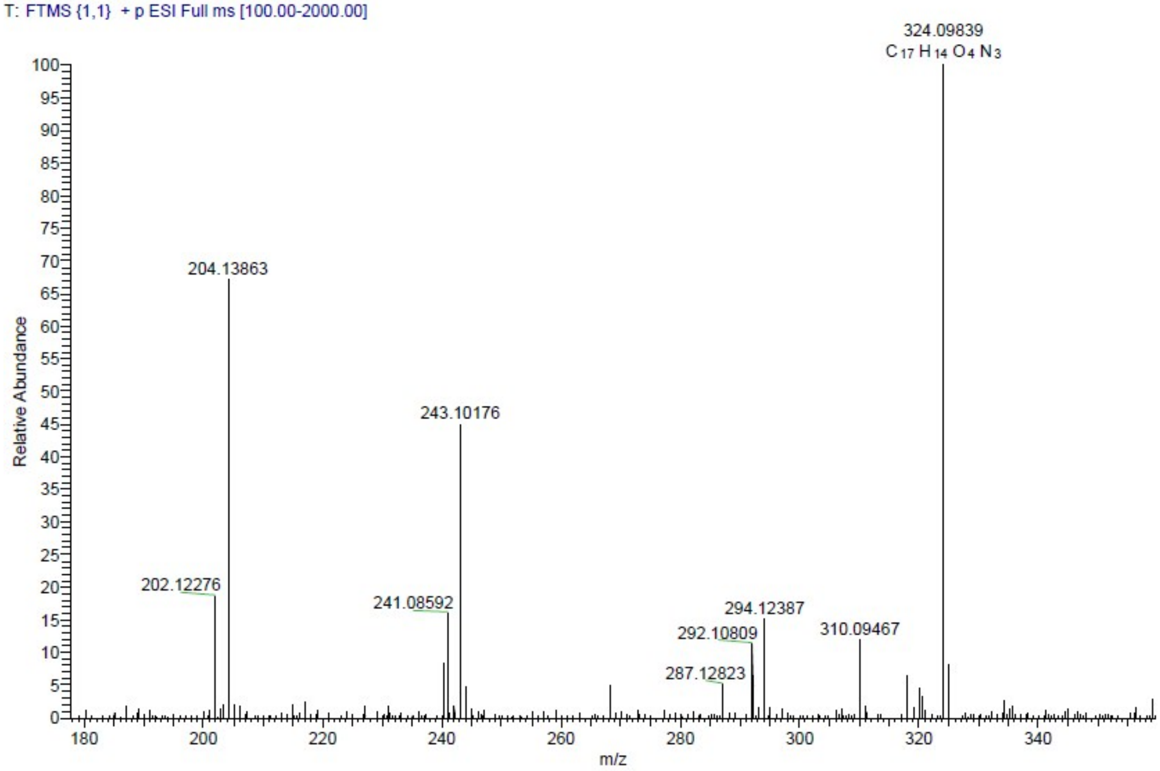

4g. ***(E)-3-(3,4,5-trimethoxystyryl)quinoxalin-2(1H)-one***

^{1}H NMR spectrum (400 MHz, DMSO-d_6)

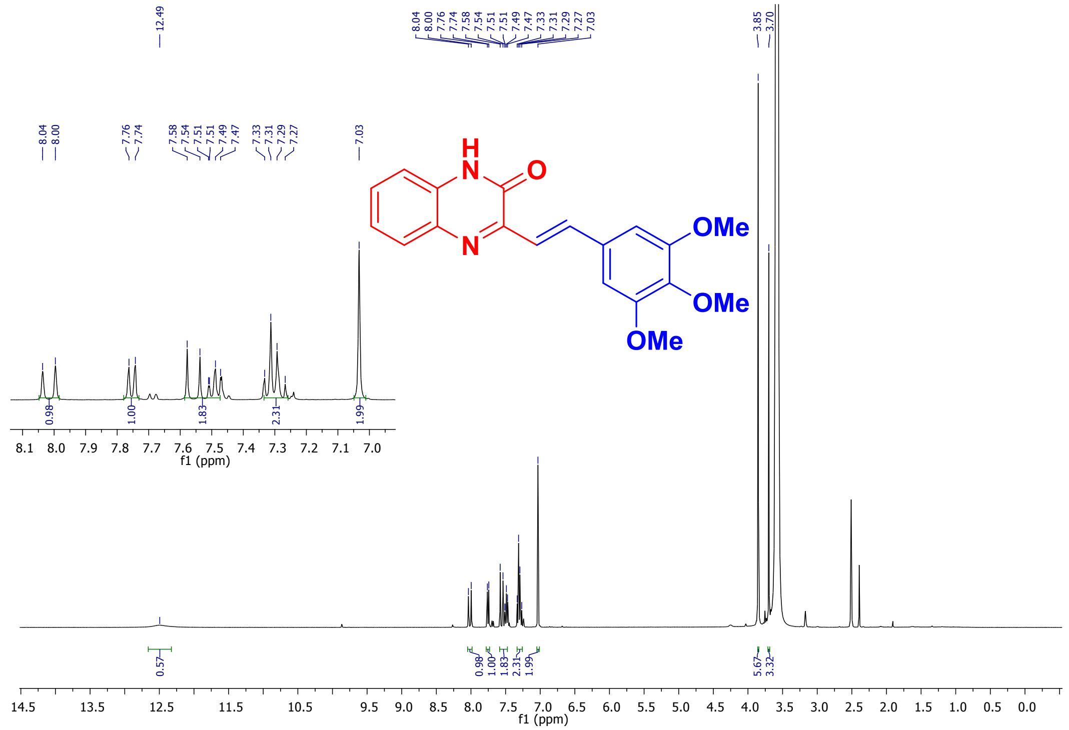

^{13}C NMR spectrum (101 MHz, DMSO-d_6)

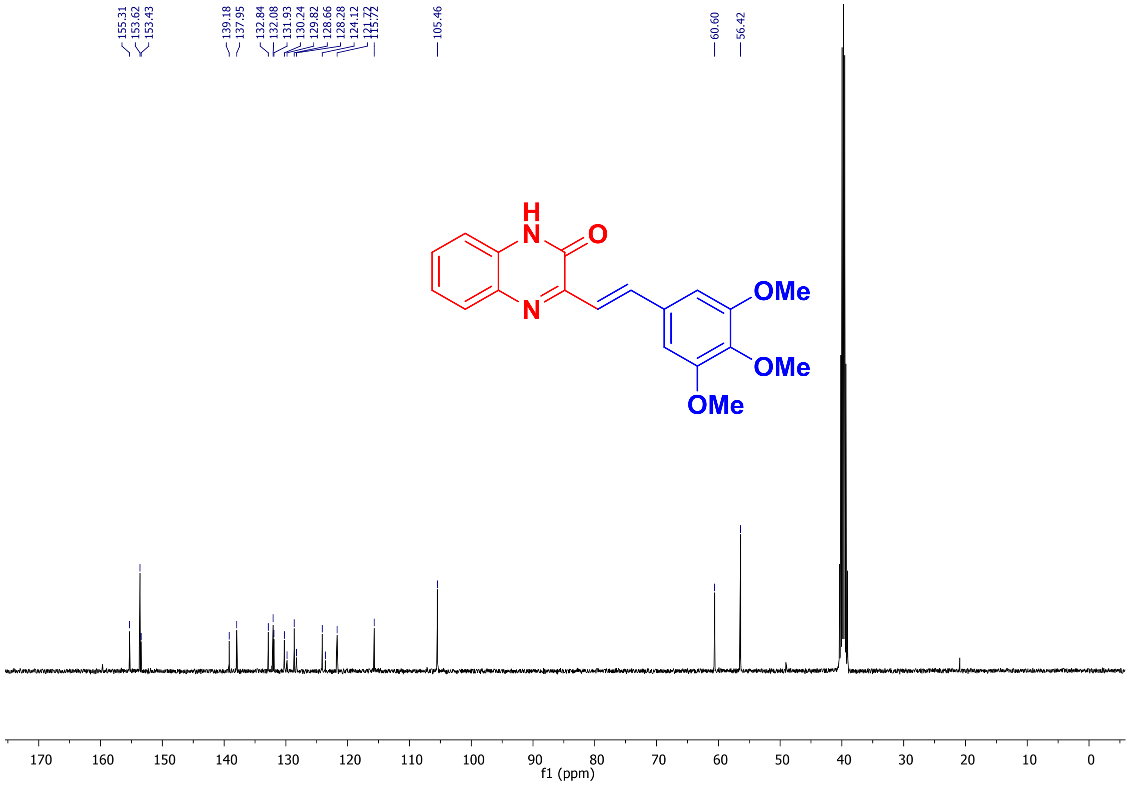

HPLC Analysis of **4g**

The HPLC purity was checked using Shimadzu HPLC system, consisting of purosphere C_{18} (5 µ, 250 × 4.6 mm) column and a PDA detector. The flow rate was 0.6 mL/min with the injection volume of 10 µL. The total run time was 45 min with gradient elution using 0.1% v/v formic acid in water (A) and mobile phase of acetonitrile (B). The gradient (WRT % v/v of A and B) was as shown in the **Table 2** :

Table 2 : Parameters used in HPLC purity check.

**Time (in min.)**	**WRT % v/v of B**	**WRT % v/v of A**
0	0	100
10	10	90
20	30	70
30	60	40
35	80	20
40	0	100
45	Stop	Stop

==== Shimadzu LCsolution Analysis Report ====

C:\newcgmp\purity\4G-01.lcd

Acquired by	: Admin
Sample Name	: 4G
Sample ID	: 4G-01
Tray#	: 1
Vail #	: 22
Injection Volume	: 5 uL
Data File Name	: 4G-01.lcd
Method File Name	: purity.lcm
Batch File Name	: nancy compound purity.lcb
Report File Name	: Default.lcr
Data Acquired	: 28-11-2019 20:13:52
Data Processed	: 29-11-2019 11:15:57

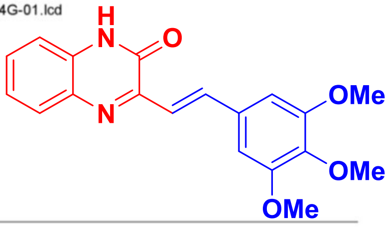

<Chromatogram>

C:\newcgmp\purity\4G-01.lcd

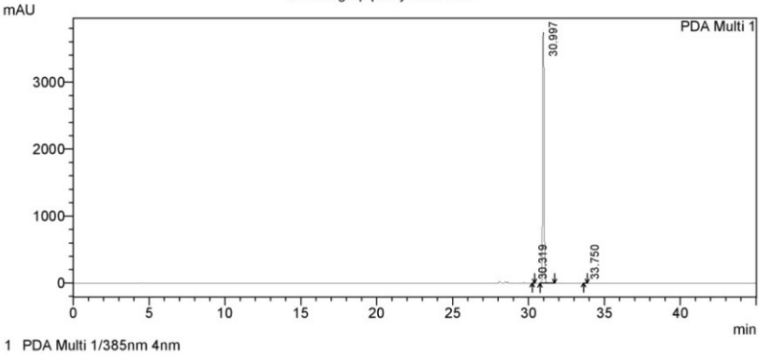

1 PDA Multi 1/385nm 4nm

PeakTable

PDA Ch1 385nm 4nm

Peak#	Ret. Time	Area	Height	Area %	Height %
1	30.319	31275	6360	0.118	0.169
2	30.997	26328342	3744751	99.714	99.643
3	33.750	44270	7042	0.168	0.187
Total		26403888	3758153	100.000	100.000

HRMS Analysis of **4g**

LC HRMS- THERMOSCIENTIFIC- EXACTIVE
C18 COLUMN- Hypersil
MOBILE PHASE- methanol and water (0.1% formic acid)
Gradient method : 97% methanol and 3% water for 5 minutes.
Injected amount : 2Microlitre
Flow rate of solvent 150µl /minute
The source was operated in both positive and negative mode at an ion spray voltage of 3KV
Oven temperature was set to 30^{0}C

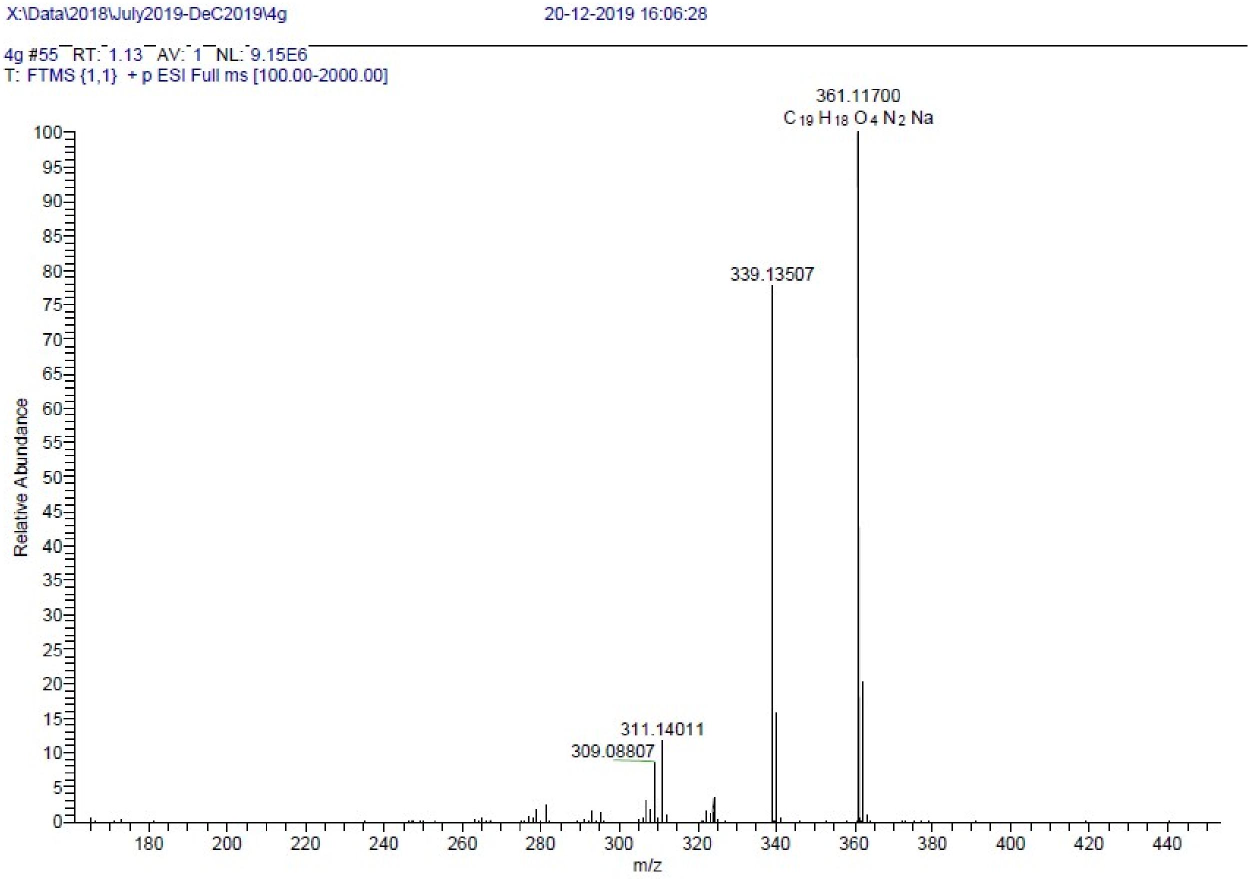

4h. ***(E)-3-(2-chlorostyryl)quinoxalin-2(1H)-one***

^{1}H NMR spectrum (400 MHz, DMSO-d_6)

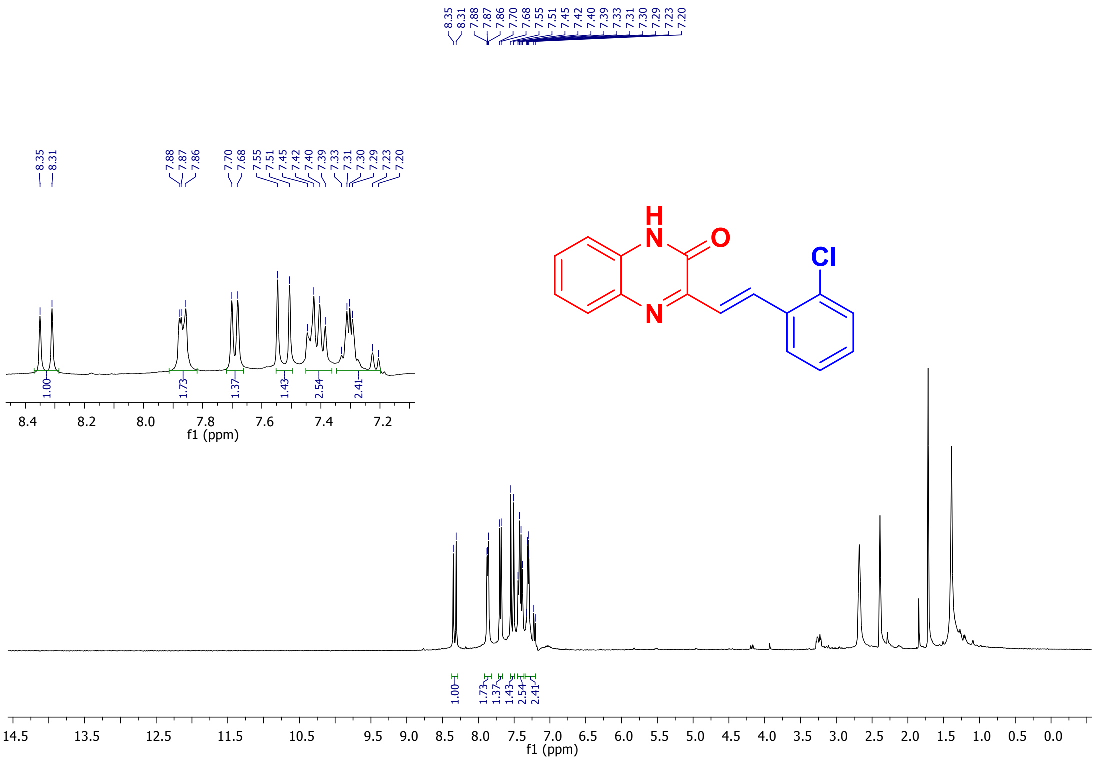

^{13}C NMR spectrum (101 MHz, DMSO-d_6)

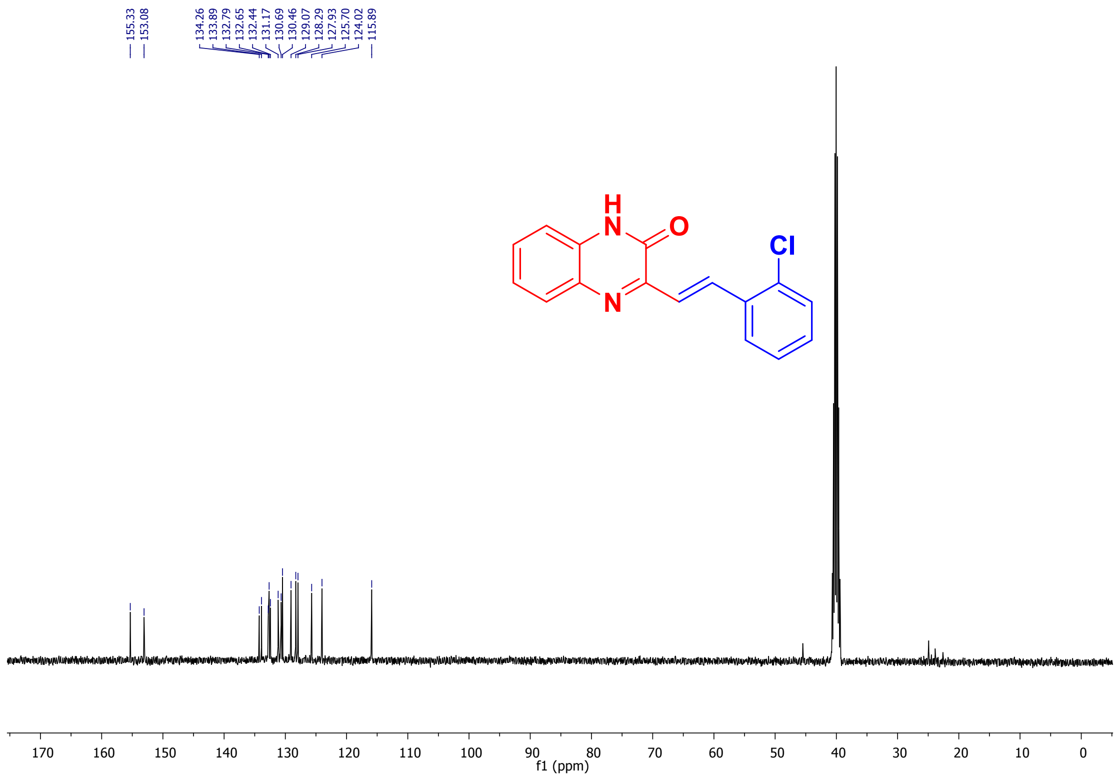

4i. *(E)-3-(4-chlorostyryl)quinoxalin-2(1H)-one*

<u>^{1}H NMR spectrum</u> (400 MHz, DMSO-d_6)

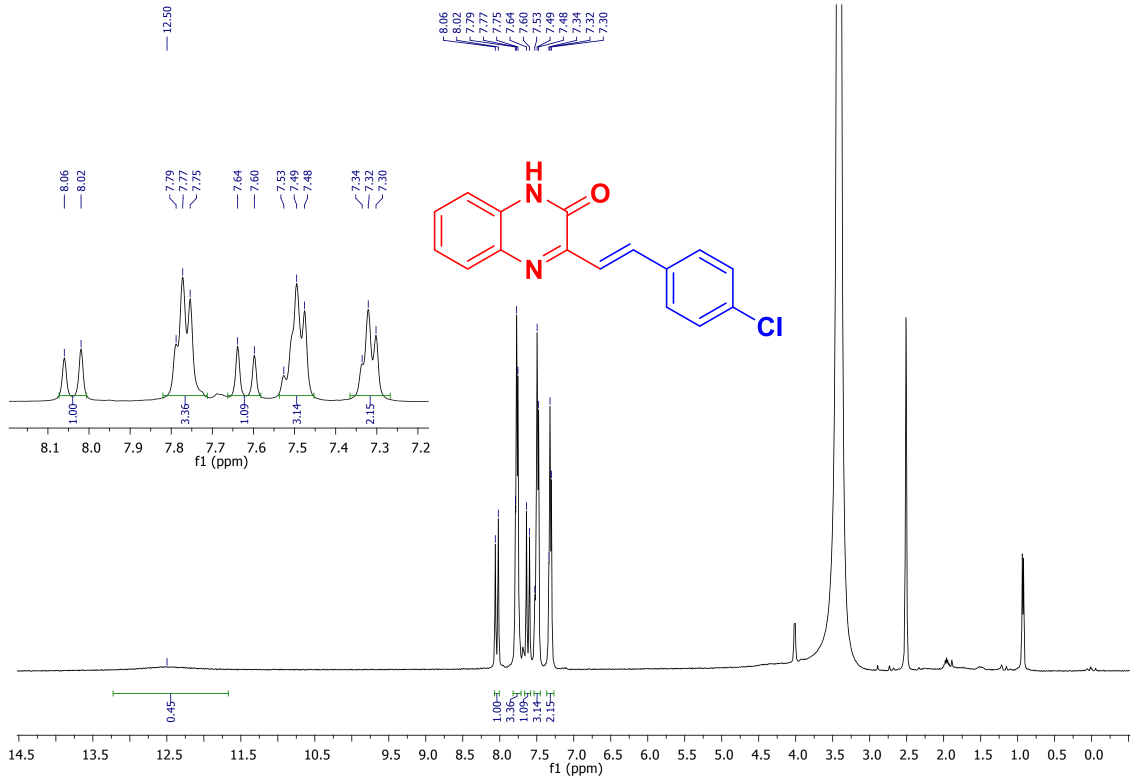

<u>^{13}C NMR spectrum</u> (101 MHz, DMSO-d_6)

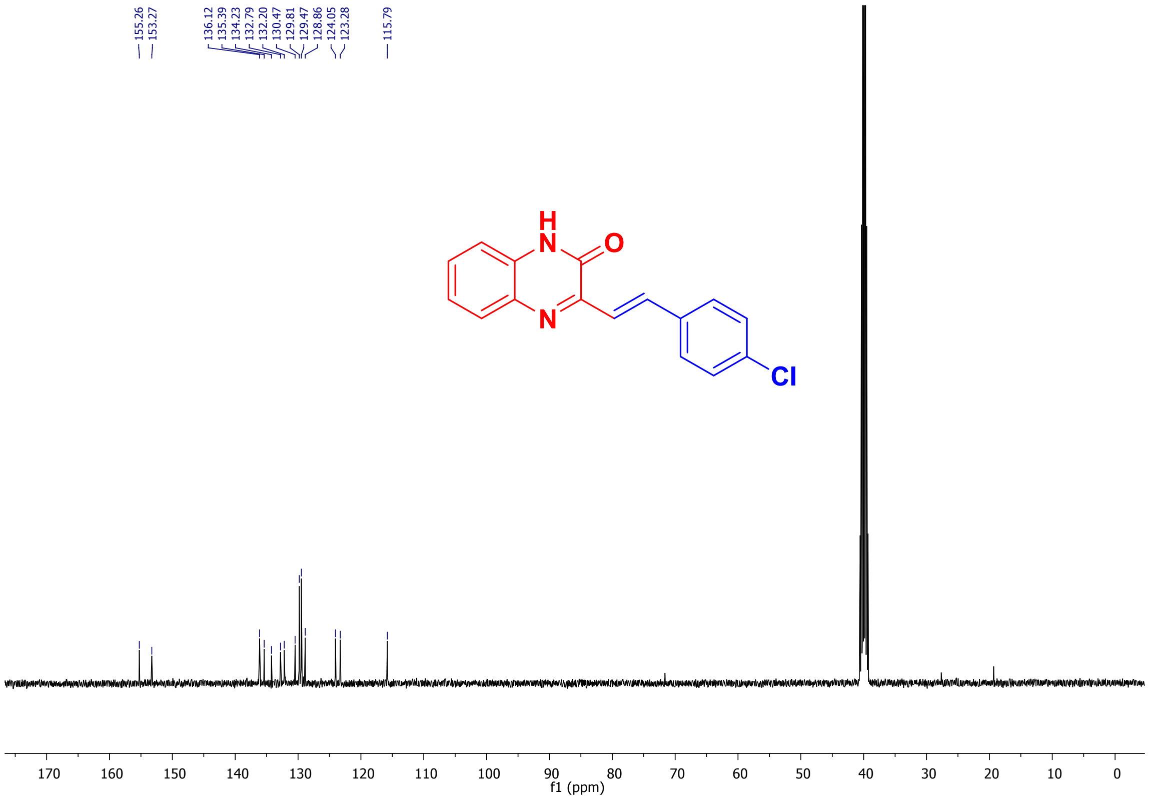

4j. ***(E)-3-(4-chlorostyryl)-6-nitroquinoxalin-2(1H)-one***

<u>^{1}H NMR spectrum</u> (400 MHz, DMSO-d$_6$)

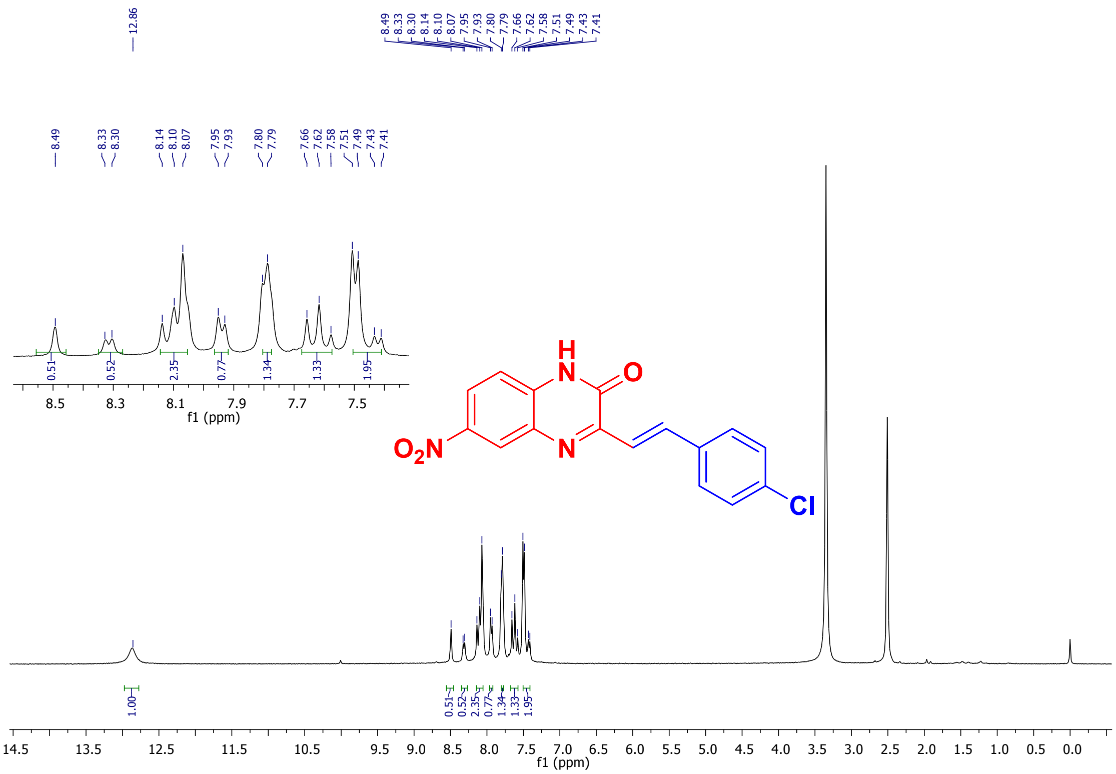

<u>^{13}C NMR spectrum</u> (101 MHz, DMSO-d$_6$)

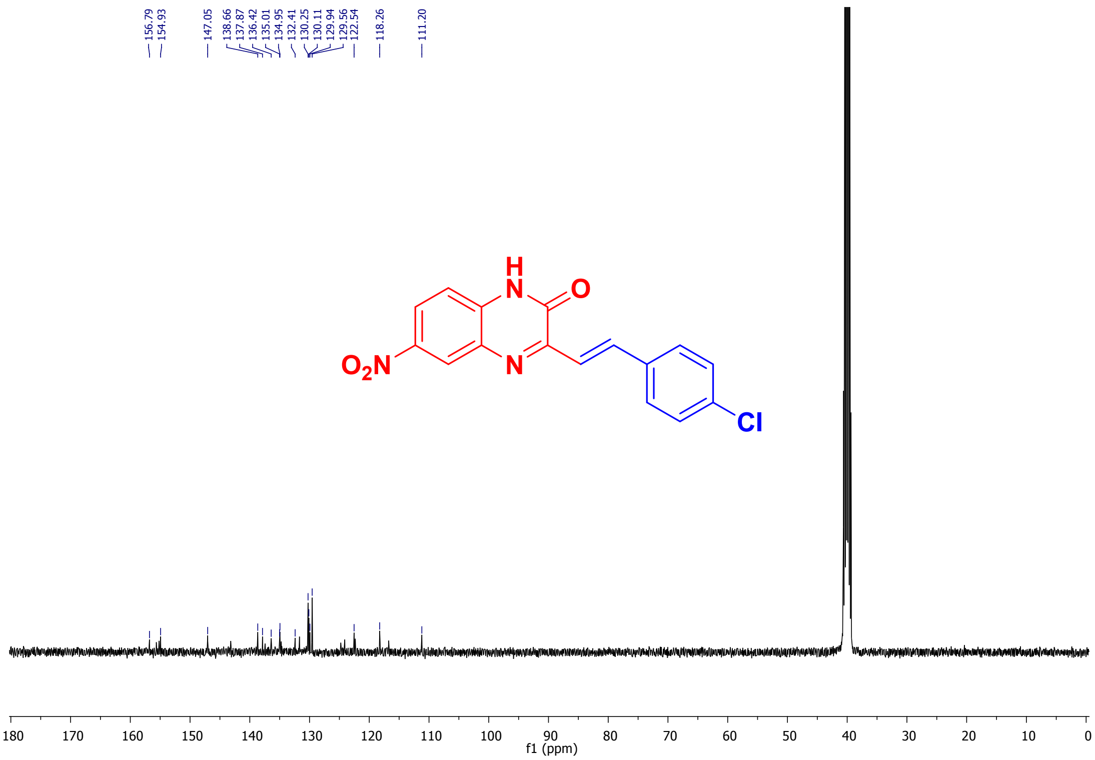

HPLC Analysis of **4j**

The HPLC purity was checked using Shimadzu HPLC system, consisting of purosphere C_{18} (5 µ, 250 × 4.6 mm) column and a PDA detector. The flow rate was 0.6 mL/min with the injection volume of 10 µL. The total run time was 45 min with gradient elution using 0.1% v/v formic acid in water (A) and mobile phase of acetonitrile (B). The gradient (WRT % v/v of A and B) was as shown in the **Table 2** :

Table 2 : Parameters used in HPLC purity check.

**Time (in min.)**	**WRT % v/v of B**	**WRT % v/v of A**
0	0	100
10	10	90
20	30	70
30	60	40
35	80	20
40	0	100
45	Stop	Stop

26-11-2019 10:34:03 1 / 1

==== Shimadzu LCsolution Analysis Report ====

C:\newcgmp\purity\4J.lcd

Acquired by	: Admin
Sample Name	: 4J
Sample ID	: 4J
Tray#	: 1
Vail #	: 23
Injection Volume	: 5 uL
Data File Name	: 4J.lcd
Method File Name	: purity.lcm
Batch File Name	: nancy compound purity.lcb
Report File Name	: Default.lcr
Data Acquired	: 25-11-2019 21:04:42
Data Processed	: 25-11-2019 21:49:44

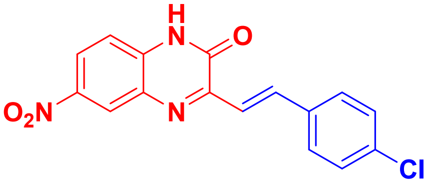

<Chromatogram>

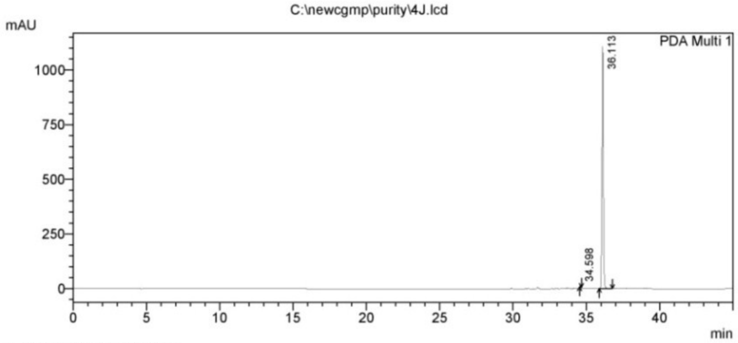

1 PDA Multi 1/380nm 4nm

PeakTable

PDA Ch1 380nm 4nm

Peak#	Ret. Time	Area	Height	Area %	Height %
1	34.598	48569	11705	0.649	1.046
2	36.113	7436145	1106880	99.351	98.954
Total		7484715	1118585	100.000	100.000

HRMS Analysis of **4j**

LC HRMS- THERMOSCIENTIFIC- EXACTIVE
C18 COLUMN- Hypersil
MOBILE PHASE- methanol and water (0.1% formic acid)
Gradient method : 97% methanol and 3% water for 5 minutes.
Injected amount : 2Microlitre
Flow rate of solvent 150µl /minute
The source was operated in both positive and negative mode at an ion spray voltage of 3KV
Oven temperature was set to 30^{0}C

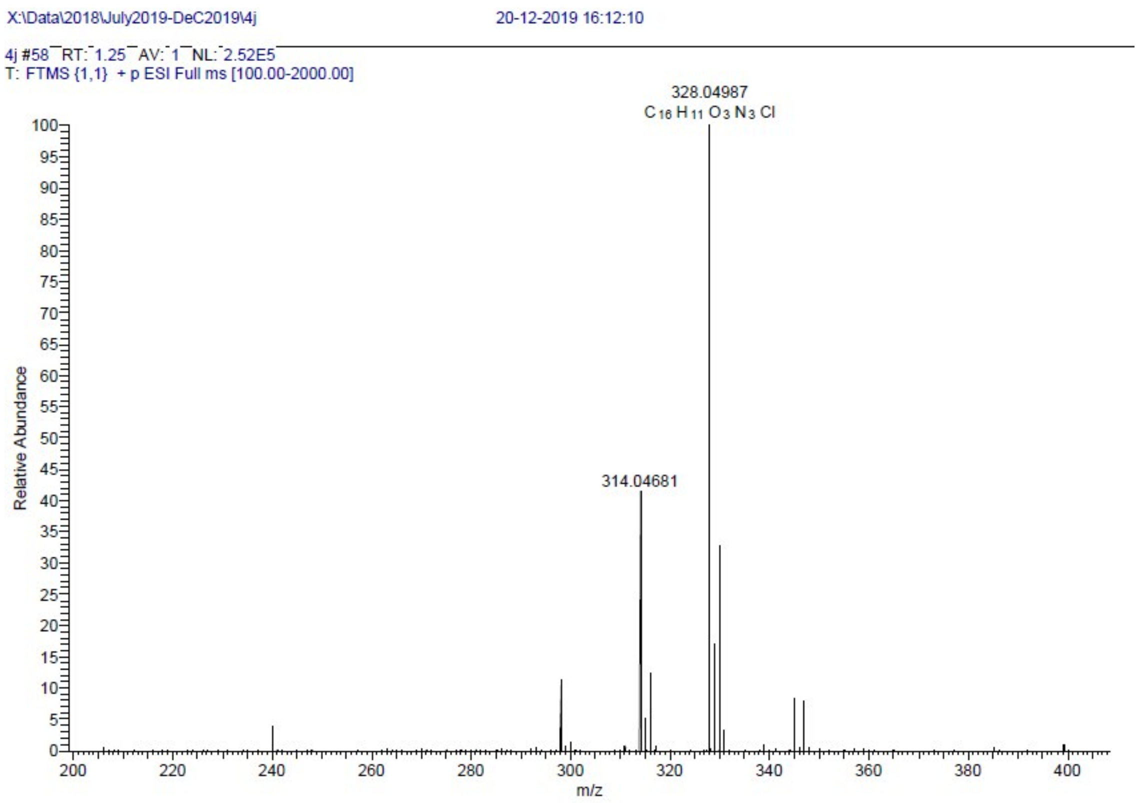

X:\Data\2018\July2019-DeC2019\4j 20-12-2019 16:12:10

4j #58 RT: 1.25 AV: 1 NL: 2.52E5
T: FTMS {1,1} + p ESI Full ms [100.00-2000.00]

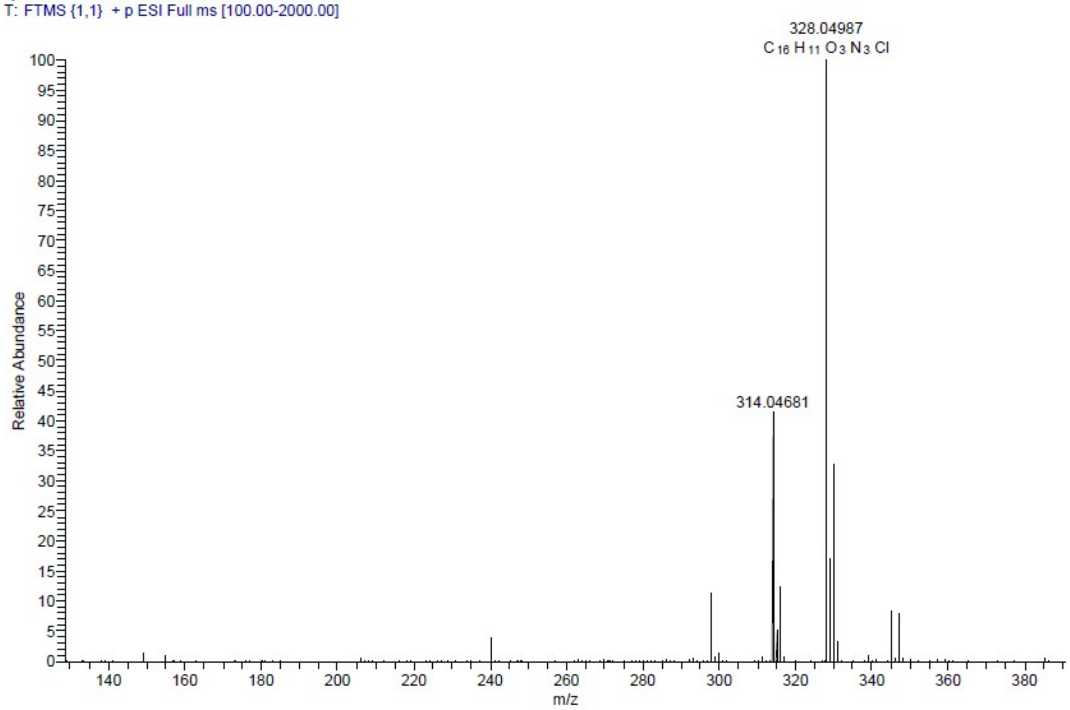

4k. **(*E*)-3-(4-bromostyryl)quinoxalin-2(1*H*)-one**

^{1}H NMR spectrum (400 MHz, DMSO-d_6)

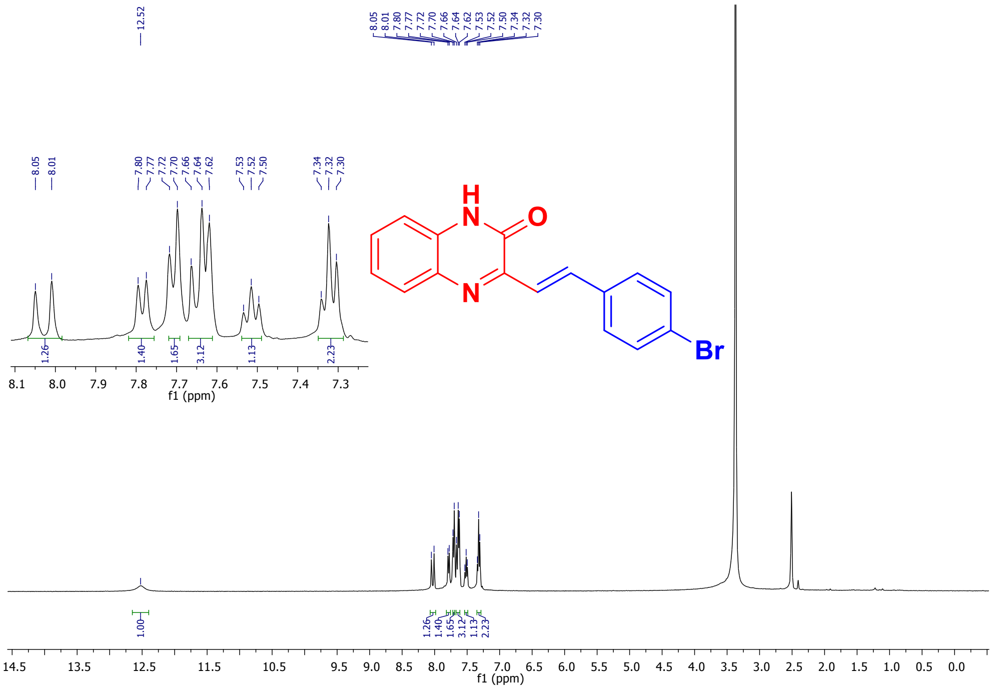

^{13}C NMR spectrum (101 MHz, DMSO-d_6)

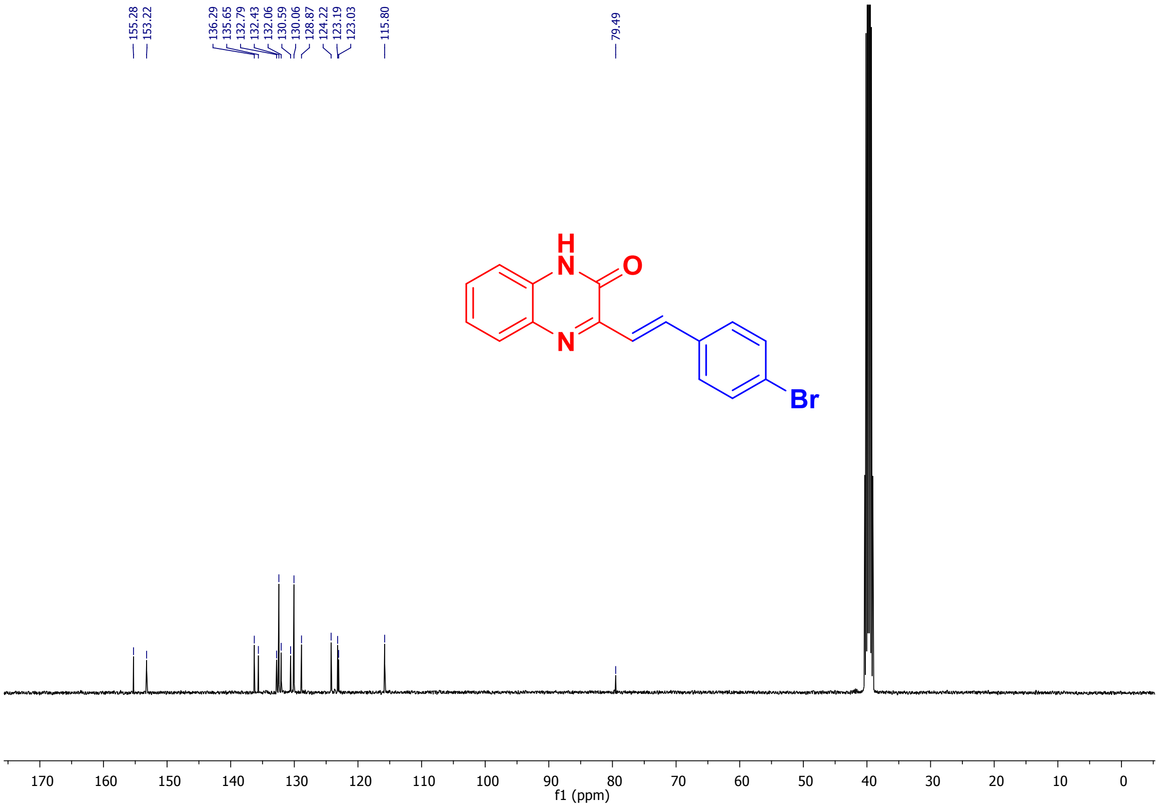

4l. ***(E)-3-(4-(dimethylamino)styryl)quinoxalin-2(1H)-one***

<u>^{1}H NMR spectrum</u> (400 MHz, DMSO-d_6)

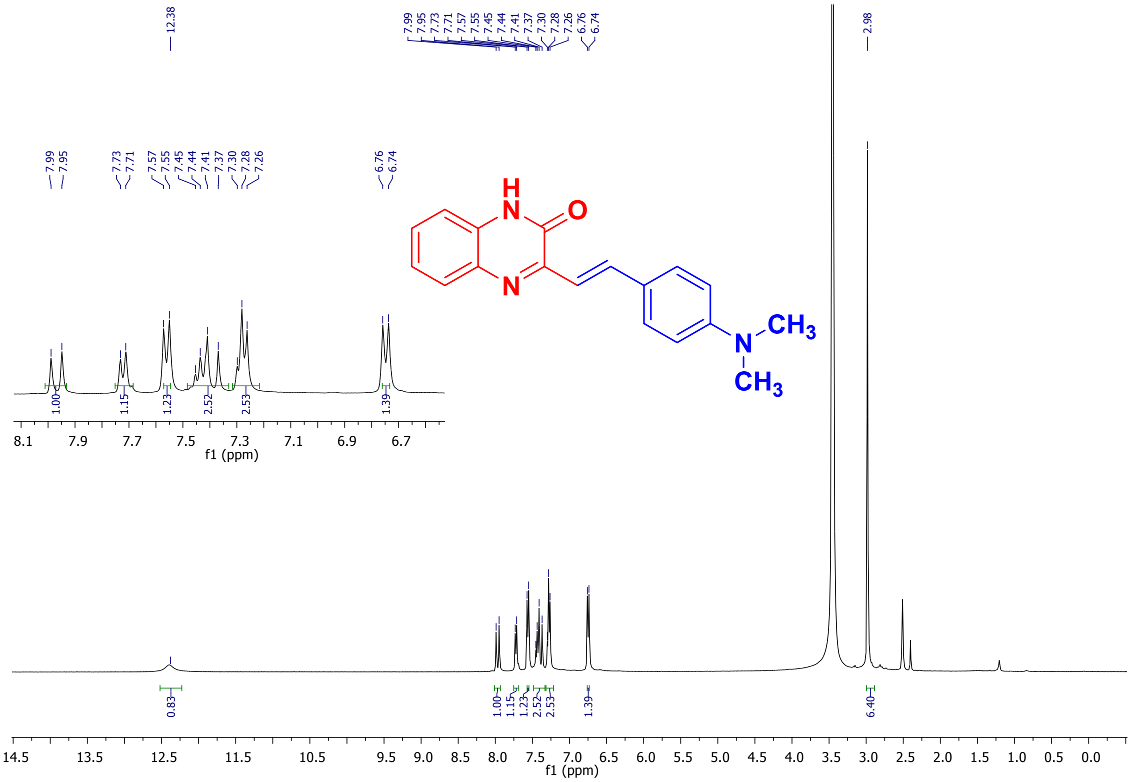

<u>^{13}C NMR spectrum</u> (101 MHz, DMSO-d_6)

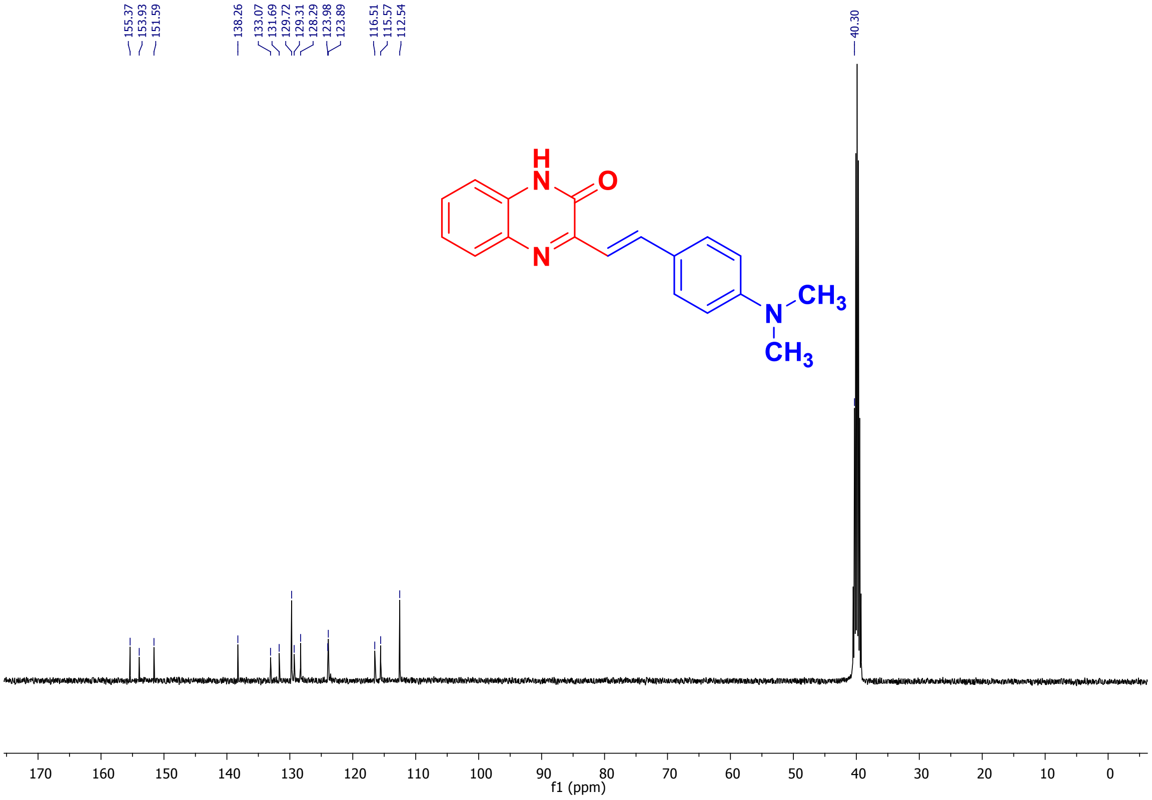

4m. (*E*)-3-(4-nitrostyryl)quinoxalin-2(1H)-one

^{1}H NMR spectrum (400 MHz, DMSO-d$_6$)

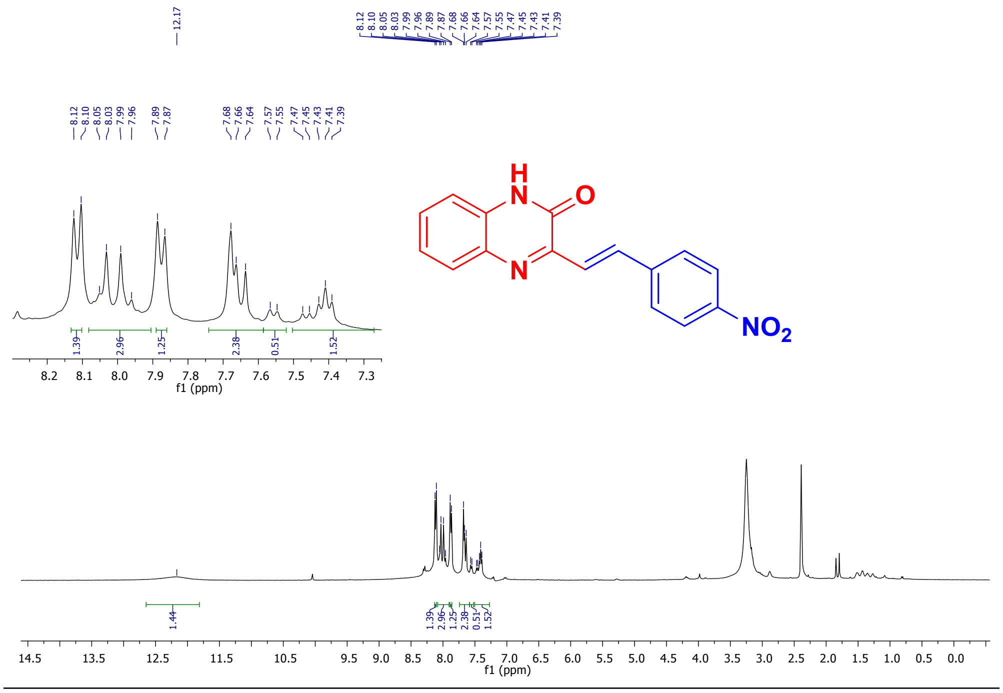

^{13}C NMR spectrum (101 MHz, DMSO-d$_6$)

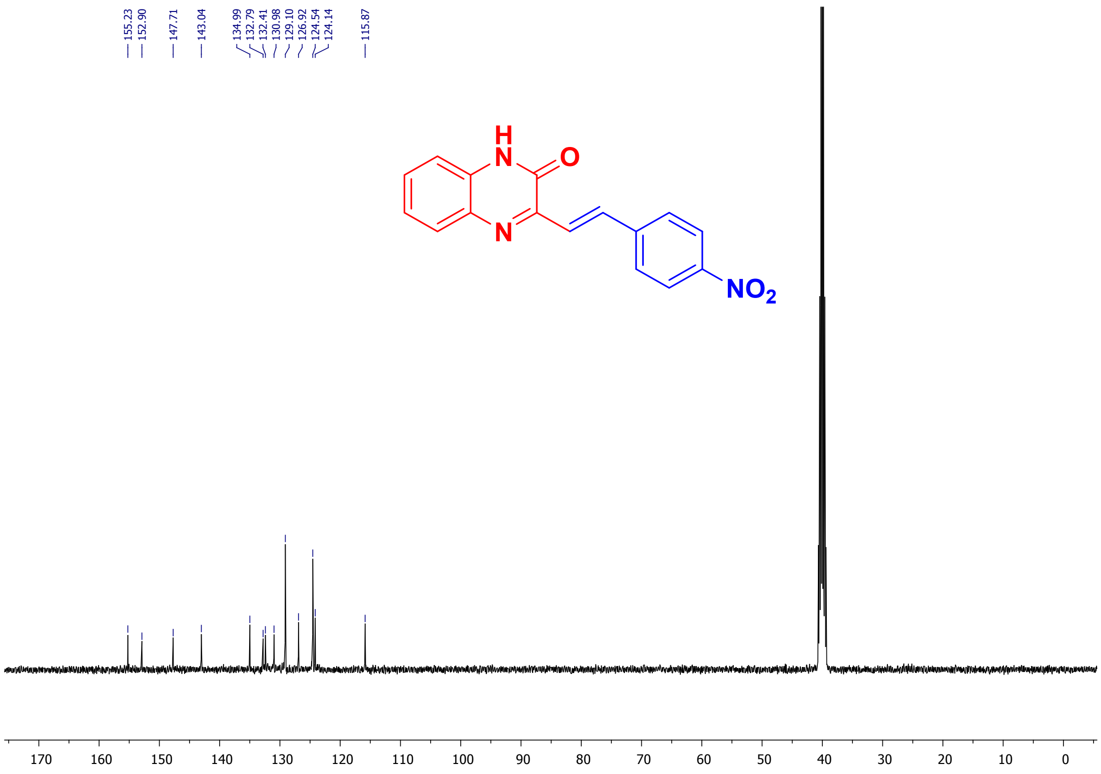

4n. ***(E)-3-(3-bromo-4-fluorostyryl)quinoxalin-2(1H)-one***

^{1}H NMR spectrum (400 MHz, DMSO-d_6)

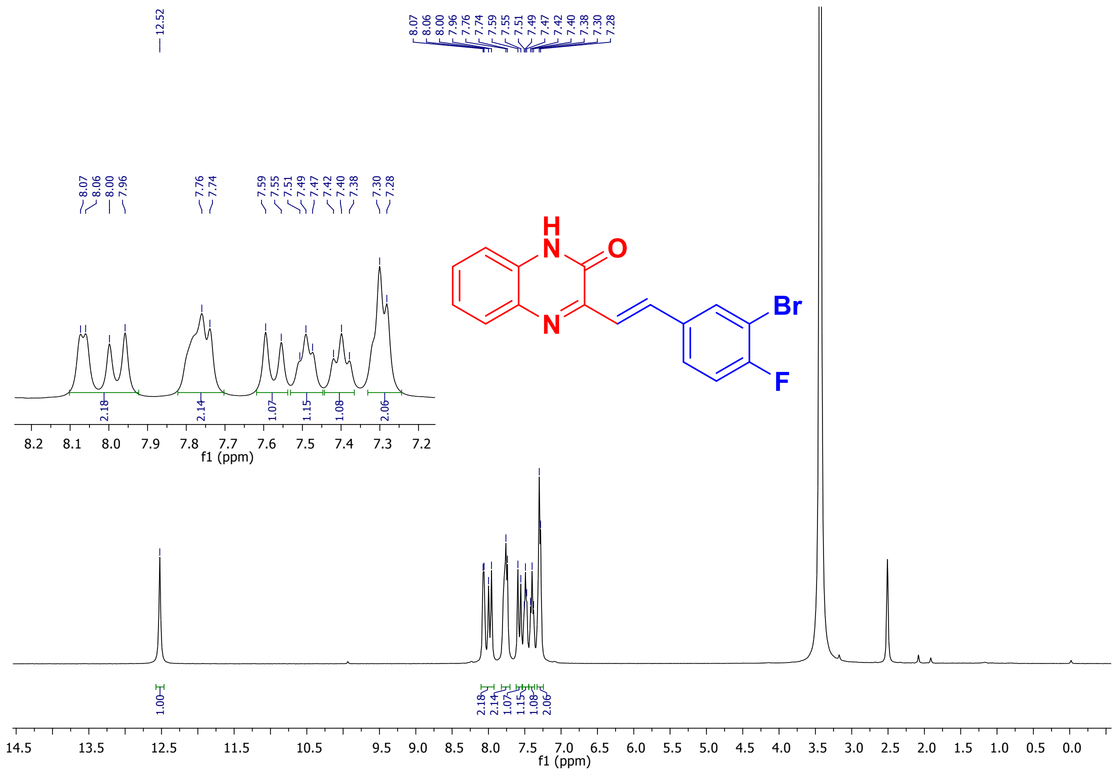

^{13}C NMR spectrum (101 MHz, DMSO-d_6)

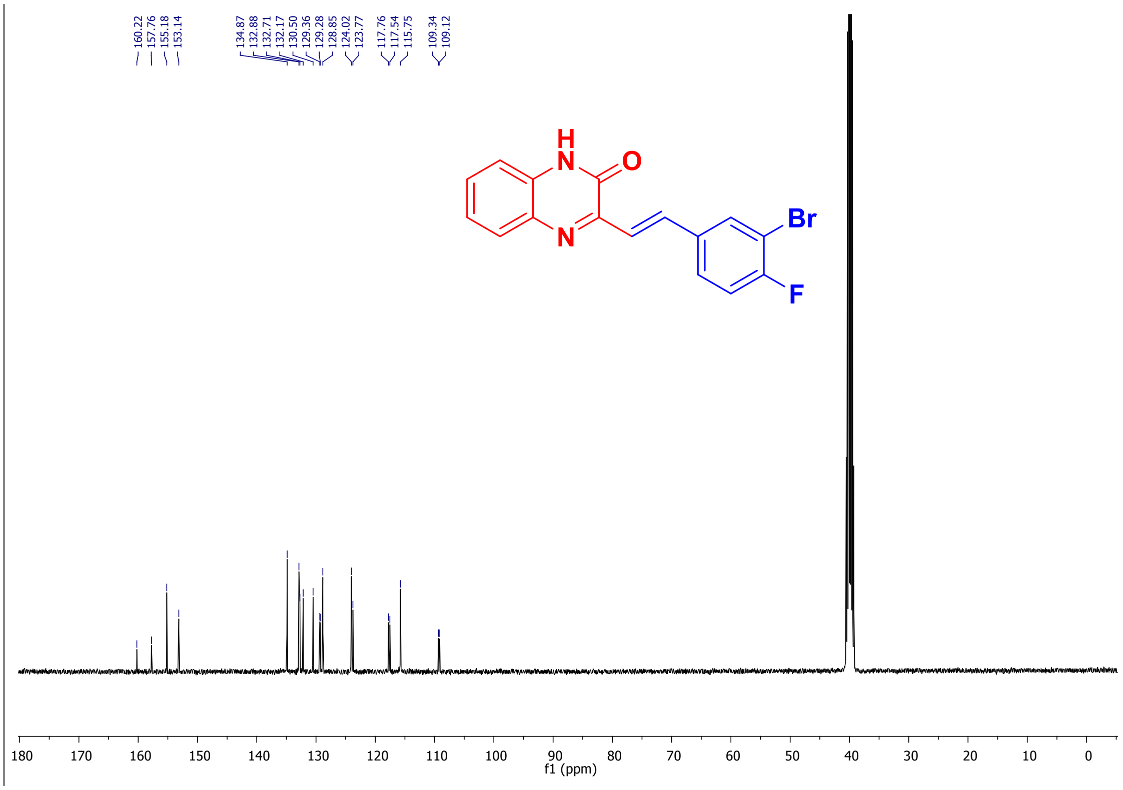

HPLC Analysis of **4n**

The HPLC purity was checked using Shimadzu HPLC system, consisting of purosphere C_{18} (5 µ, 250 × 4.6 mm) column and a PDA detector. The flow rate was 0.6 mL/min with the injection volume of 10 µL. The total run time was 45 min with gradient elution using 0.1% v/v formic acid in water (A) and mobile phase of acetonitrile (B). The gradient (WRT % v/v of A and B) was as shown in the **Table 2** :

Table 2 : Parameters used in HPLC purity check.

Time (in min.)	WRT % v/v of B	WRT % v/v of A
0	0	100
10	10	90
20	30	70
30	60	40
35	80	20
40	0	100
45	Stop	Stop

==== Shimadzu LCsolution Analysis Report ====

C:\newcgmp\purity\4N.lcd

Acquired by : Admin
Sample Name : 4N
Sample ID : 4N
Tray# : 1
Vail # : 24
Injection Volume : 5 uL
Data File Name : 4N.lcd
Method File Name : purity.lcm
Batch File Name : nancy compound purity.lcb
Report File Name : Default.lcr
Data Acquired : 25-11-2019 21:50:10
Data Processed : 25-11-2019 22:35:12

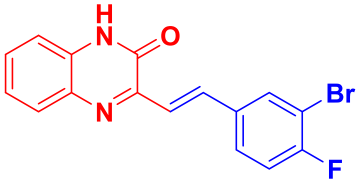

<Chromatogram>

C:\newcgmp\purity\4N.lcd

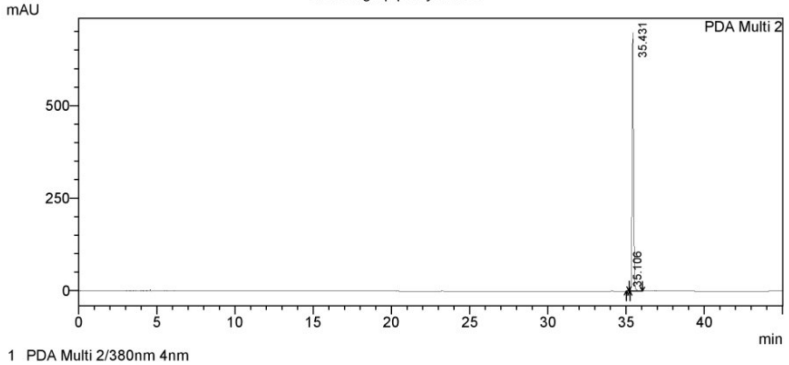

1 PDA Multi 2/380nm 4nm

PeakTable

PDA Ch2 380nm 4nm

Peak#	Ret. Time	Area	Height	Area %	Height %
1	35.106	11021	2103	0.242	0.300
2	35.431	4551543	698094	99.758	99.700
Total		4562564	700198	100.000	100.000

HRMS Analysis of **4n**

LC HRMS- THERMOSCIENTIFIC- EXACTIVE
C18 COLUMN- Hypersil
MOBILE PHASE- methanol and water (0.1% formic acid)
Gradient method : 97% methanol and 3% water for 5 minutes.
Injected amount : 2Microlitre
Flow rate of solvent 150µl /minute
The source was operated in both positive and negative mode at an ion spray voltage of 3KV
Oven temperature was set to 30^{0}C

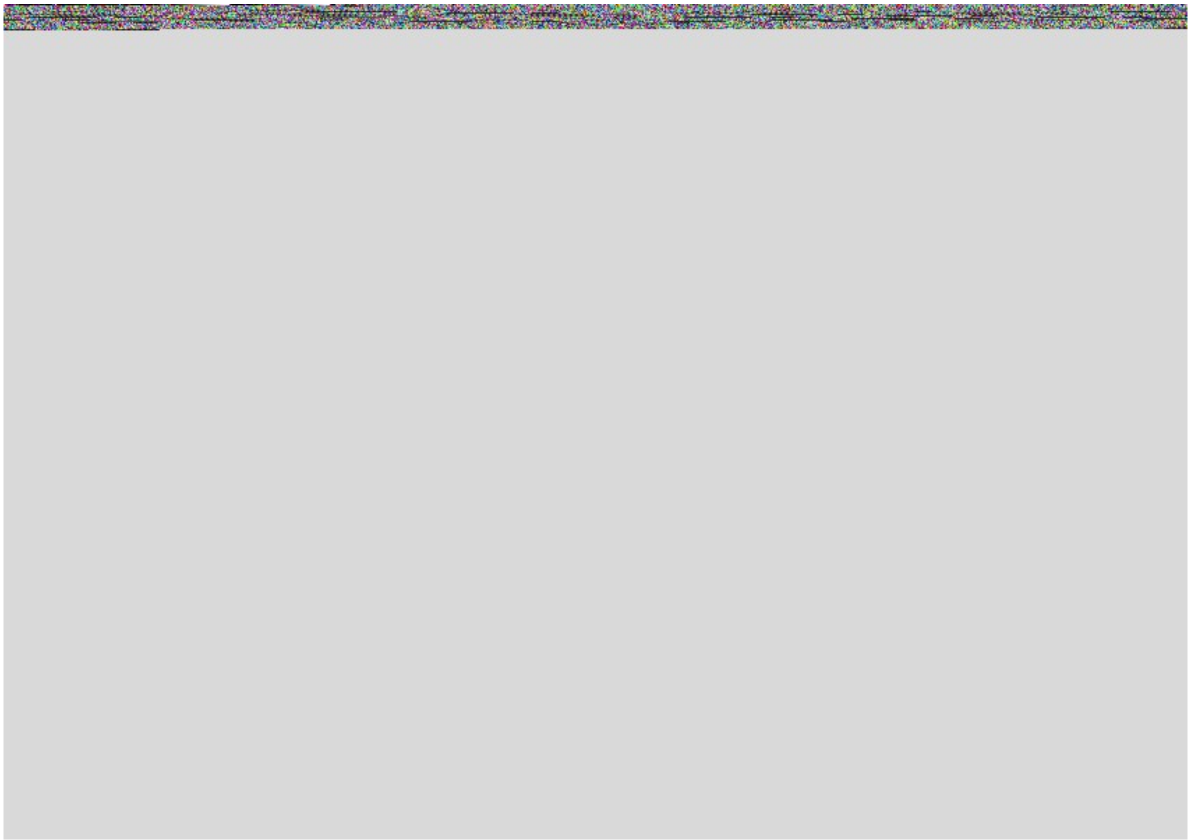

X:\Data\2018\July2019-DeC2019\4n 20-12-2019 16:17:52

4n #61 RT: 1.28 AV: 1 NL: 4.29E6
T: FTMS {1,1} + p ESI Full ms [100.00-2000.00]

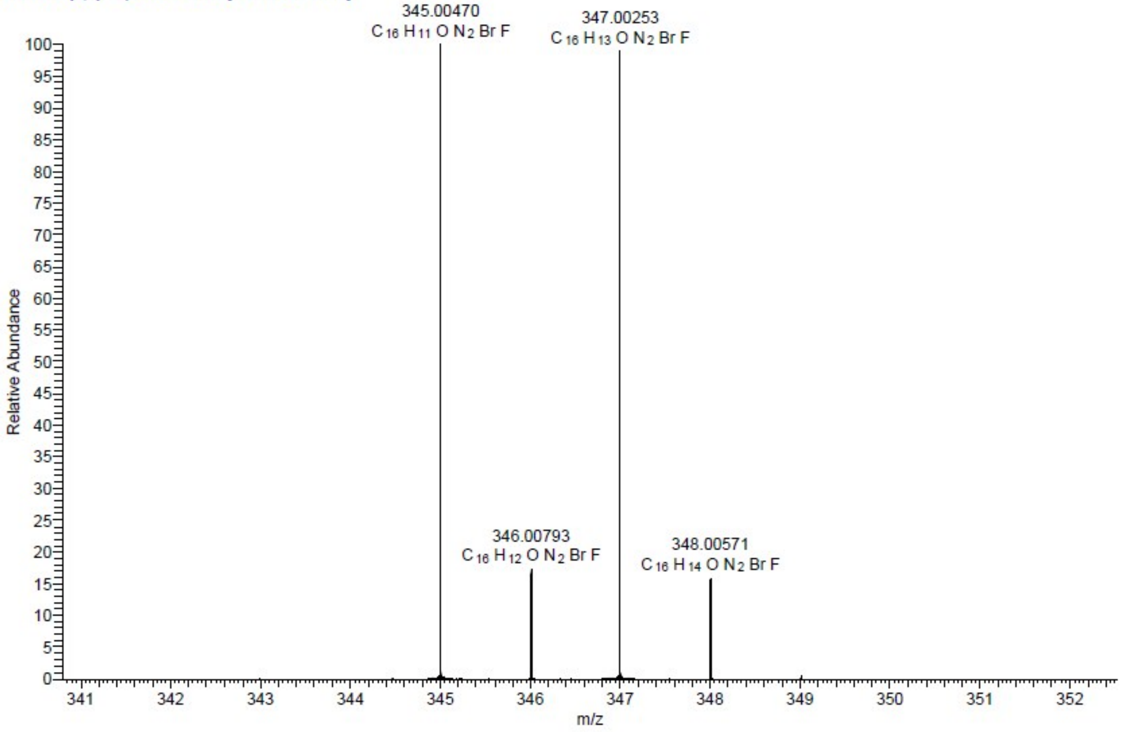

4o. ***(E)-3-(3-bromo-4-methoxystyryl)quinoxalin-2(1H)-one***

^{1}H NMR spectrum (400 MHz, DMSO-d_6)

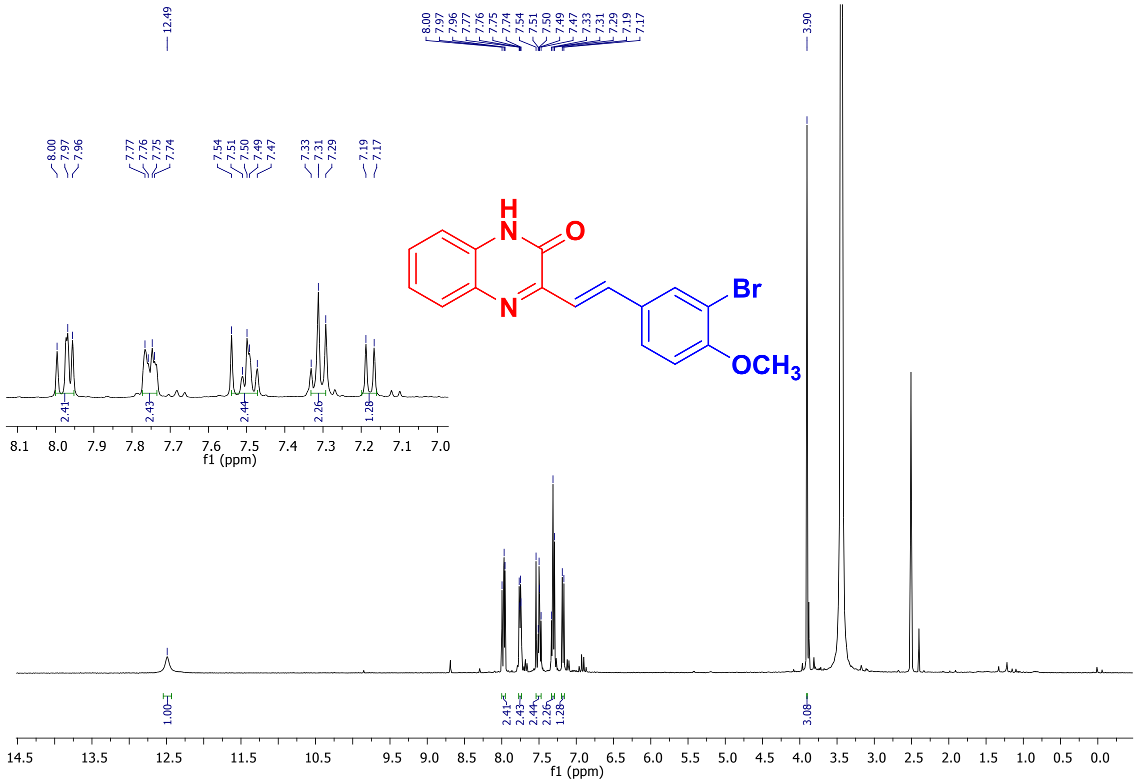

^{13}C NMR spectrum (101 MHz, DMSO-d_6)

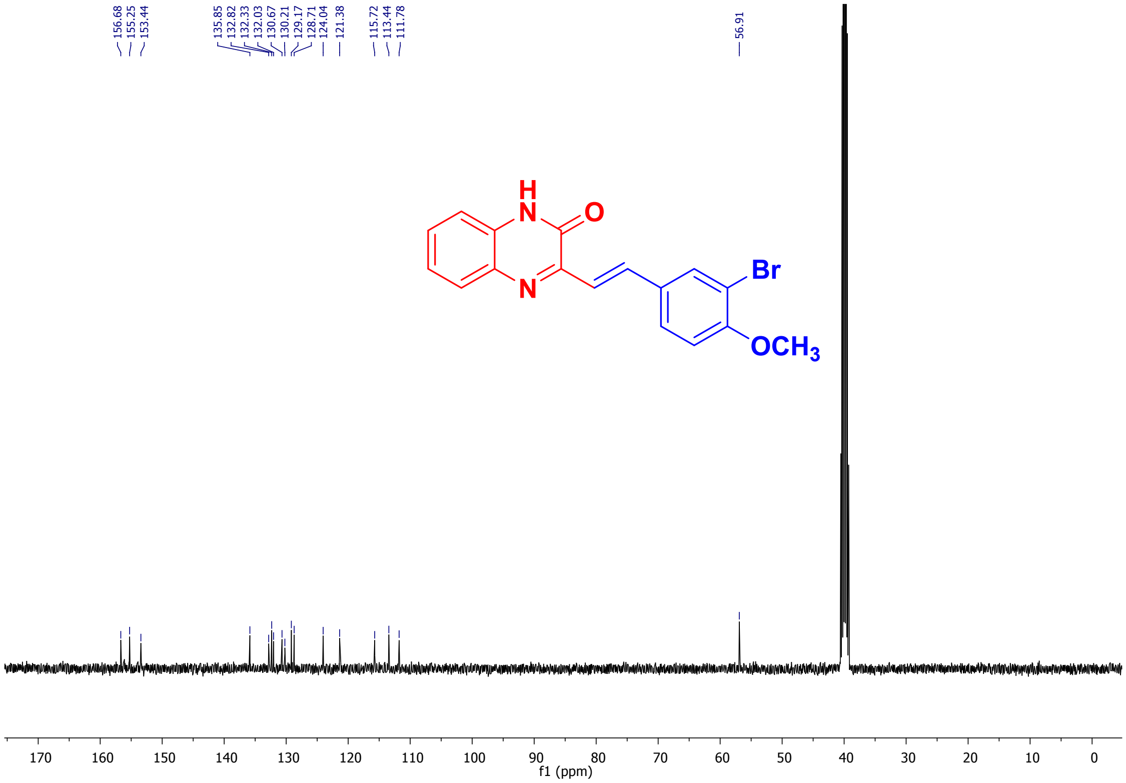

HPLC Analysis of **4o**

The HPLC purity was checked using Shimadzu HPLC system, consisting of purosphere C_{18} (5 µ, 250 × 4.6 mm) column and a PDA detector. The flow rate was 0.6 mL/min with the injection volume of 10 µL. The total run time was 45 min with gradient elution using 0.1% v/v formic acid in water (A) and mobile phase of acetonitrile (B). The gradient (WRT % v/v of A and B) was as shown in the **Table 2** :

Table 2 : Parameters used in HPLC purity check.

Time (in min.)	WRT % v/v of B	WRT % v/v of A
0	0	100
10	10	90
20	30	70
30	60	40
35	80	20
40	0	100
45	Stop	Stop

==== Shimadzu LCsolution Analysis Report ====

C:\newcgmp\purity\4O.lcd

Acquired by : Admin
Sample Name : 4O
Sample ID : 4O
Tray# : 1
Vail # : 25
Injection Volume : 5 uL
Data File Name : 4O.lcd
Method File Name : purity.lcm
Batch File Name : nancy compound purity.lcb
Report File Name : Default.lcr
Data Acquired : 25-11-2019 22:35:40
Data Processed : 25-11-2019 23:20:43

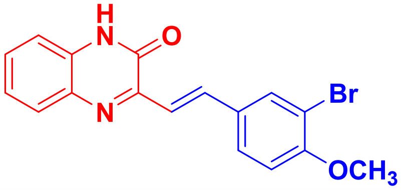

<Chromatogram>

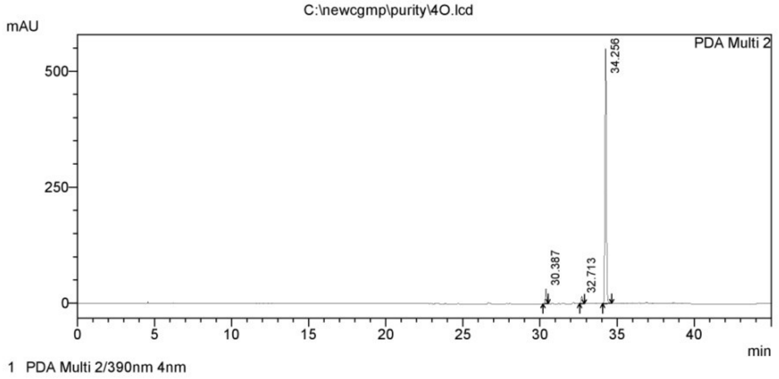

PeakTable

PDA Ch2 390nm 4nm

Peak#	Ret. Time	Area	Height	Area %	Height %
1	30.387	185753	32094	4.854	5.375
2	32.713	99969	15756	2.612	2.639
3	34.256	3541079	549207	92.534	91.986
Total		3826802	597057	100.000	100.000

HRMS Analysis of **4o**

LC HRMS- THERMOSCIENTIFIC- EXACTIVE
C18 COLUMN- Hypersil
MOBILE PHASE- methanol and water (0.1% formic acid)
Gradient method : 97% methanol and 3% water for 5 minutes.
Injected amount : 2Microlitre
Flow rate of solvent 150µl /minute
The source was operated in both positive and negative mode at an ion spray voltage of 3KV
Oven temperature was set to 30^{0}C

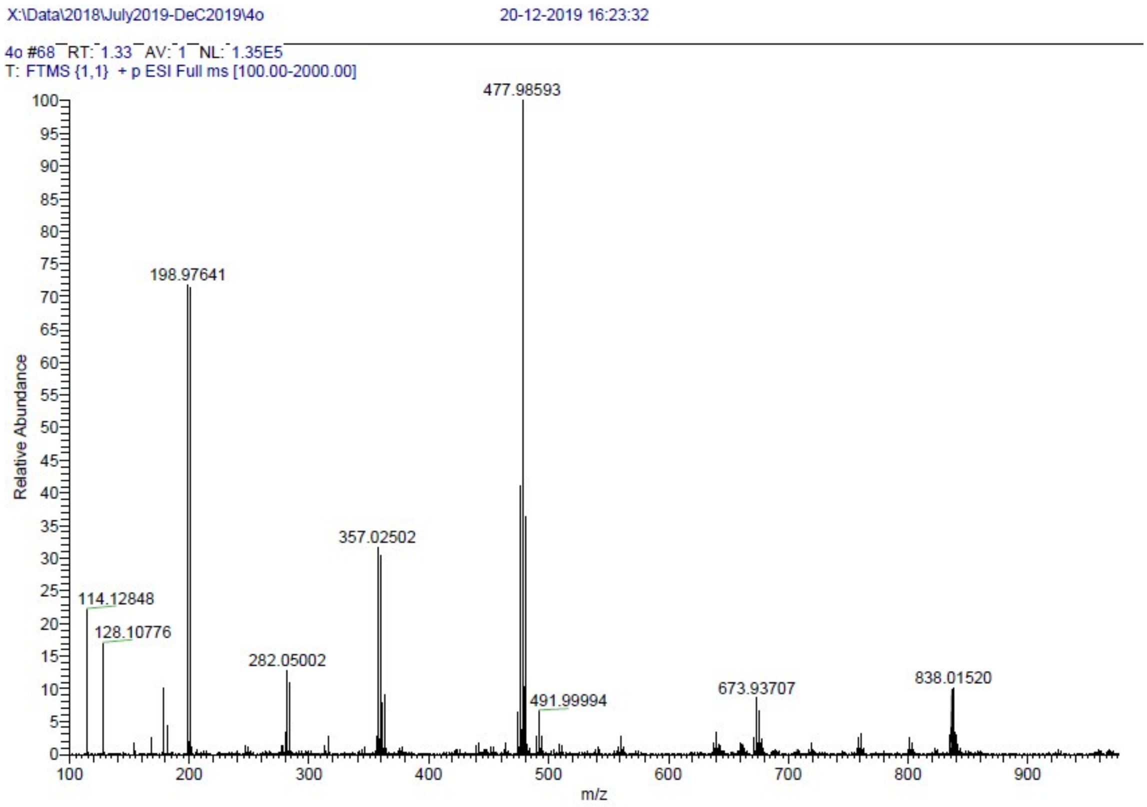

X:\Data\2018\July2019-DeC2019\4o 20-12-2019 16:23:32

4o #68 RT: 1.33 AV: 1 NL: 4.25E4
T: FTMS {1,1} + p ESI Full ms [100.00-2000.00]

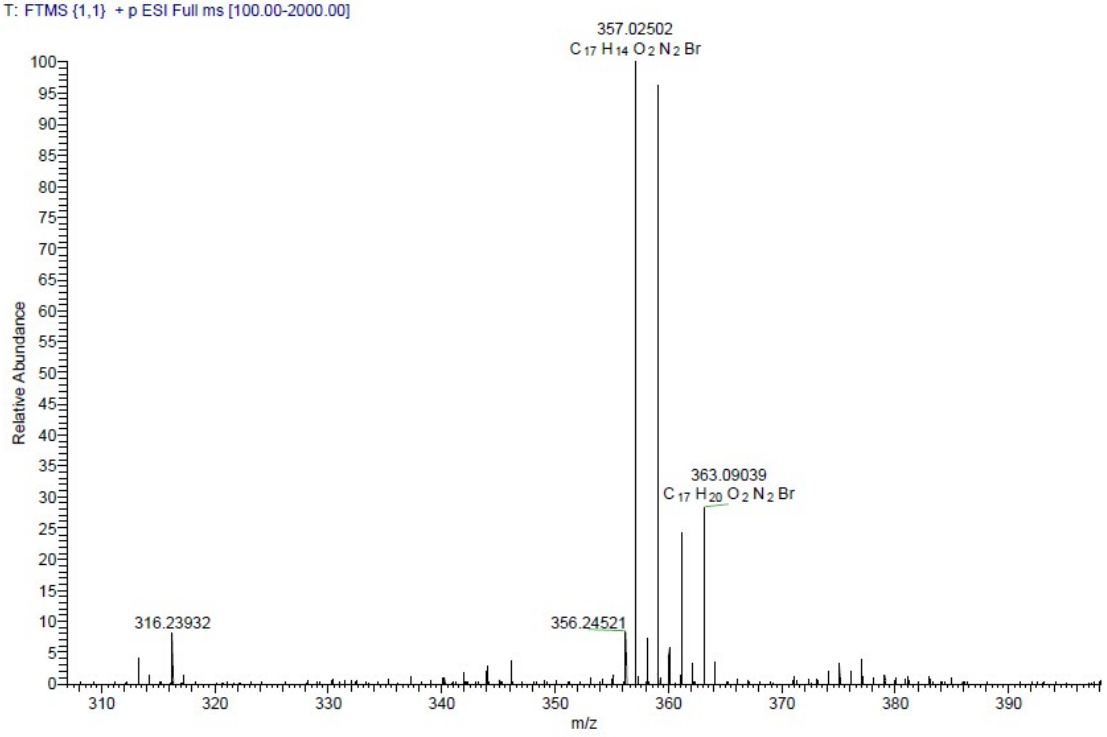

X:\Data\2018\July2019-DeC2019\4o 20-12-2019 16:23:32

4o #68 RT: 1.33 AV: 1 NL: 4.25E4
T: FTMS {1,1} + p ESI Full ms [100.00-2000.00]

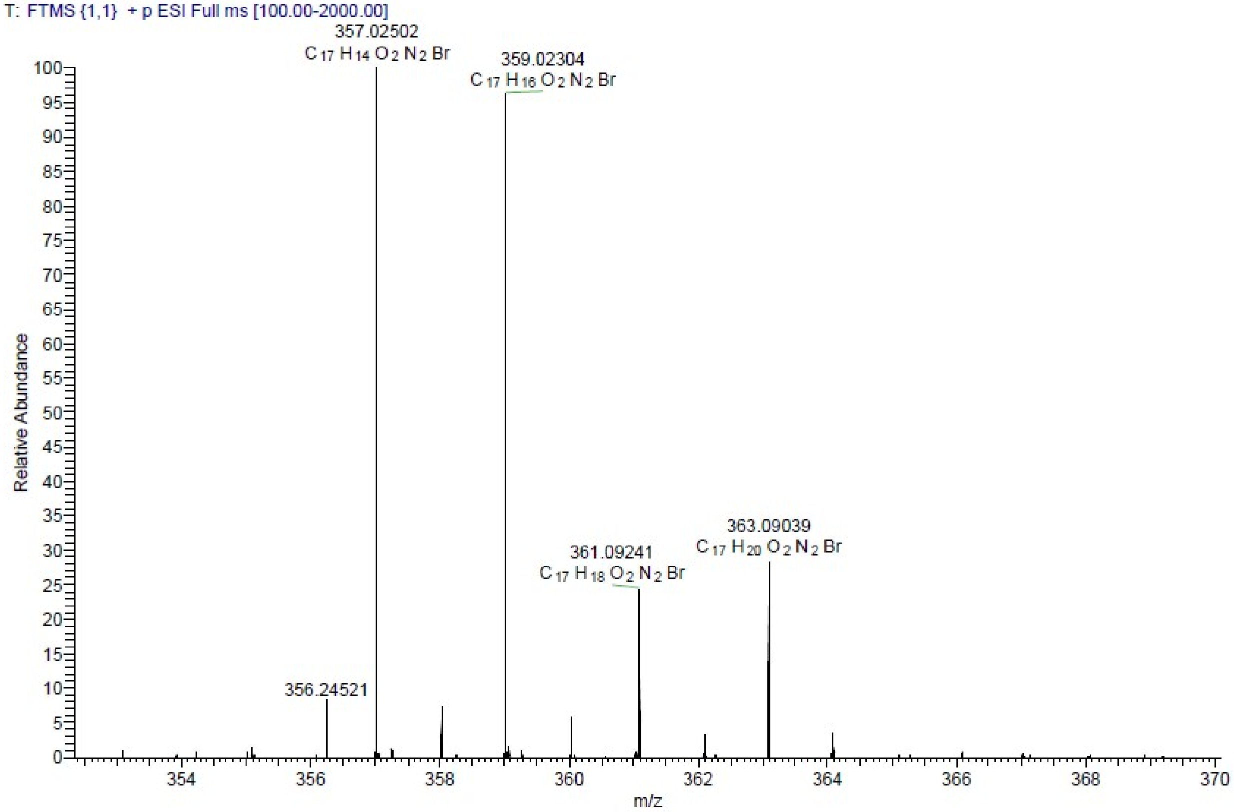

4p. *(E)-3-(2-bromo-4,5-dimethoxystyryl)quinoxalin-2(1H)-one*

^{1}H NMR spectrum (400 MHz, DMSO-d_6)

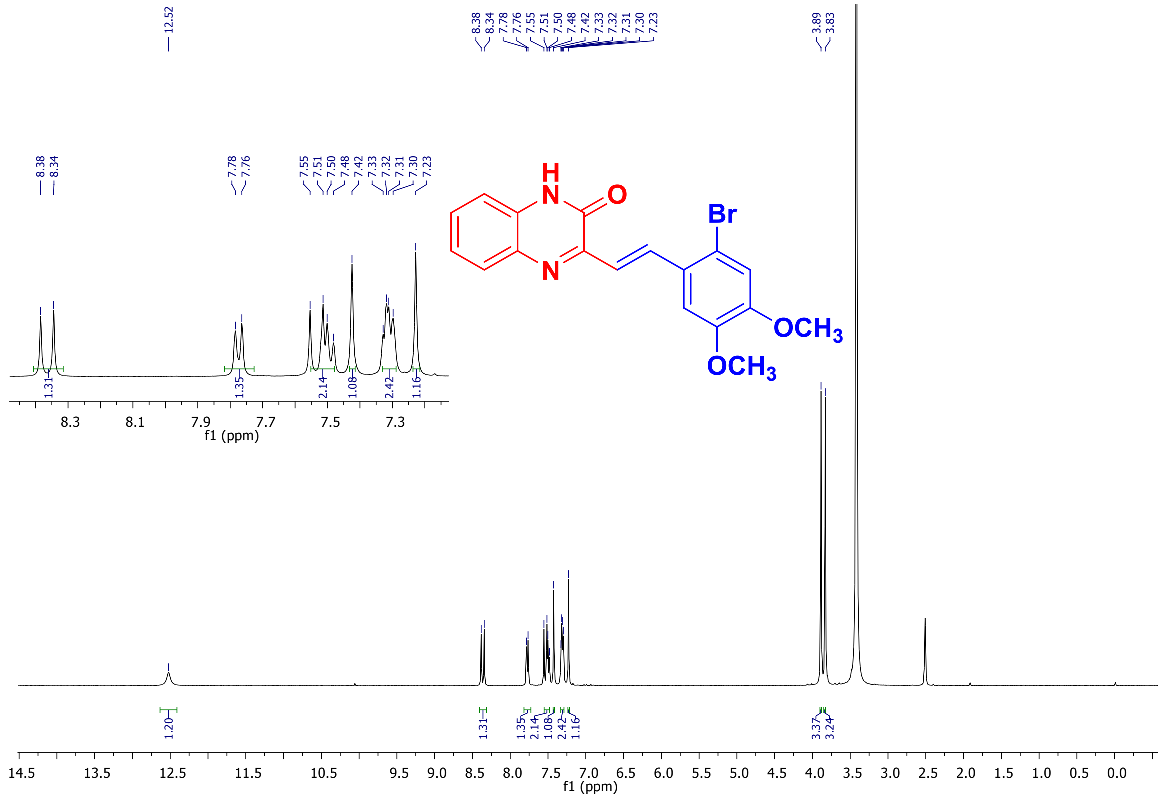

^{13}C NMR spectrum (101 MHz, DMSO-d_6)

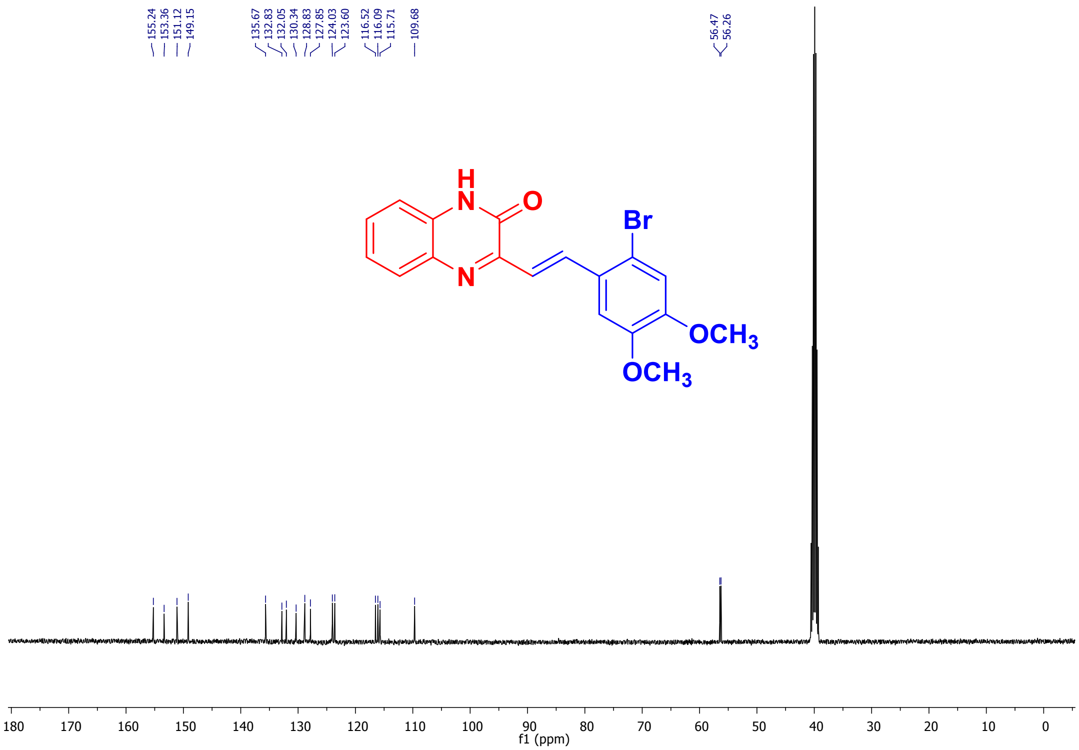

HPLC Analysis of **4p**

The HPLC purity was checked using Shimadzu HPLC system, consisting of purosphere C_{18} (5 µ, 250 × 4.6 mm) column and a PDA detector. The flow rate was 0.6 mL/min with the injection volume of 10 µL. The total run time was 45 min with gradient elution using 0.1% v/v formic acid in water (A) and mobile phase of acetonitrile (B). The gradient (WRT % v/v of A and B) was as shown in the **Table 2** :

Table 2 : Parameters used in HPLC purity check.

**Time (in min.)**	**WRT % v/v of B**	**WRT % v/v of A**
0	0	100
10	10	90
20	30	70
30	60	40
35	80	20
40	0	100
45	Stop	Stop

28-11-2019 17:13:23 1 / 1

==== Shimadzu LCsolution Analysis Report ====

C:\newcgmp\purity\4P.lcd

Acquired by : Admin
Sample Name : 4P
Sample ID : 4P
Tray# : 1
Vail # : 26
Injection Volume : 5 uL
Data File Name : 4P.lcd
Method File Name : purity.lcm
Batch File Name : nancy compound purity.lcb
Report File Name : Default.lcr
Data Acquired : 25-11-2019 23:21:11
Data Processed : 26-11-2019 10:27:39

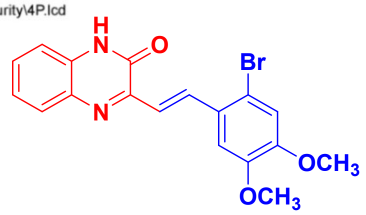

<Chromatogram>

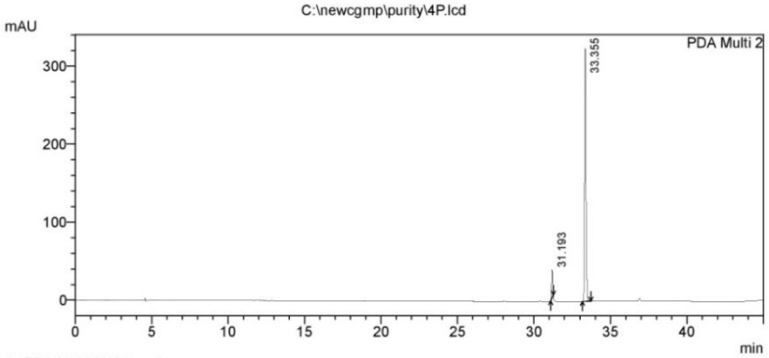

PeakTable

PDA Ch2 401nm 4nm

Peak#	Ret. Time	Area	Height	Area %	Height %
1	31.193	188291	35723	8.470	9.936
2	33.355	2034752	323807	91.530	90.064
Total		2223043	359530	100.000	100.000

HRMS Analysis of **4p**

LC HRMS- THERMOSCIENTIFIC- EXACTIVE
C18 COLUMN- Hypersil
MOBILE PHASE- methanol and water (0.1% formic acid)
Gradient method : 97% methanol and 3% water for 5 minutes.
Injected amount : 2Microlitre
Flow rate of solvent 150µl /minute
The source was operated in both positive and negative mode at an ion spray voltage of 3KV
Oven temperature was set to 30^{0}C

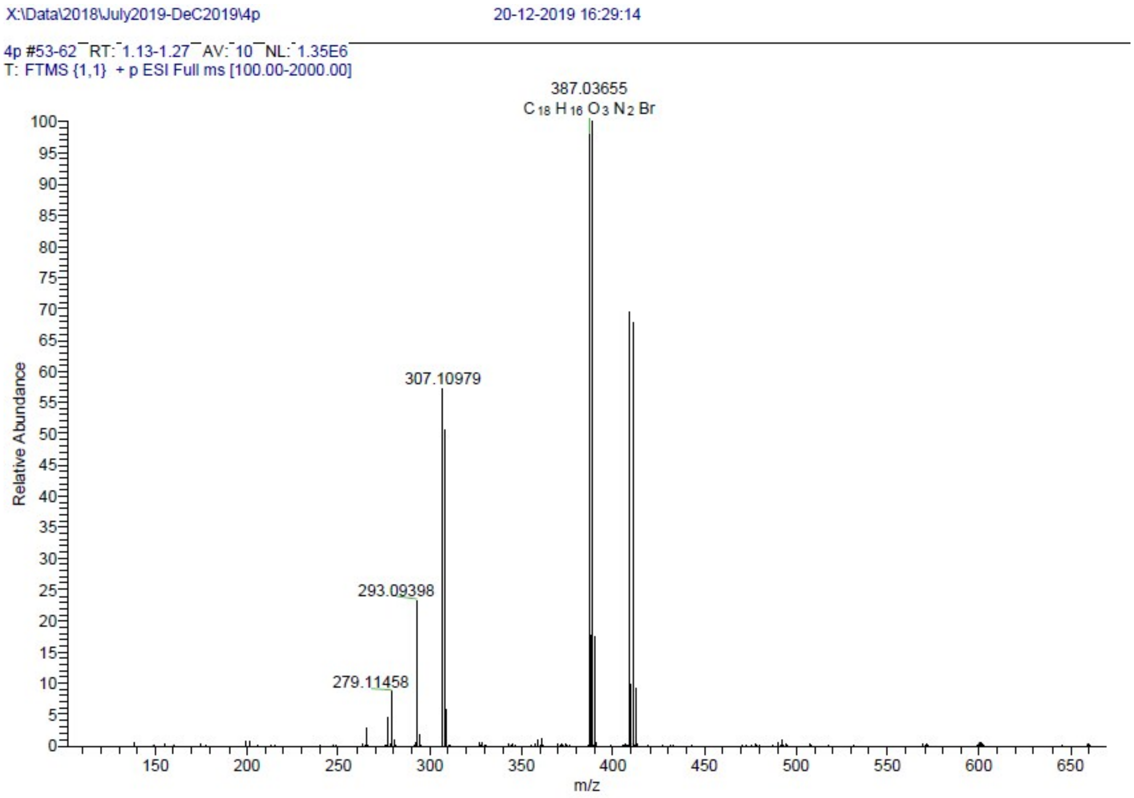

X:\Data\2018\July2019-DeC2019\4p 20-12-2019 16:29:14

4p #53-62 RT: 1.13-1.27 AV: 10 NL: 1.35E6
T: FTMS {1,1} + p ESI Full ms [100.00-2000.00]

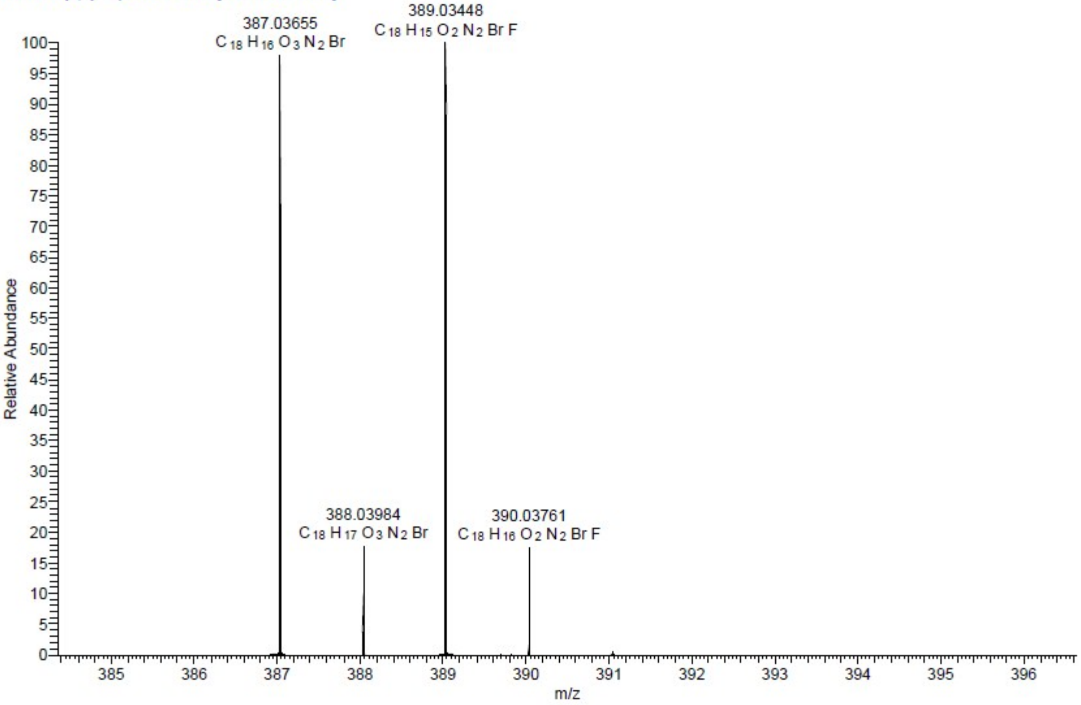

4q. ***(E)-3-(2-bromo-5-hydroxy-4-methoxystyryl)quinoxalin-2(1H)-one***

^{1}H NMR spectrum (400 MHz, DMSO-d$_6$)

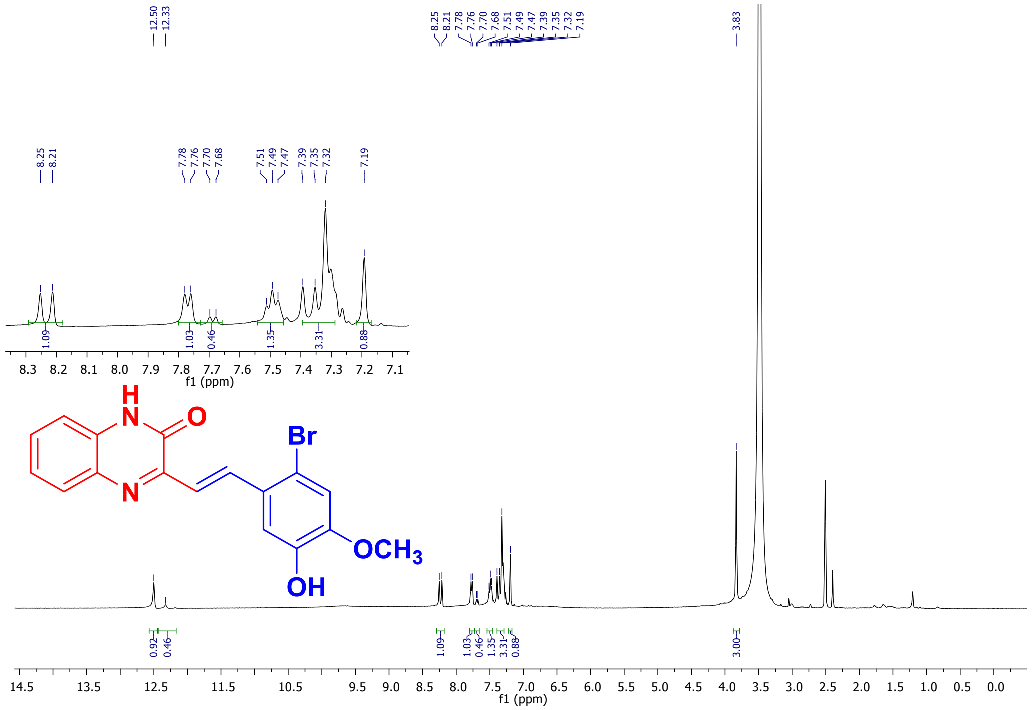

^{13}C NMR spectrum (101 MHz, DMSO-d$_6$)

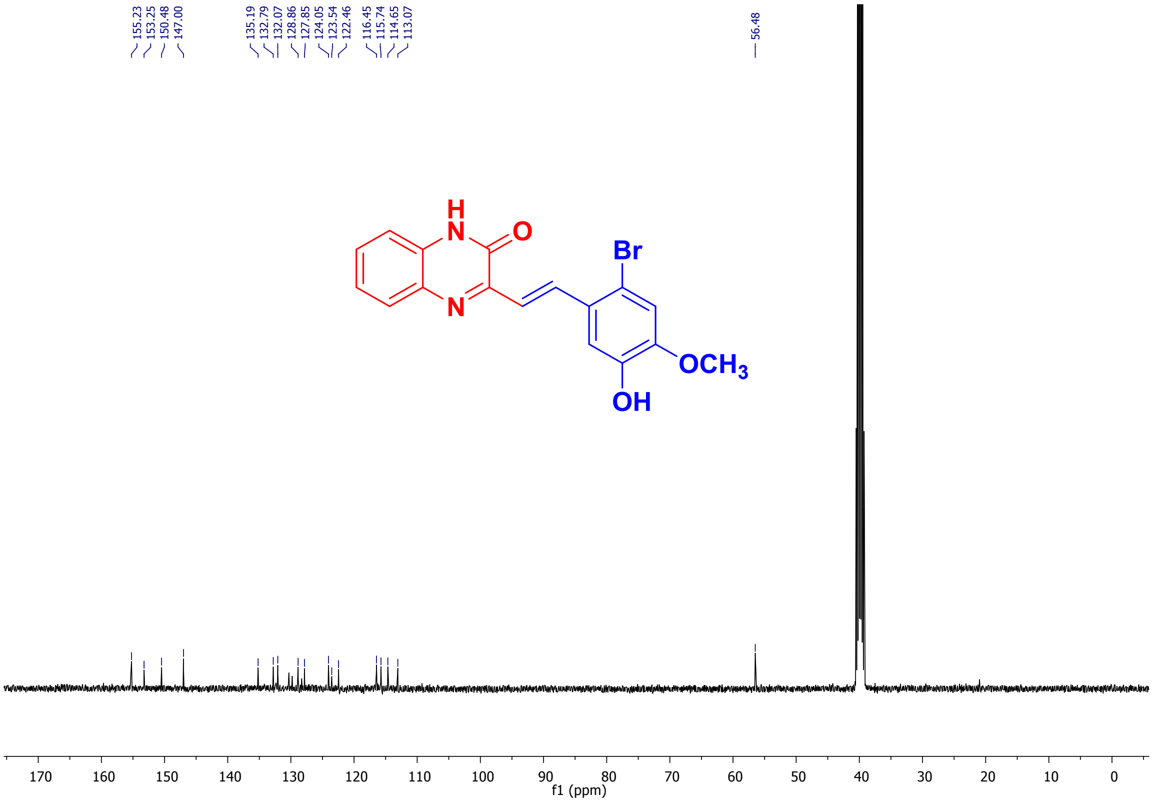

HPLC Analysis of **4q**

The HPLC purity was checked using Shimadzu HPLC system, consisting of purosphere C_{18} (5 µ, 250 × 4.6 mm) column and a PDA detector. The flow rate was 0.6 mL/min with the injection volume of 10 µL. The total run time was 45 min with gradient elution using 0.1% v/v formic acid in water (A) and mobile phase of acetonitrile (B). The gradient (WRT % v/v of A and B) was as shown in the **Table 2** :

Table 2 : Parameters used in HPLC purity check.

**Time (in min.)**	**WRT % v/v of B**	**WRT % v/v of A**
0	0	100
10	10	90
20	30	70
30	60	40
35	80	20
40	0	100
45	Stop	Stop

26-11-2019 10:24:16 1 / 1

==== Shimadzu LCsolution Analysis Report ====

C:\newcgmp\purity\4Q.lcd

Acquired by	: Admin
Sample Name	: 4Q
Sample ID	: 4Q
Tray#	: 1
Vail #	: 27
Injection Volume	: 5 uL
Data File Name	: 4Q.lcd
Method File Name	: purity.lcm
Batch File Name	: nancy compound purity.lcb
Report File Name	: Default.lcr
Data Acquired	: 26-11-2019 00:06:41
Data Processed	: 26-11-2019 00:51:43

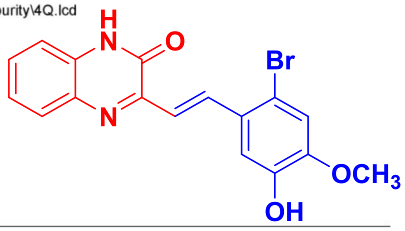

<Chromatogram>

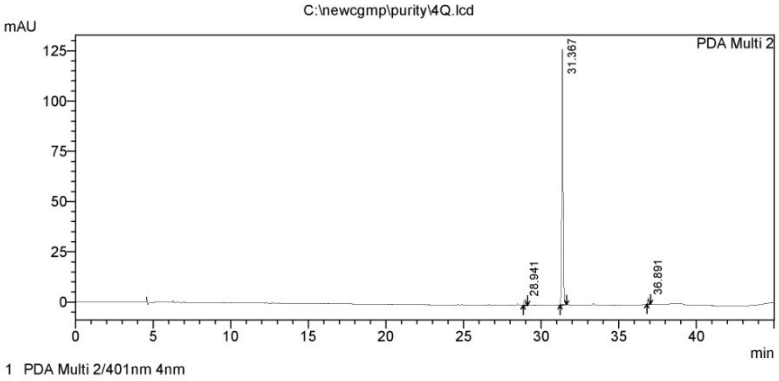

PeakTable

PDA Ch2 401nm 4nm

Peak#	Ret. Time	Area	Height	Area %	Height %
1	28.941	17003	2924	2.133	2.196
2	31.367	760286	127178	95.396	95.492
3	36.891	19687	3079	2.470	2.312
Total		796976	133182	100.000	100.000

HRMS Analysis of **4q**

LC HRMS- THERMOSCIENTIFIC- EXACTIVE
C18 COLUMN- Hypersil
MOBILE PHASE- methanol and water (0.1% formic acid)
Gradient method : 97% methanol and 3% water for 5 minutes.
Injected amount : 2Microlitre
Flow rate of solvent 150µl /minute
The source was operated in both positive and negative mode at an ion spray voltage of 3KV
Oven temperature was set to 30^{0}C

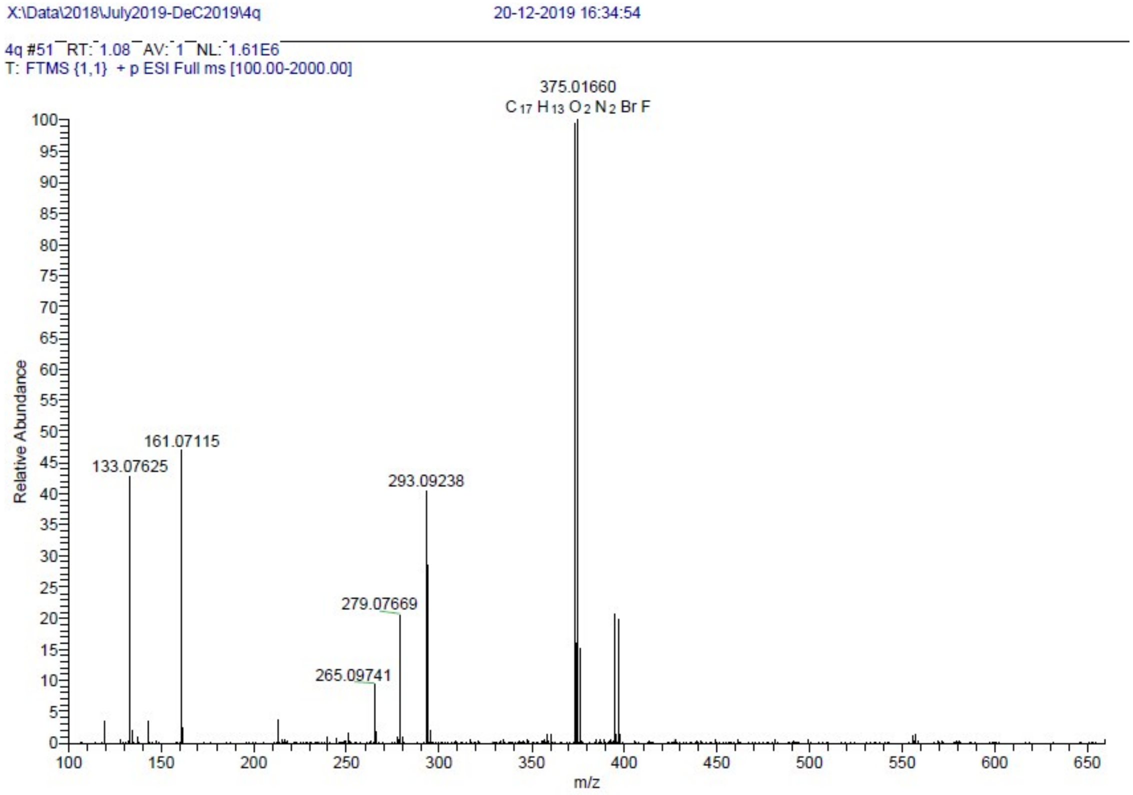

X:\Data\2018\July2019-DeC2019\4q 20-12-2019 16:34:54

4q #51 RT: 1.08 AV: 1 NL: 1.61E6
T: FTMS {1,1} + p ESI Full ms [100.00-2000.00]

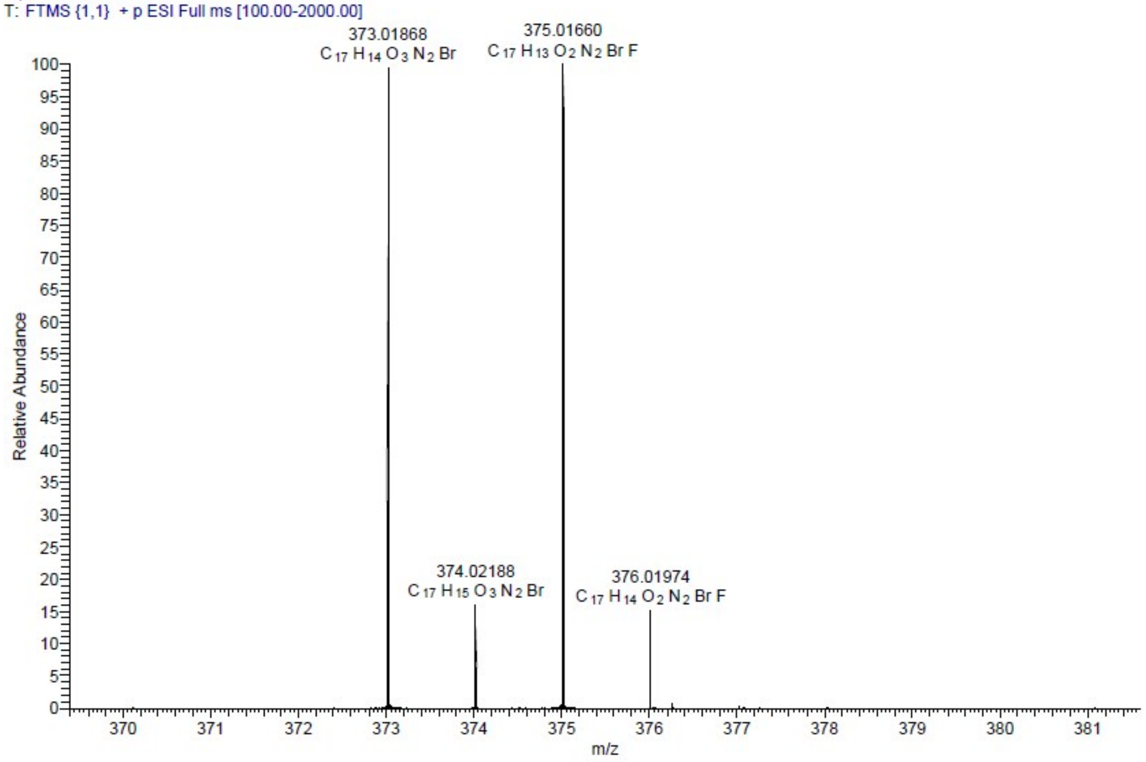

4r. ***(E)-3-(2-(trifluoromethyl)styryl)quinoxalin-2(1H)-one***

^{1}H NMR spectrum (400 MHz, DMSO-d_6)

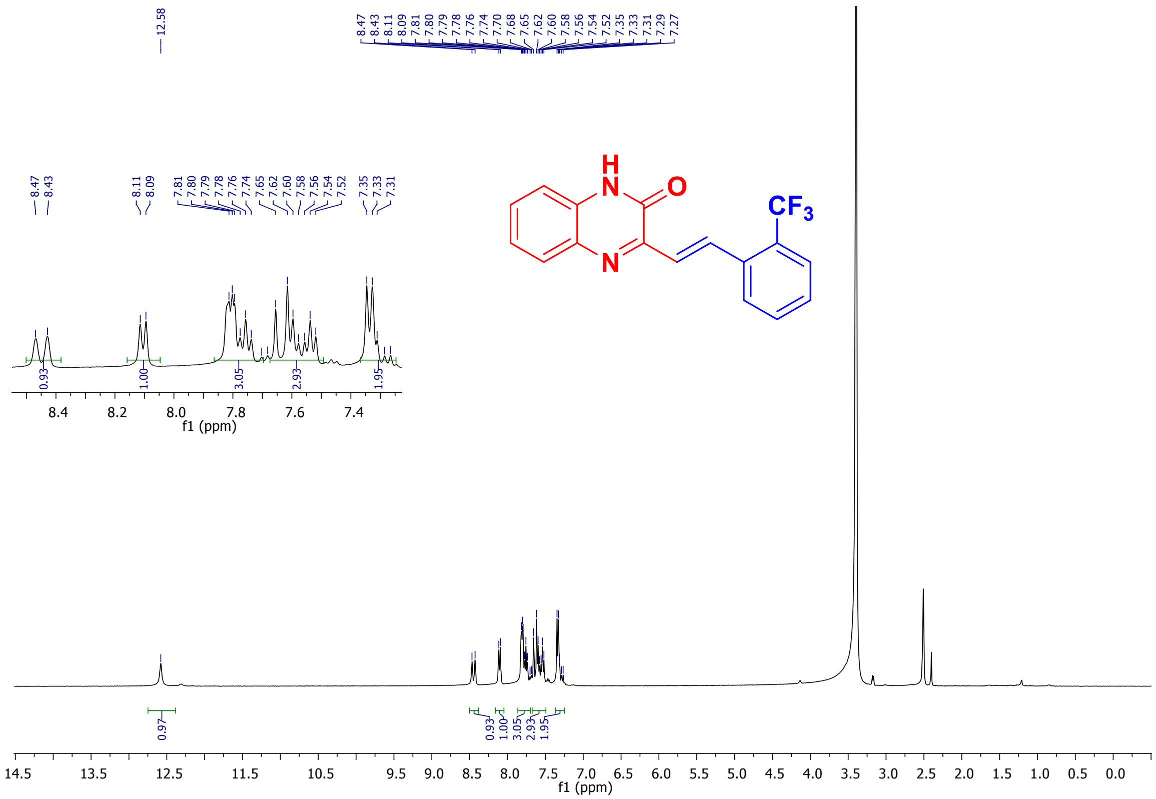

^{13}C NMR spectrum (101 MHz, DMSO-d_6)

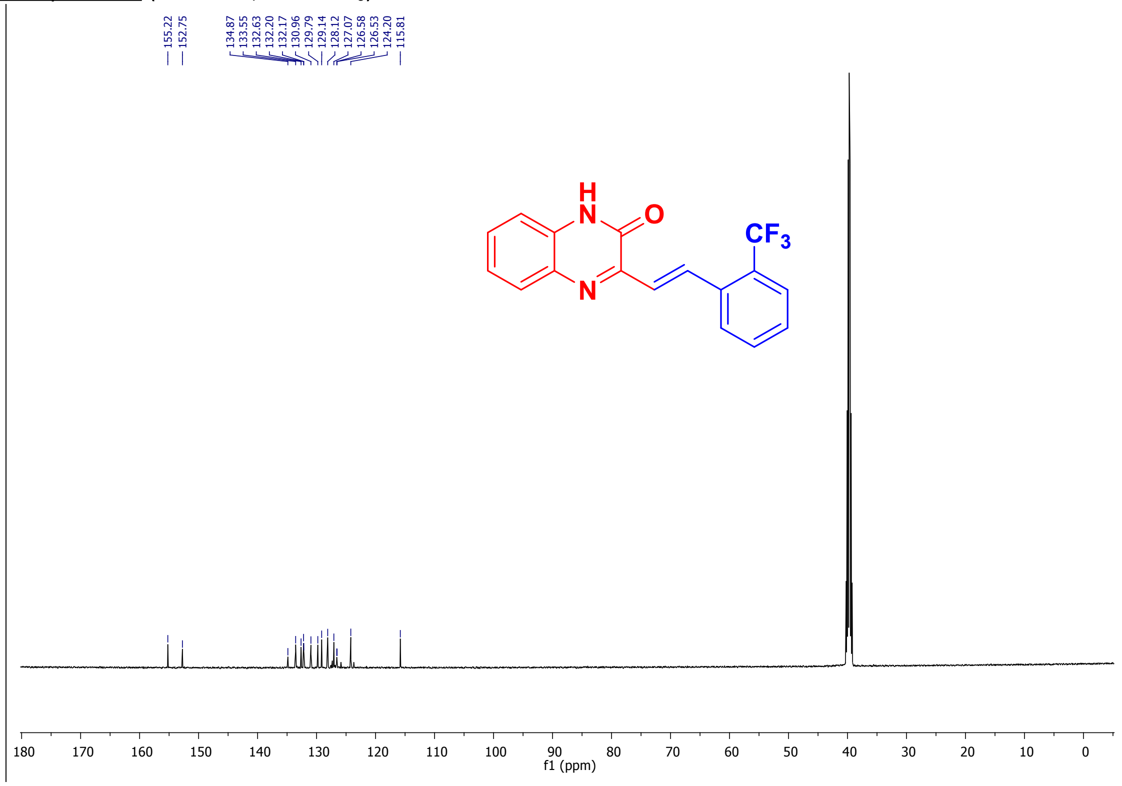

^{19}F NMR spectrum (376 MHz, DMSO-d_6)

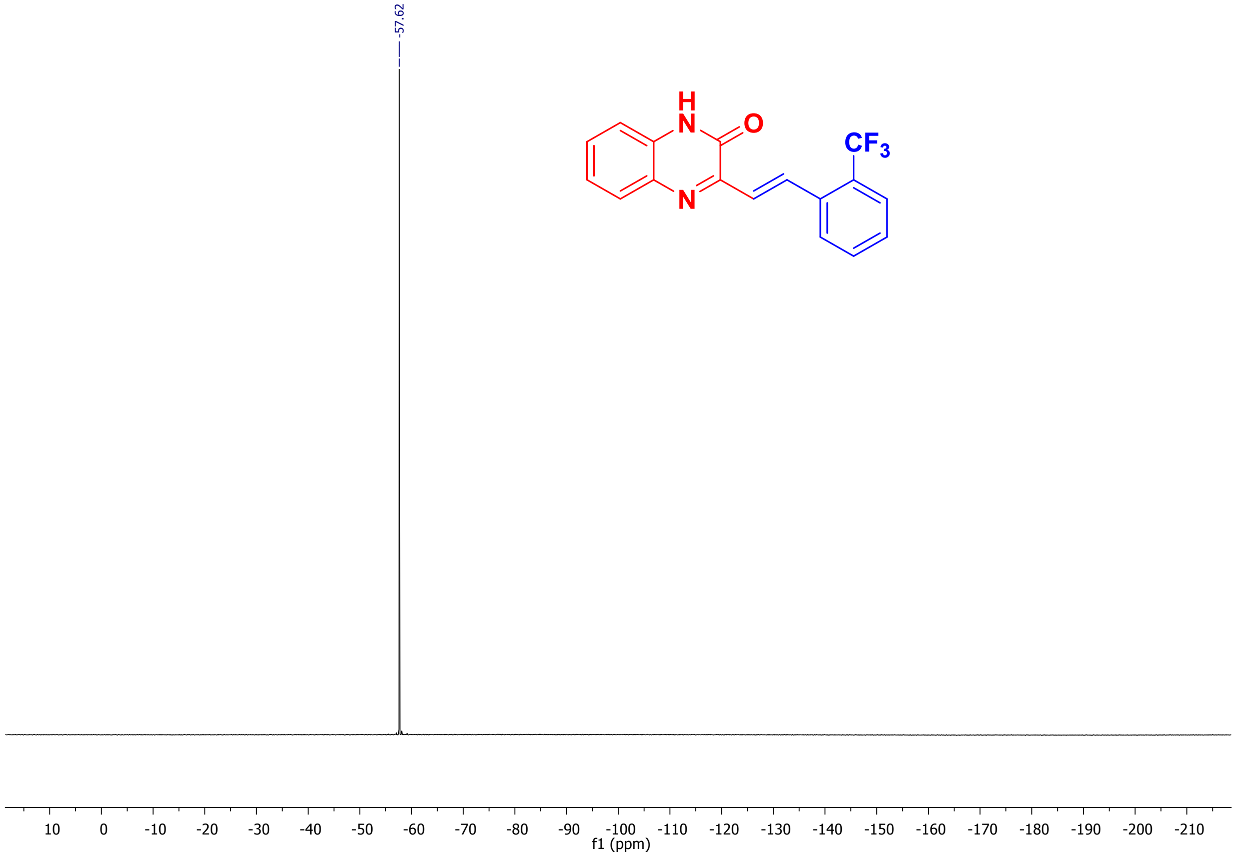

HPLC Analysis of **4r**

The HPLC purity was checked using Shimadzu HPLC system, consisting of purosphere C_{18} (5 µ, 250 × 4.6 mm) column and a PDA detector. The flow rate was 0.6 mL/min with the injection volume of 10 µL. The total run time was 45 min with gradient elution using 0.1% v/v formic acid in water (A) and mobile phase of acetonitrile (B). The gradient (WRT % v/v of A and B) was as shown in the **Table 2** :

Table 2 : Parameters used in HPLC purity check.

**Time (in min.)**	**WRT % v/v of B**	**WRT % v/v of A**
0	0	100
10	10	90
20	30	70
30	60	40
35	80	20
40	0	100
45	Stop	Stop

26-11-2019 10:19:41 1 / 1

==== Shimadzu LCsolution Analysis Report ====

C:\newcgmp\purity\4R.lcd

Acquired by : Admin
Sample Name : 4R
Sample ID : 4R
Tray# : 1
Vail # : 28
Injection Volume : 10 uL
Data File Name : 4R.lcd
Method File Name : purity.lcm
Batch File Name : nancy compound purity.lcb
Report File Name : Default.lcr
Data Acquired : 26-11-2019 00:52:14
Data Processed : 26-11-2019 01:37:16

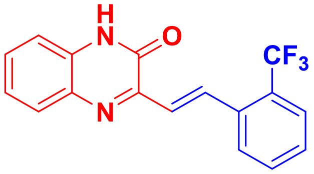

<Chromatogram>

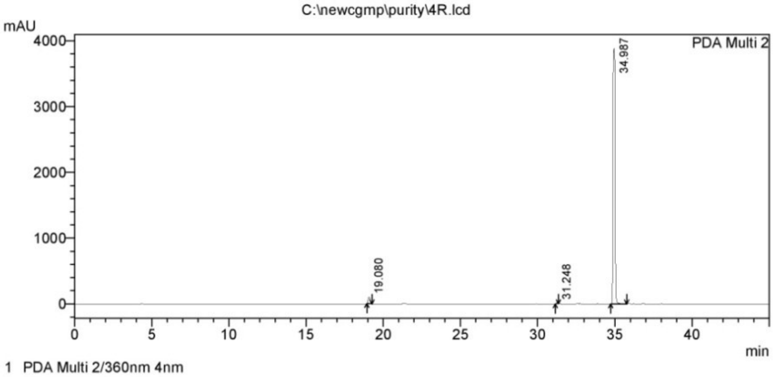

1 PDA Multi 2/360nm 4nm

PeakTable

PDA Ch2 360nm 4nm

Peak#	Ret. Time	Area	Height	Area %	Height %
1	19.080	841793	103453	2.309	2.584
2	31.248	102596	19554	0.281	0.488
3	34.987	35516422	3880803	97.410	96.928
Total		36460811	4003809	100.000	100.000

HRMS Analysis of **4r**

LC HRMS- THERMOSCIENTIFIC- EXACTIVE
C18 COLUMN- Hypersil
MOBILE PHASE- methanol and water (0.1% formic acid)
Gradient method : 97% methanol and 3% water for 5 minutes.
Injected amount : 2Microlitre
Flow rate of solvent 150µl /minute
The source was operated in both positive and negative mode at an ion spray voltage of 3KV
Oven temperature was set to 30^{0}C

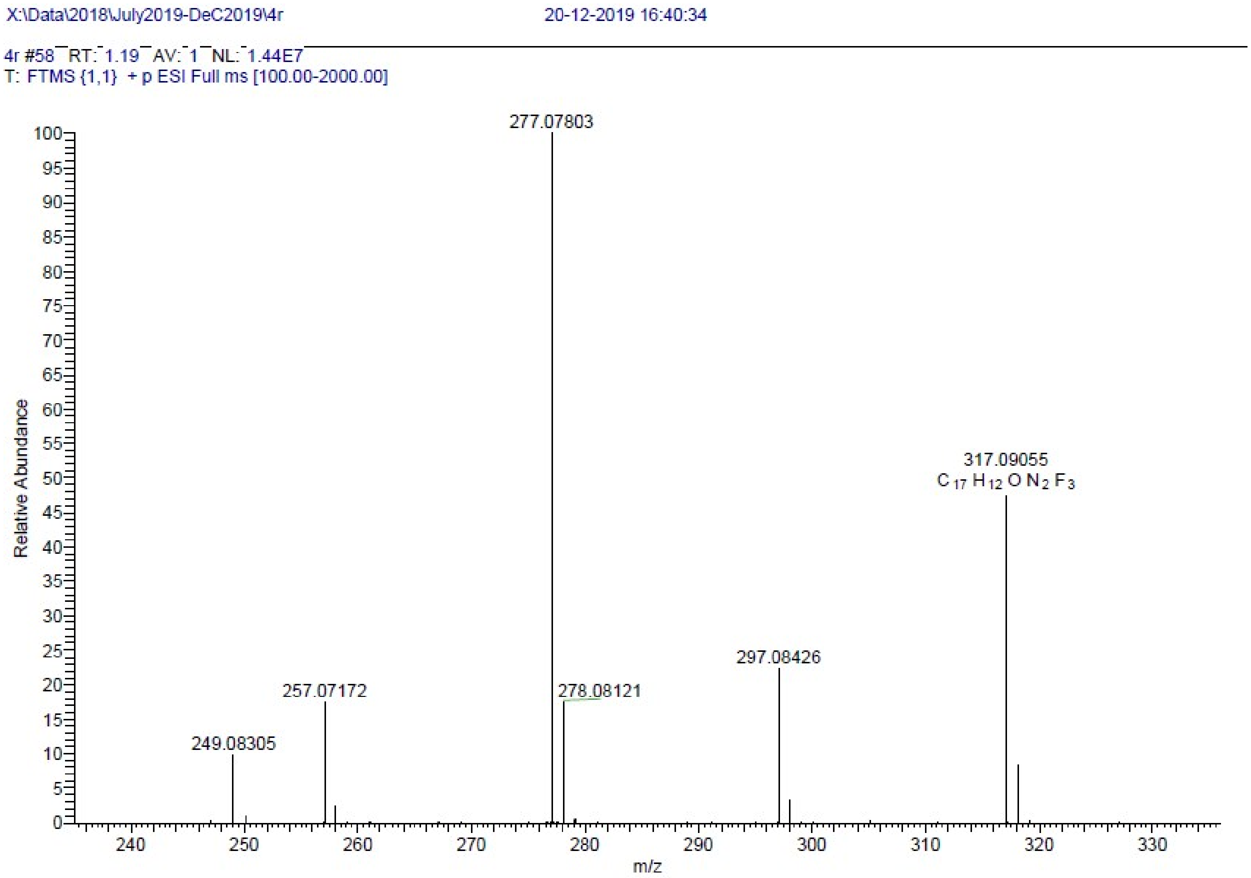

4s. ***(E)-3-(4-(methylthio)styryl)quinoxalin-2(1H)-one***

<u>^{1}H NMR spectrum</u> (400 MHz, DMSO-d$_6$)

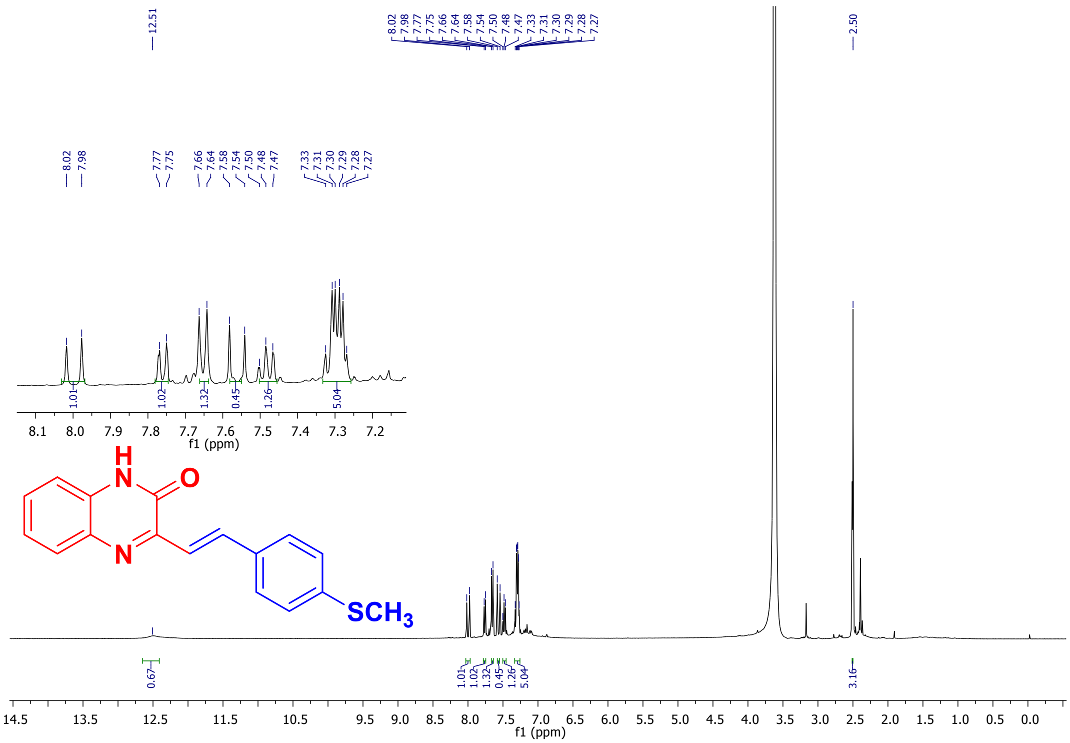

<u>^{13}C NMR spectrum</u> (101 MHz, DMSO-d$_6$)

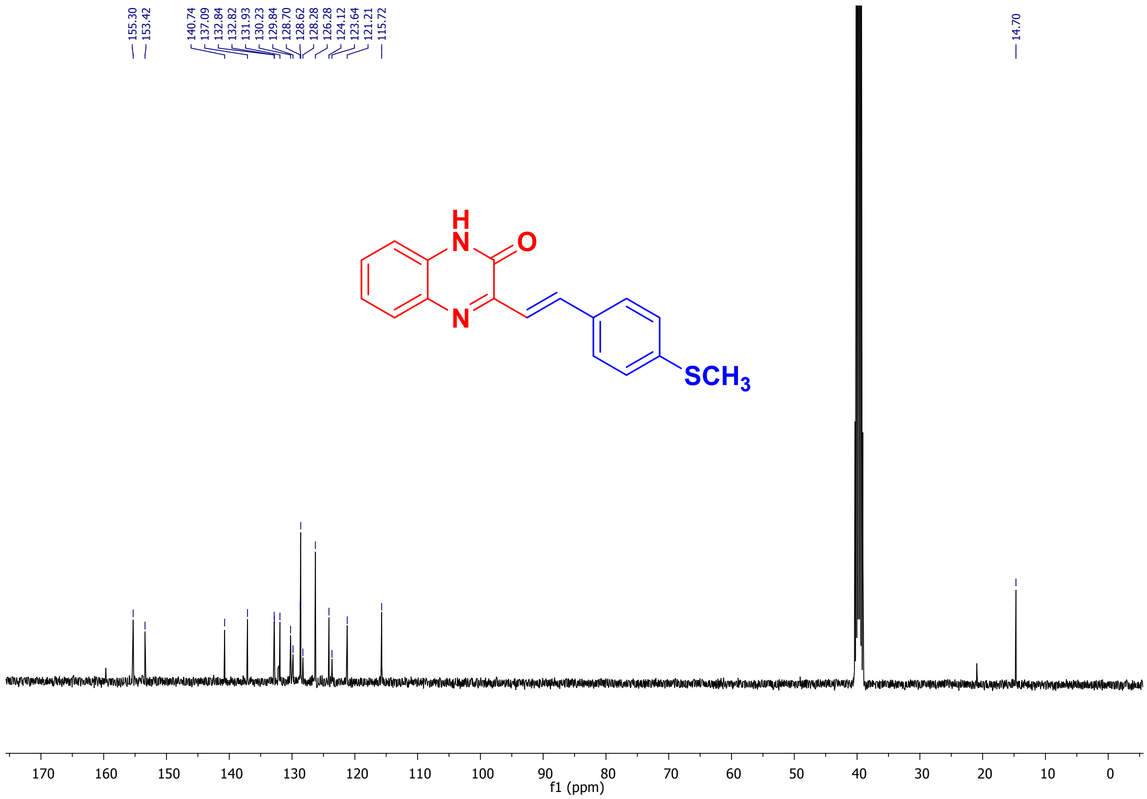

HPLC Analysis of **4s**

The HPLC purity was checked using Shimadzu HPLC system, consisting of purosphere C_{18} (5 µ, 250 × 4.6 mm) column and a PDA detector. The flow rate was 0.6 mL/min with the injection volume of 10 µL. The total run time was 45 min with gradient elution using 0.1% v/v formic acid in water (A) and mobile phase of acetonitrile (B). The gradient (WRT % v/v of A and B) was as shown in the **Table 2** :

Table 2 : Parameters used in HPLC purity check.

**Time (in min.)**	**WRT % v/v of B**	**WRT % v/v of A**
0	0	100
10	10	90
20	30	70
30	60	40
35	80	20
40	0	100
45	Stop	Stop

==== Shimadzu LCsolution Analysis Report ====

C:\4S purity2.lcd

Acquired by	: Admin
Sample Name	: 4s
Sample ID	: 4S
Tray#	: 1
Vail #	: 40
Injection Volume	: 10 uL
Data File Name	: 4S purity2.lcd
Method File Name	: purity.lcm
Batch File Name	:
Report File Name	: Default.lcr
Data Acquired	: 04-02-2020 15:33:28
Data Processed	: 04-02-2020 16:18:31

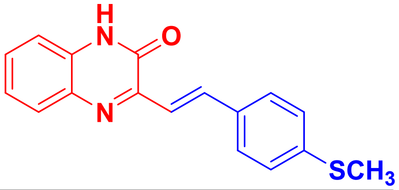

<Chromatogram>

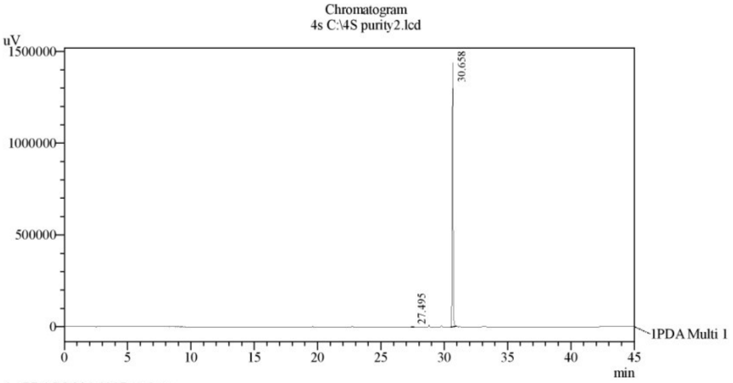

1 PDA Multi 1 / 397nm 4nm

PeakTable

PDA Ch1 397nm 4nm

Peak#	Ret. Time	Area	Height	Area %	Height %
1	27.495	2712	651	0.039	0.045
2	30.658	7015045	1437867	99.961	99.955
Total		7017757	1438518	100.000	100.000

HRMS Analysis of **4s**

LC HRMS- THERMOSCIENTIFIC- EXACTIVE
C18 COLUMN- Hypersil
MOBILE PHASE- methanol and water (0.1% formic acid)
Gradient method : 97% methanol and 3% water for 5 minutes.
Injected amount : 2Microlitre
Flow rate of solvent 150µl /minute
The source was operated in both positive and negative mode at an ion spray voltage of 3KV
Oven temperature was set to 30^{0}C

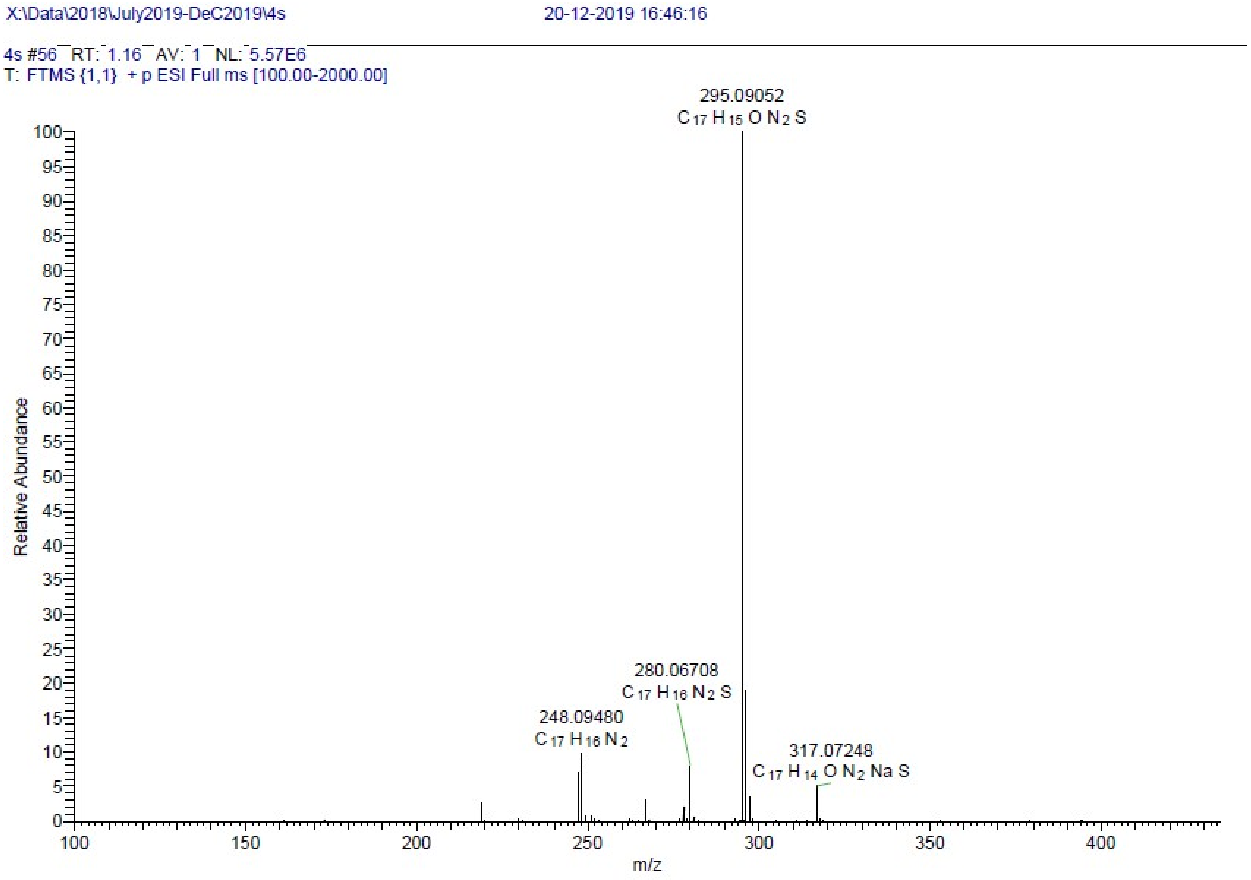

4t. ***(E)-3-(2-(pyridin-2-yl)vinyl)quinoxalin-2(1H)-one***

^{1}H NMR spectrum (400 MHz, DMSO-d$_6$)

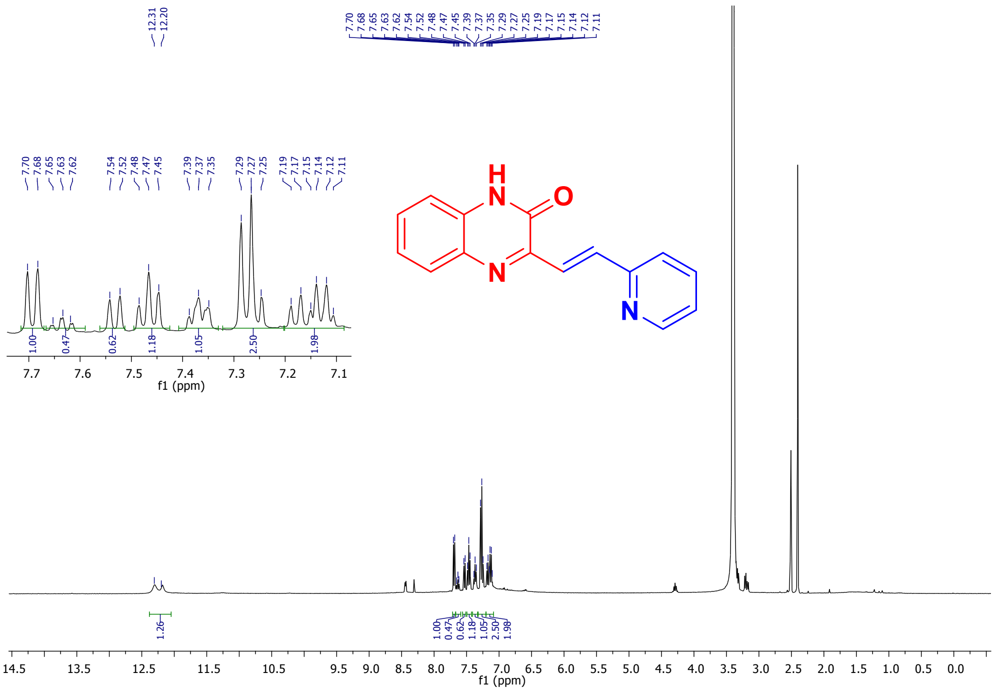

^{13}C NMR spectrum (101 MHz, DMSO-d$_6$)

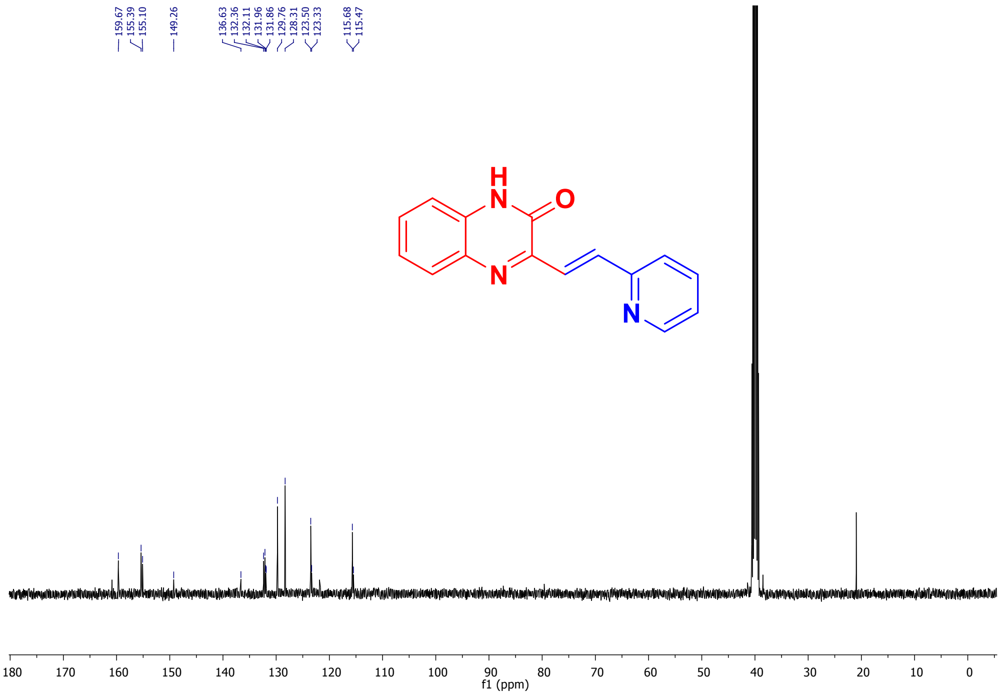

4u. ***(E)-3-(2-(thiophen-2-yl)vinyl)quinoxalin-2(1H)-one***

^{1}H NMR spectrum (400 MHz, DMSO-d_6)

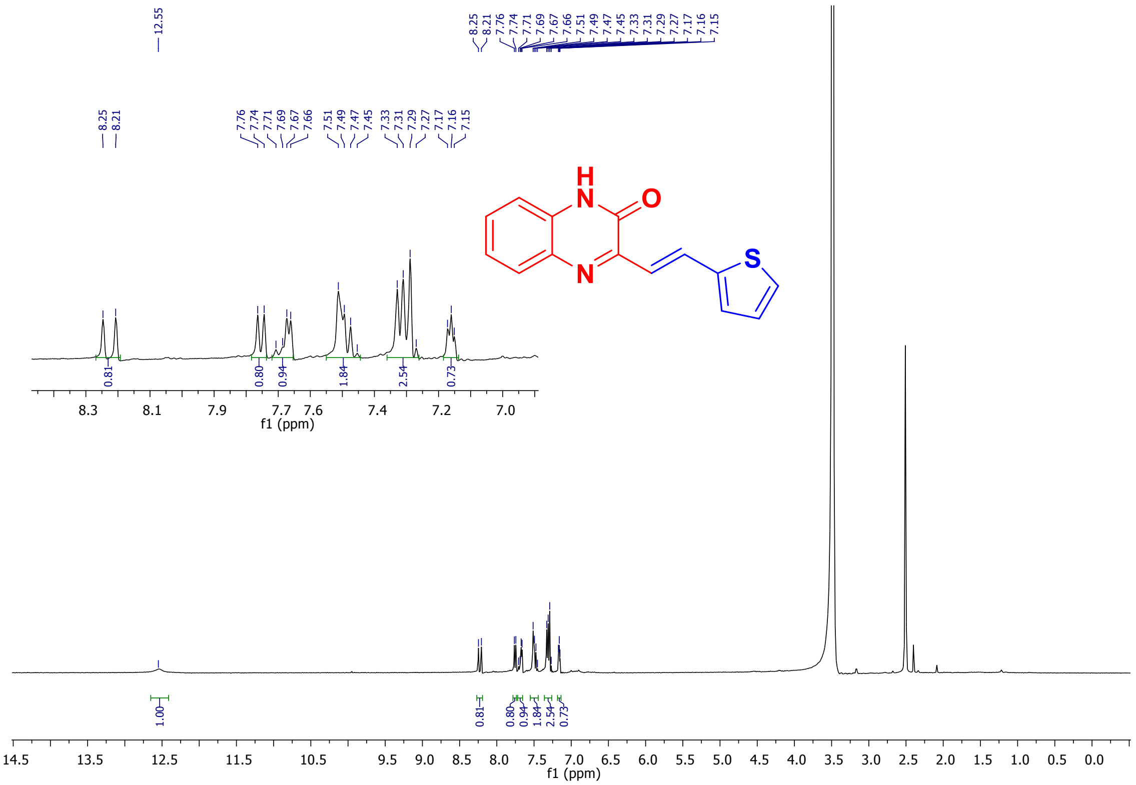

^{13}C NMR spectrum (101 MHz, DMSO-d_6)

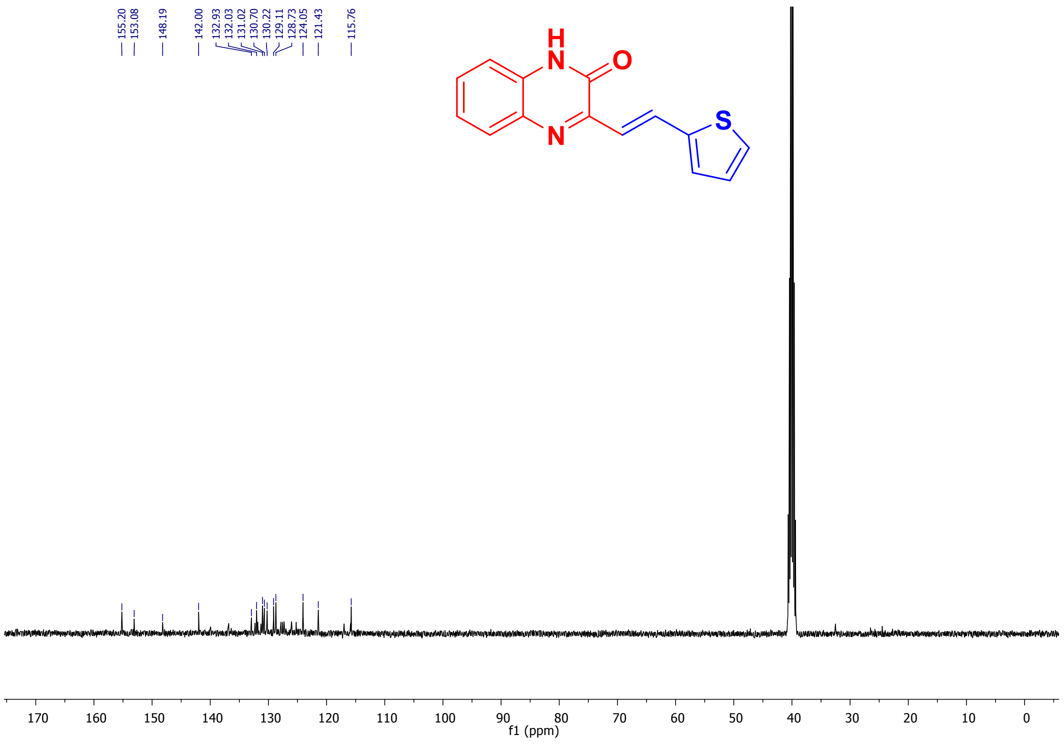

4v. ***(E)-3-(2-(benzo[d][1,3]dioxol-5-yl)vinyl)quinoxalin-2(1H)-one***

^{1}H NMR spectrum (400 MHz, DMSO-d$_6$)

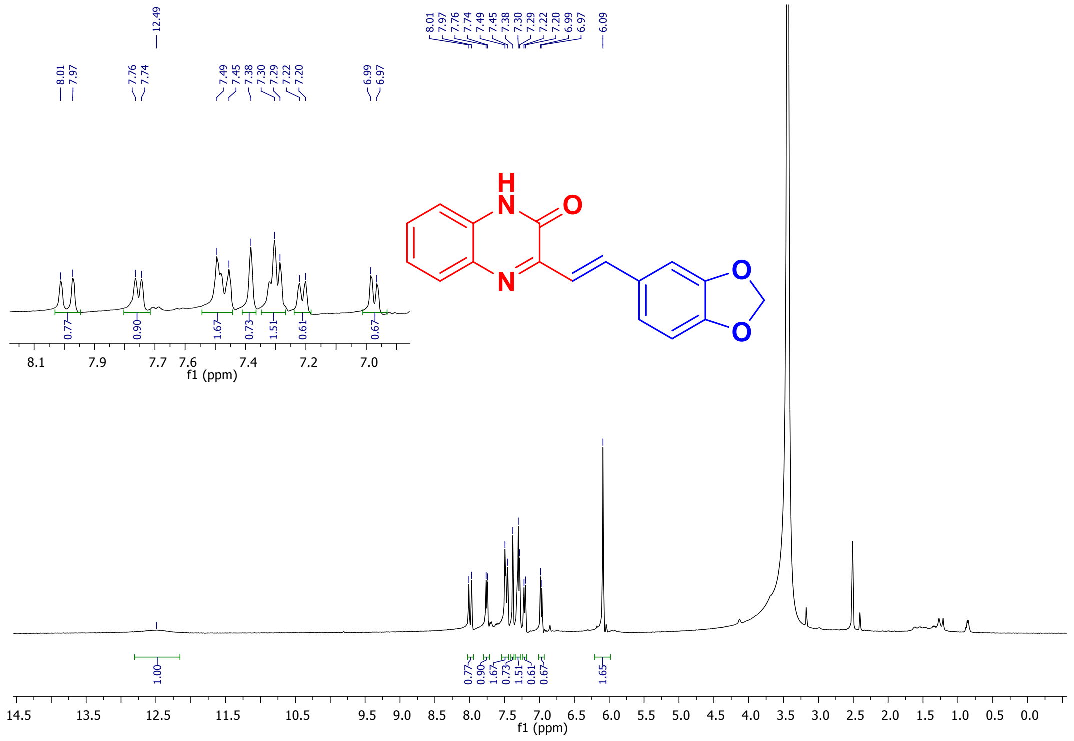

^{13}C NMR spectrum (101 MHz, DMSO-d$_6$)

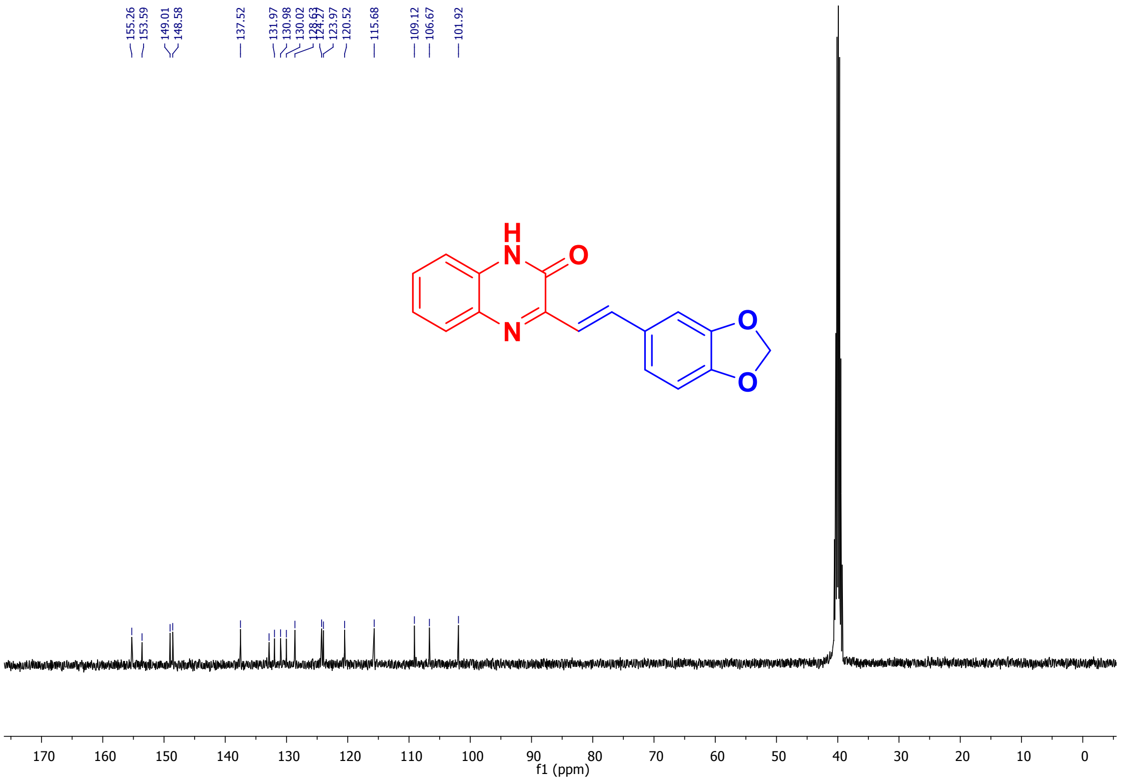

HPLC Analysis of **4v**

The HPLC purity was checked using Shimadzu HPLC system, consisting of purosphere C_{18} (5 µ, 250 × 4.6 mm) column and a PDA detector. The flow rate was 0.6 mL/min with the injection volume of 10 µL. The total run time was 45 min with gradient elution using 0.1% v/v formic acid in water (A) and mobile phase of acetonitrile (B). The gradient (WRT % v/v of A and B) was as shown in the **Table 2** :

Table 2 : Parameters used in HPLC purity check.

Time (in min.)	WRT % v/v of B	WRT % v/v of A
0	0	100
10	10	90
20	30	70
30	60	40
35	80	20
40	0	100
45	Stop	Stop

==== Shimadzu LCsolution Analysis Report ====

C:\newcgmp\purity\4V.lcd

Acquired by : Admin
Sample Name : 4V
Sample ID : 4V
Tray# : 1
Vail # : 30
Injection Volume : 5 uL
Data File Name : 4V.lcd
Method File Name : purity.lcm
Batch File Name : nancy compound purity.lcb
Report File Name : Default.lcr
Data Acquired : 26-11-2019 02:23:15
Data Processed : 26-11-2019 10:06:52

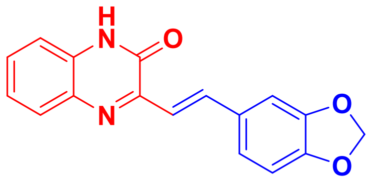

<Chromatogram>

C:\newcgmp\purity\4V.lcd

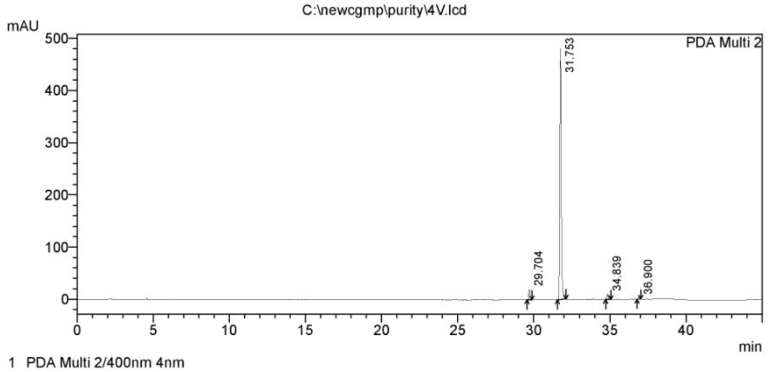

1 PDA Multi 2/400nm 4nm

PeakTable

PDA Ch2 400nm 4nm

Peak#	Ret. Time	Area	Height	Area %	Height %
1	29.704	128838	20492	3.636	3.972
2	31.753	3322481	481271	93.777	93.294
3	34.839	70407	10686	1.987	2.071
4	36.900	21235	3416	0.599	0.662
Total		3542961	515865	100.000	100.000

HRMS Analysis of **4v**

LC HRMS- THERMOSCIENTIFIC- EXACTIVE
C18 COLUMN- Hypersil
MOBILE PHASE- methanol and water (0.1% formic acid)
Gradient method : 97% methanol and 3% water for 5 minutes.
Injected amount : 2Microlitre
Flow rate of solvent 150µl /minute
The source was operated in both positive and negative mode at an ion spray voltage of 3KV
Oven temperature was set to 30^{0}C

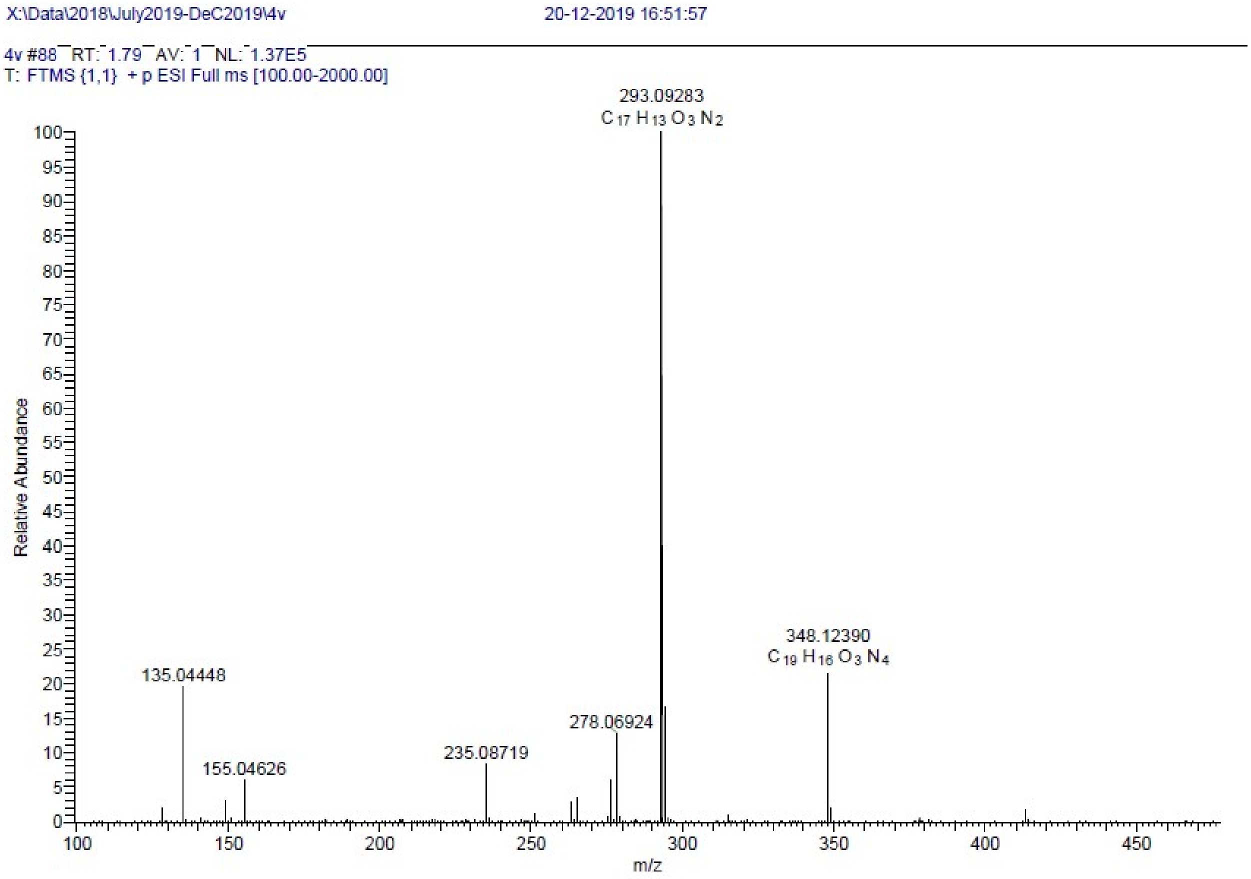

4w. ***(E)-3-(2-(4-bromobenzo[d][1,3]dioxol-5-yl)vinyl)quinoxalin-2(1H)-one***

^{1}H NMR spectrum (400 MHz, DMSO-d_6)

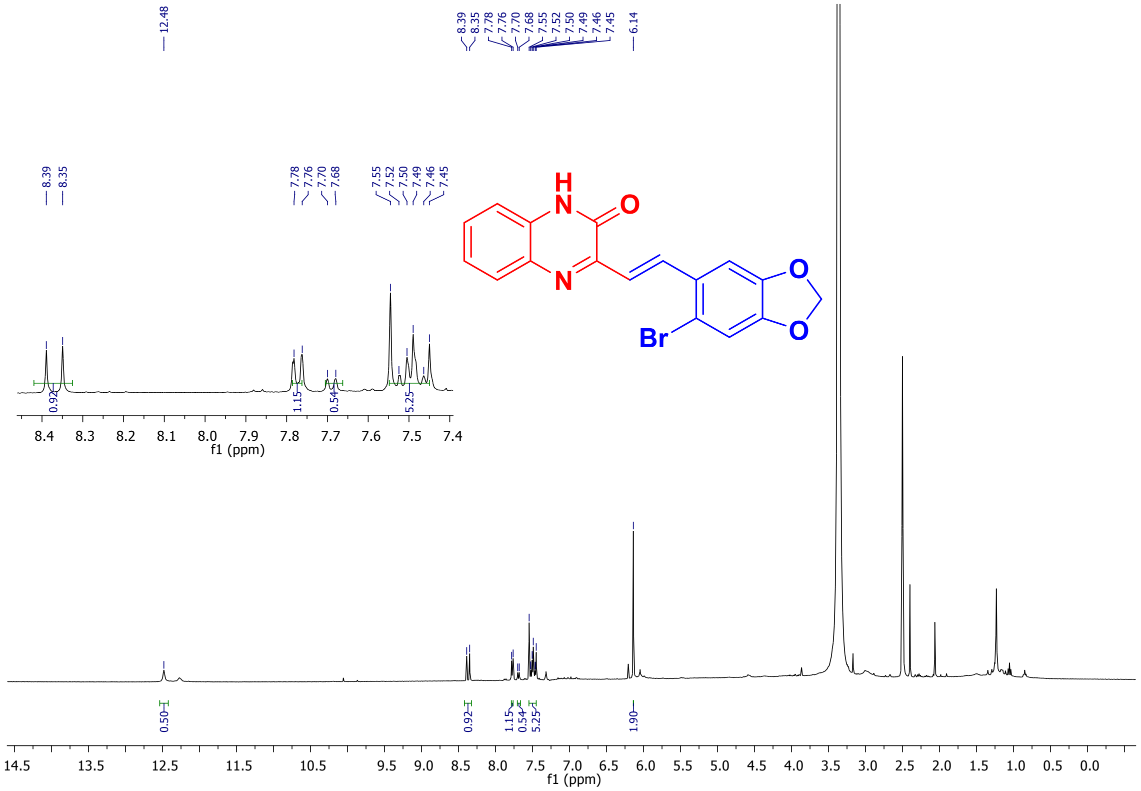

^{13}C NMR spectrum (101 MHz, DMSO-d_6)

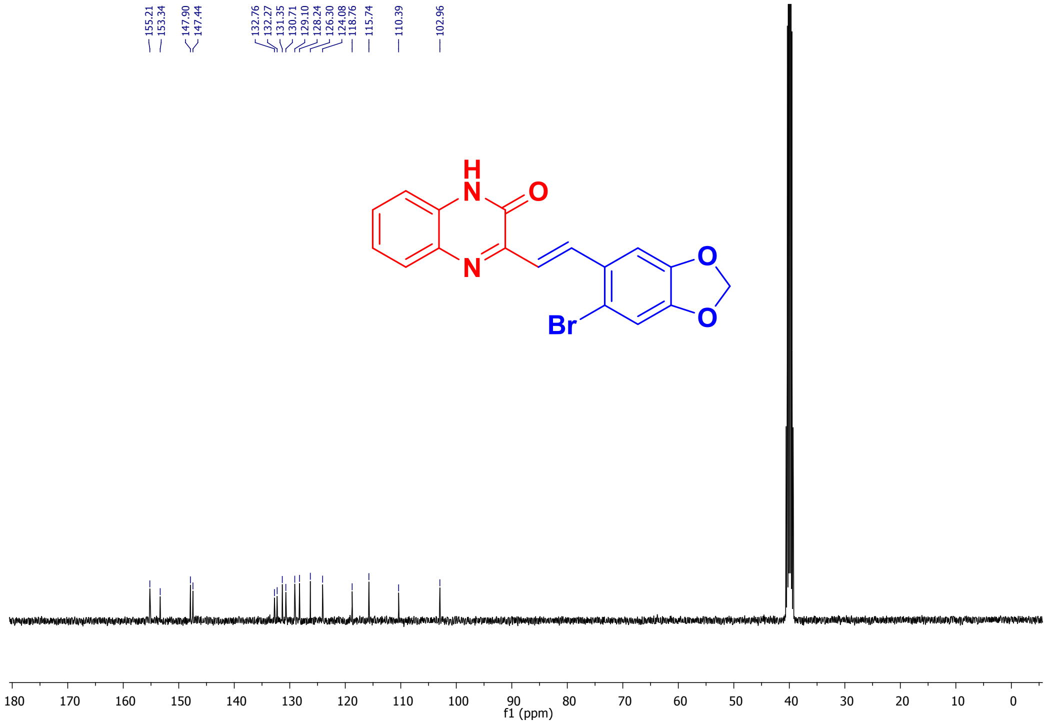

HPLC Analysis of **4w**

The HPLC purity was checked using Shimadzu HPLC system, consisting of purosphere C_{18} (5 µ, 250 × 4.6 mm) column and a PDA detector. The flow rate was 0.6 mL/min with the injection volume of 10 µL. The total run time was 45 min with gradient elution using 0.1% v/v formic acid in water (A) and mobile phase of acetonitrile (B). The gradient (WRT % v/v of A and B) was as shown in the **Table 2** :

Table 2 : Parameters used in HPLC purity check.

Time (in min.)	WRT % v/v of B	WRT % v/v of A
0	0	100
10	10	90
20	30	70
30	60	40
35	80	20
40	0	100
45	Stop	Stop

26-11-2019 10:03:25 1 / 1

==== Shimadzu LCsolution Analysis Report ====

C:\newcgmp\purity\4W.lcd

Acquired by : Admin
Sample Name : 4W
Sample ID : 4W
Tray# : 1
Vail # : 31
Injection Volume : 5 uL
Data File Name : 4W.lcd
Method File Name : purity.lcm
Batch File Name : nancy compound purity.lcb
Report File Name : Default.lcr
Data Acquired : 26-11-2019 03:08:47
Data Processed : 26-11-2019 10:02:45

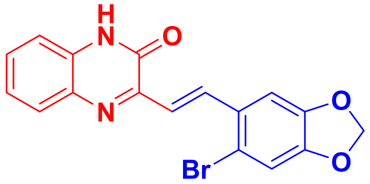

<Chromatogram>

C:\newcgmp\purity\4W.lcd

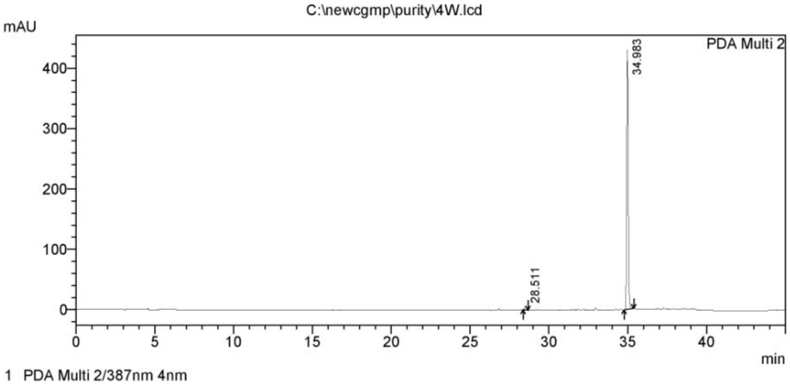

1 PDA Multi 2/387nm 4nm

PeakTable

PDA Ch2 387nm 4nm

Peak#	Ret. Time	Area	Height	Area %	Height %
1	28.511	45655	6005	1.615	1.376
2	34.983	2781664	430275	98.385	98.624
Total		2827319	436280	100.000	100.000

HRMS Analysis of **4w**

LC HRMS- THERMOSCIENTIFIC- EXACTIVE
C18 COLUMN- Hypersil
MOBILE PHASE- methanol and water (0.1% formic acid)
Gradient method : 97% methanol and 3% water for 5 minutes.
Injected amount : 2Microlitre
Flow rate of solvent 150µl /minute
The source was operated in both positive and negative mode at an ion spray voltage of 3KV
Oven temperature was set to 30^{0}C

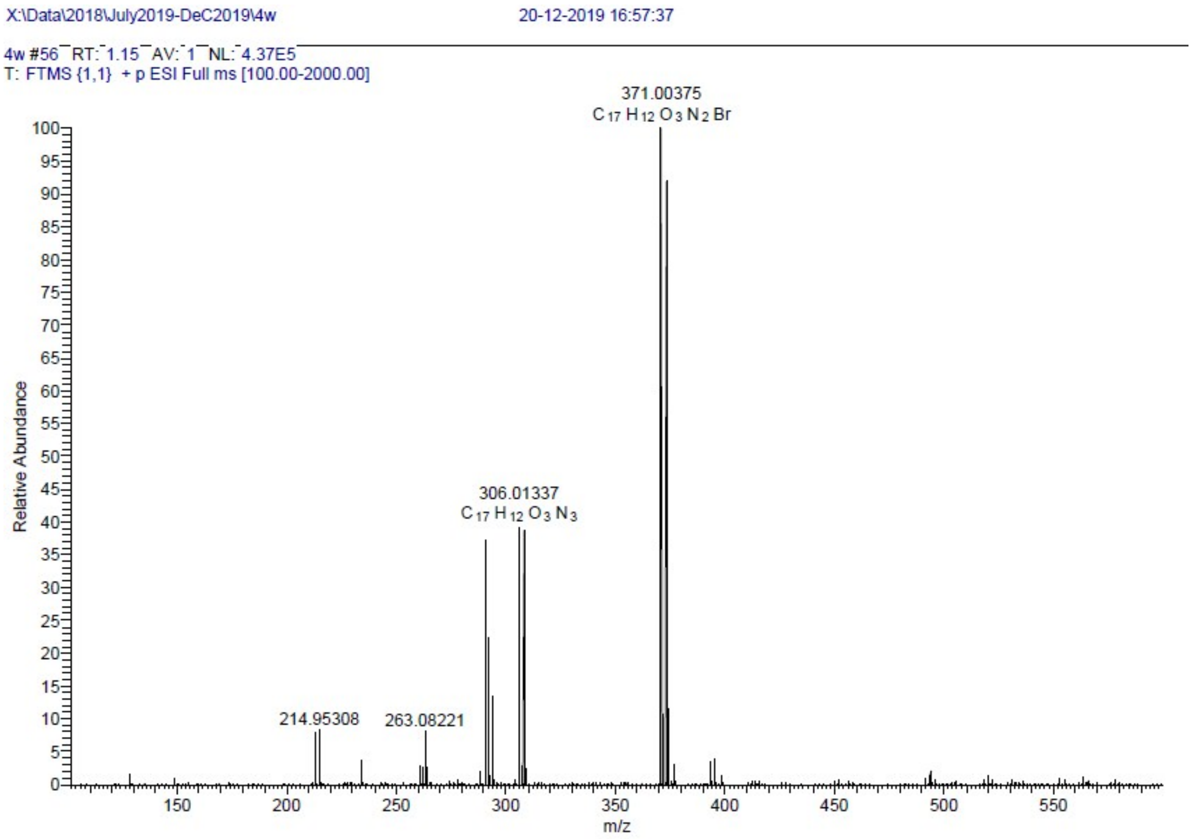

X:\Data\2018\July2019-DeC2019\4w 20-12-2019 16:57:37

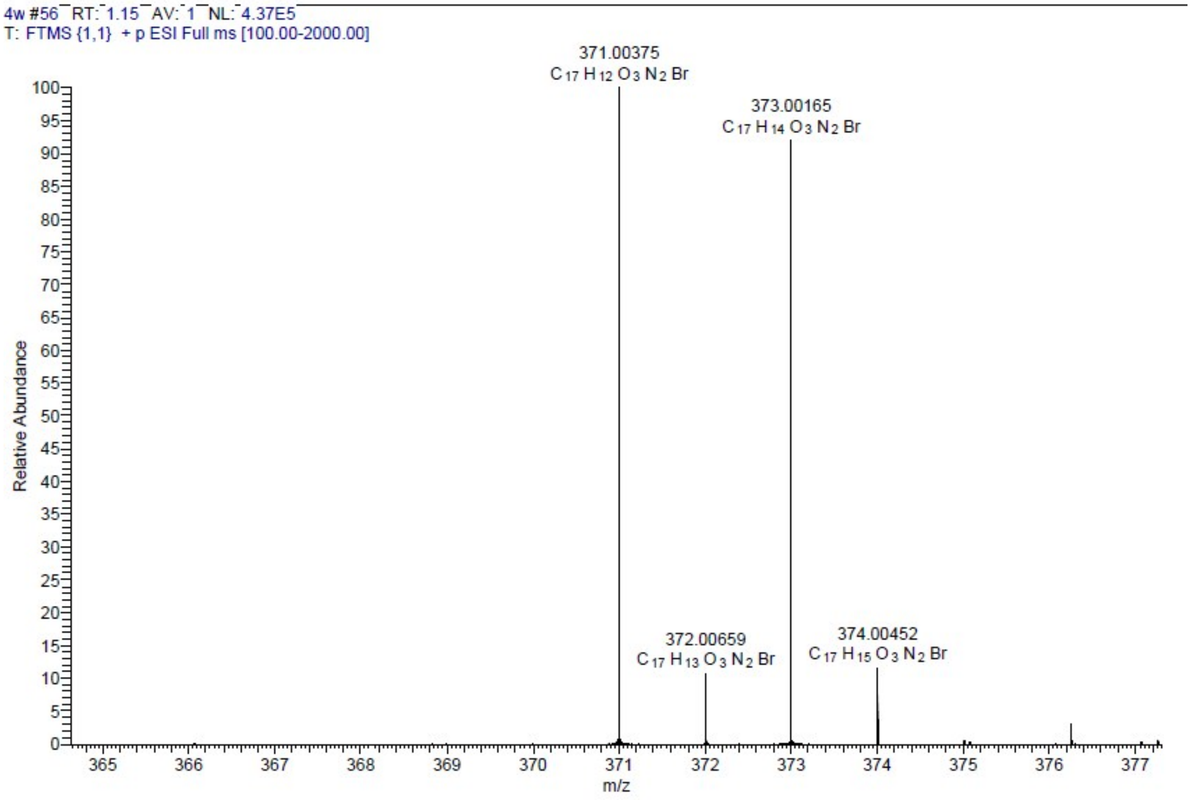

4x. *3-((1E,3E)-4-phenylbuta-1,3-dien-1-yl)quinoxalin-2(1H)-one*

<u>^{1}H NMR spectrum</u> (400 MHz, DMSO-d$_6$)

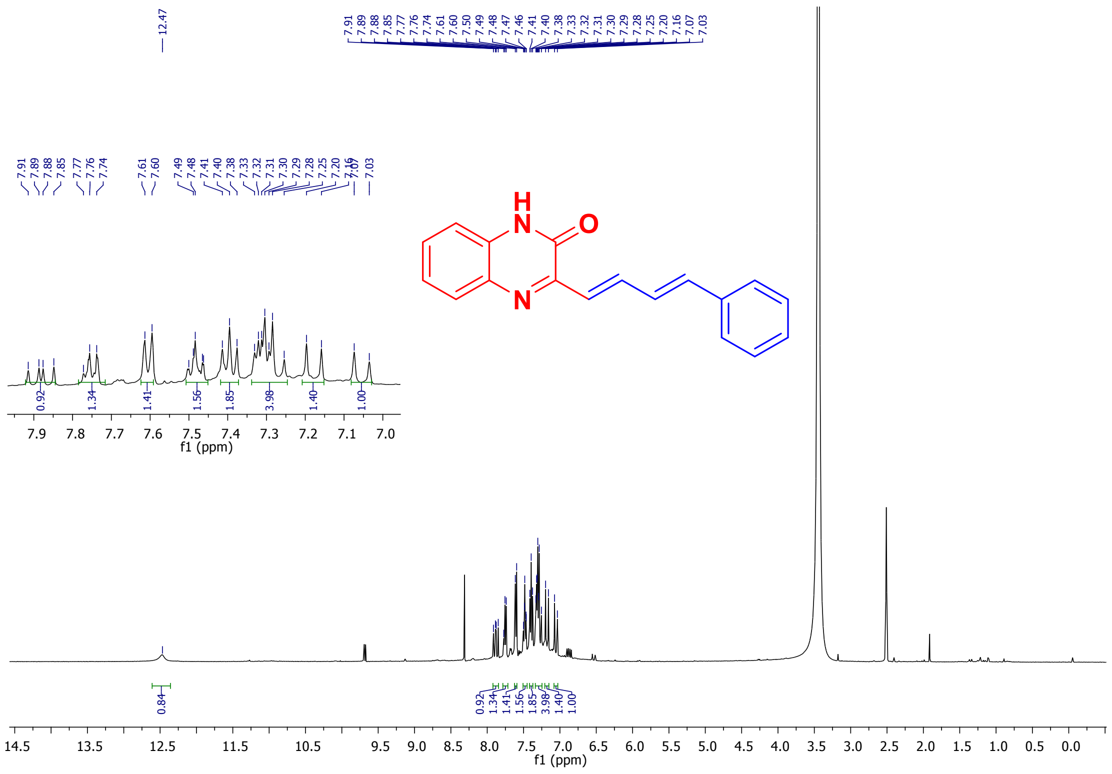

<u>^{13}C NMR spectrum</u> (101 MHz, DMSO-d$_6$)

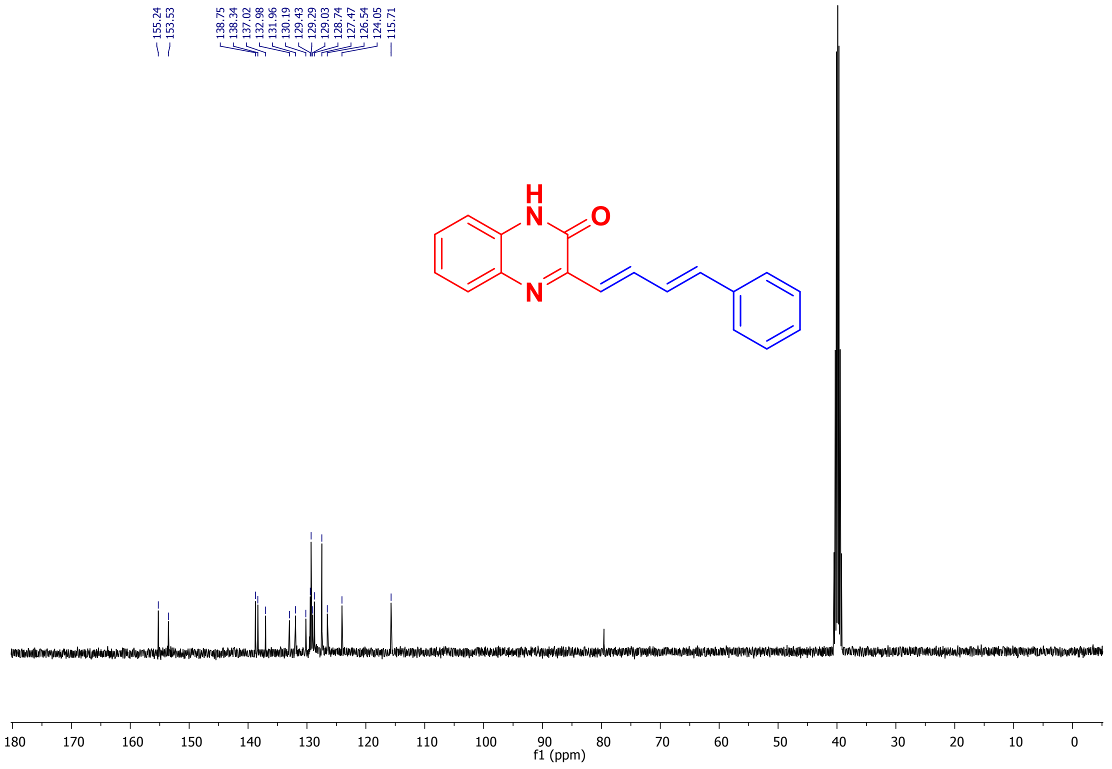

4y. ***(E)-3-(4-(trifluoromethyl)styryl)quinoxalin-2(1H)-one***

^{1}H NMR spectrum (400 MHz, DMSO-d$_6$)

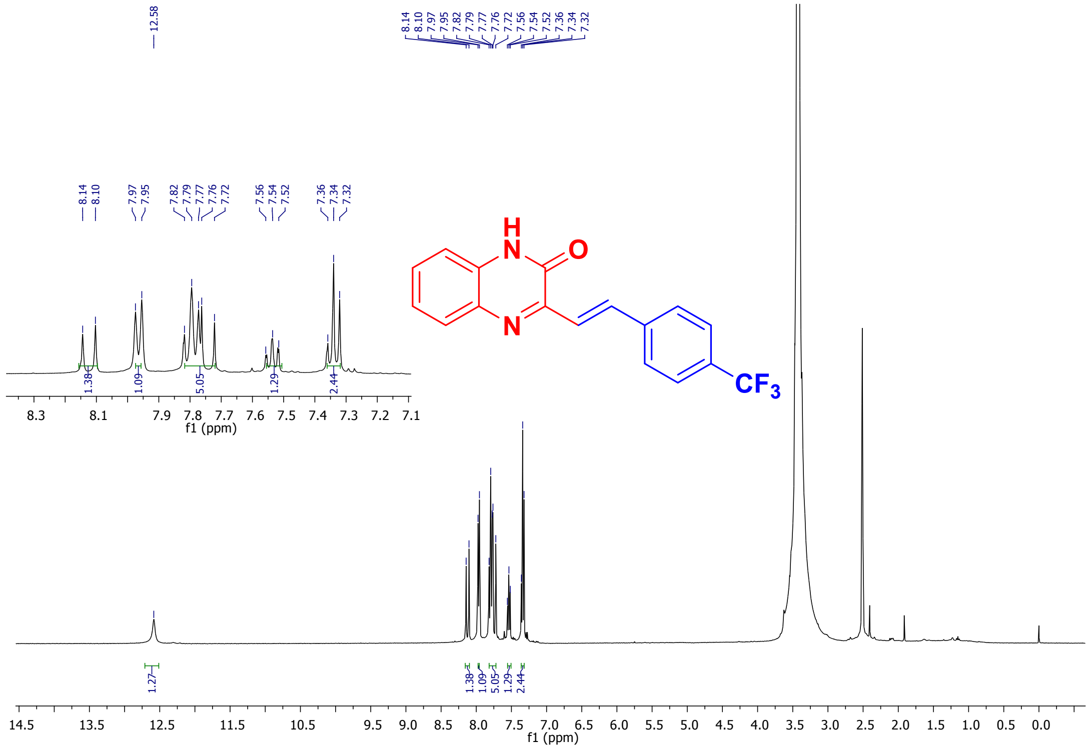

^{13}C NMR spectrum (101 MHz, DMSO-d$_6$)

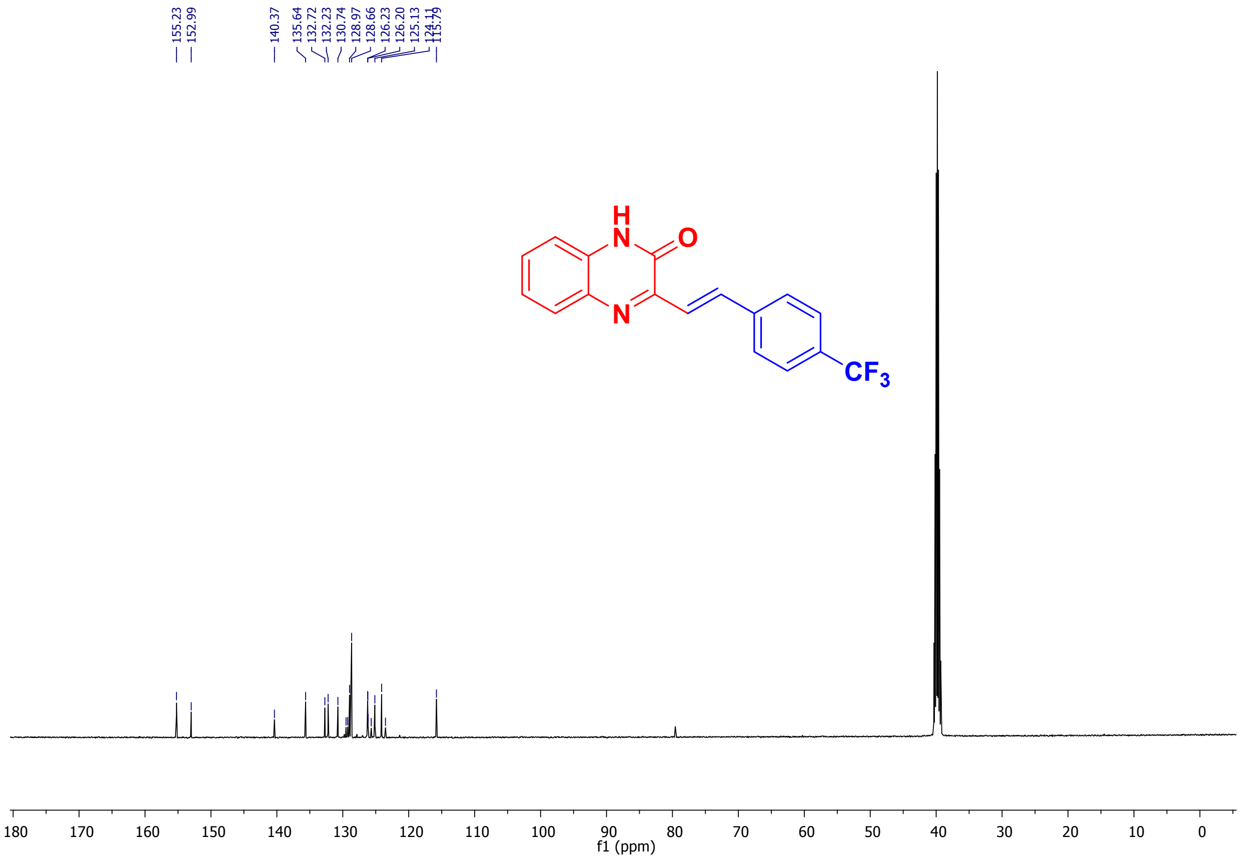

^{19}F NMR spectrum (376 MHz, DMSO-d_6)

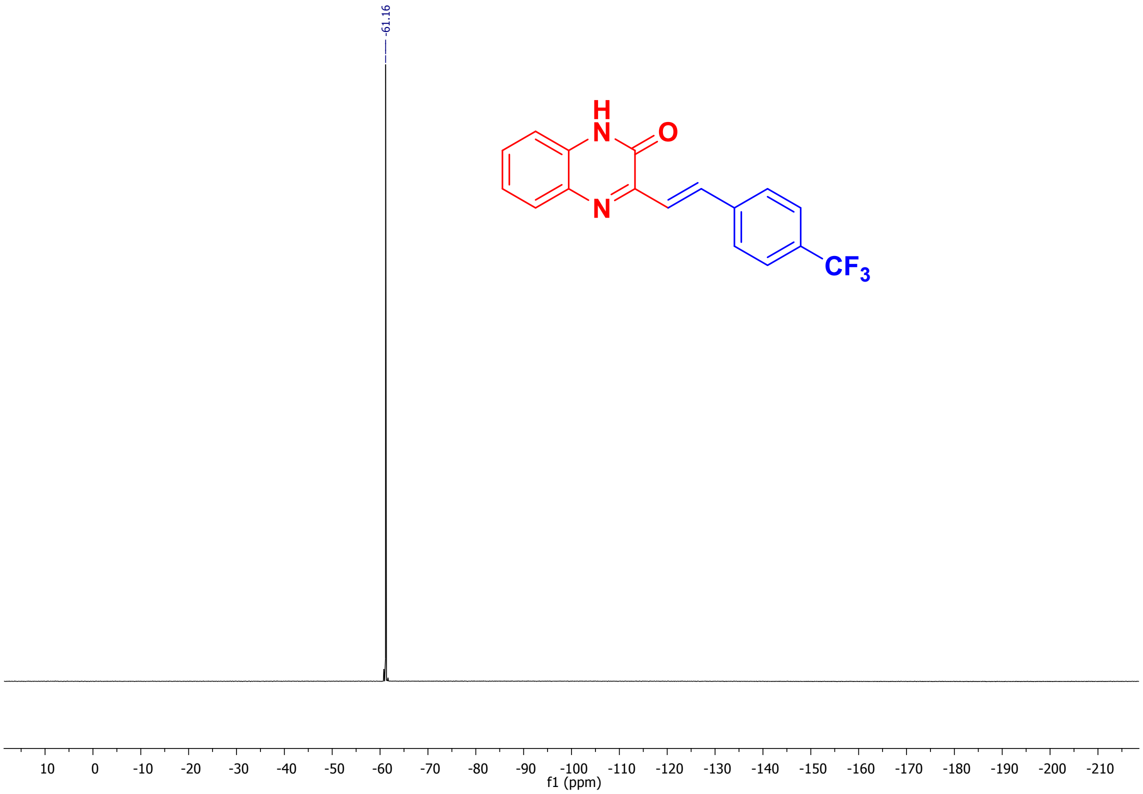

4z. *(E)-3-(4-hydroxy-3-methoxystyryl)quinoxalin-2(1H)-one*

^{1}H NMR spectrum (400 MHz, DMSO-d_6)

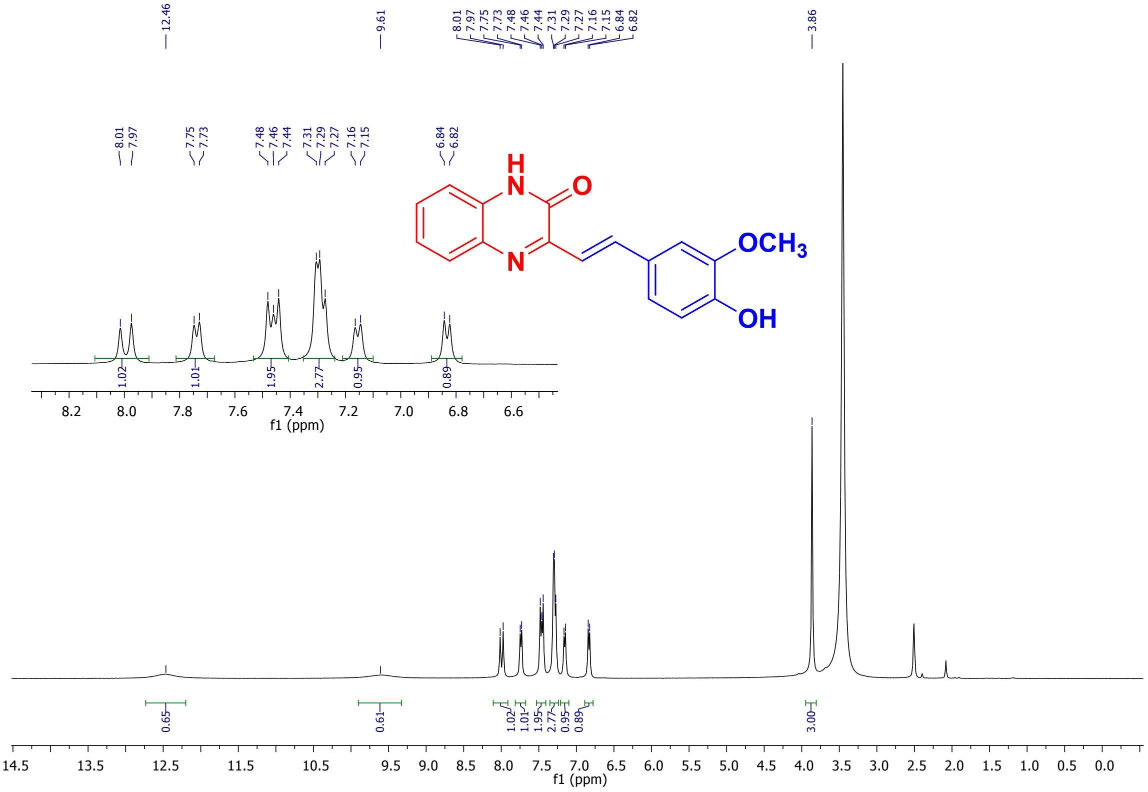

^{13}C NMR spectrum (101 MHz, DMSO-d_6)

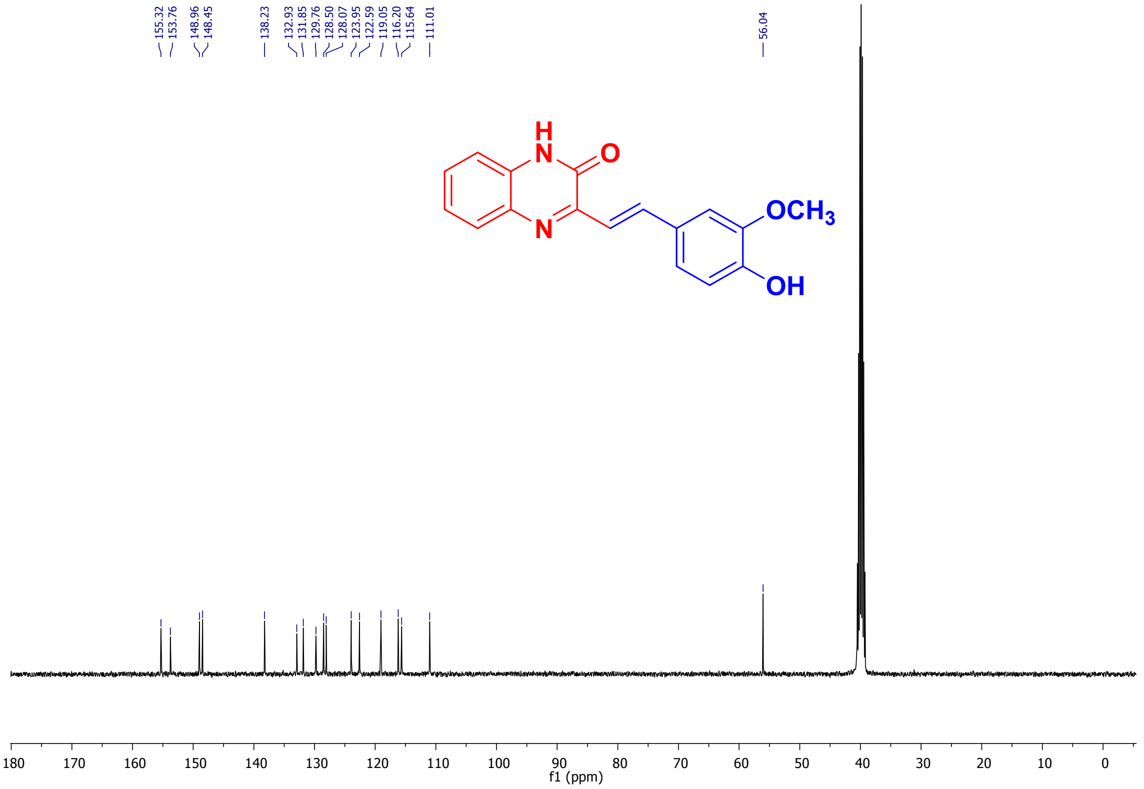

HPLC Analysis of **4z**

The HPLC purity was checked using Shimadzu HPLC system, consisting of purosphere C_{18} (5 µ, 250 × 4.6 mm) column and a PDA detector. The flow rate was 0.6 mL/min with the injection volume of 10 µL. The total run time was 45 min with gradient elution using 0.1% v/v formic acid in water (A) and mobile phase of acetonitrile (B). The gradient (WRT % v/v of A and B) was as shown in the **Table 2** :

Table 2 : Parameters used in HPLC purity check.

Time (in min.)	WRT % v/v of B	WRT % v/v of A
0	0	100
10	10	90
20	30	70
30	60	40
35	80	20
40	0	100
45	Stop	Stop

==== Shimadzu LCsolution Analysis Report ====

C:\newcgmp\purity\4z.lcd

Acquired by : Admin
Sample Name : 4z
Sample ID : 4z
Tray# : 1
Vail # : 67
Injection Volume : 5 uL
Data File Name : 4z.lcd
Method File Name : purity.lcm
Batch File Name : 17032020.lcb
Report File Name : Default.lcr
Data Acquired : 17-03-2020 12:24:19
Data Processed : 17-03-2020 13:09:21

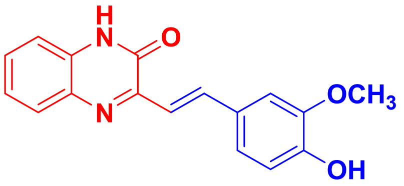

<Chromatogram>

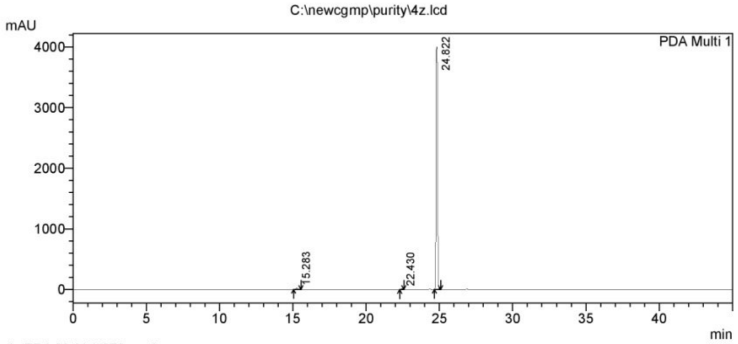

1 PDA Multi 1/272nm 4nm

PeakTable

PDA Ch1 272nm 4nm

Peak#	Ret. Time	Area	Height	Area %	Height %
1	15.283	196459	24630	0.707	0.610
2	22.430	73917	10169	0.266	0.252
3	24.822	27531482	3999780	99.027	99.137
Total		27801858	4034579	100.000	100.000

HRMS Analysis of **4z**

LC HRMS- THERMOSCIENTIFIC- EXACTIVE
C18 COLUMN- Hypersil
MOBILE PHASE- methanol and water (0.1% formic acid)
Gradient method : 97% methanol and 3% water for 5 minutes.
Injected amount : 2Microlitre
Flow rate of solvent   150µl /minute
The source was operated in both positive and negative mode at an ion spray voltage of 3KV
Oven temperature was set to 30^{0}C

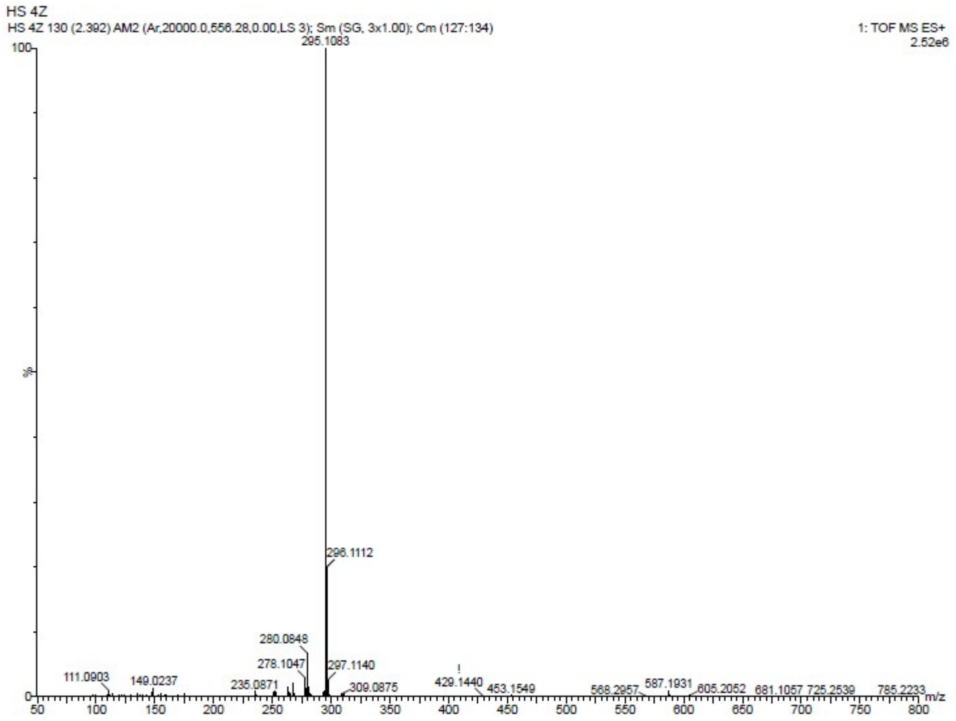

4aa. *(E)-3-(3-hydroxy-4-methoxystyryl)quinoxalin-2(1H)-one*

^{1}H NMR spectrum (400 MHz, DMSO-d_6)

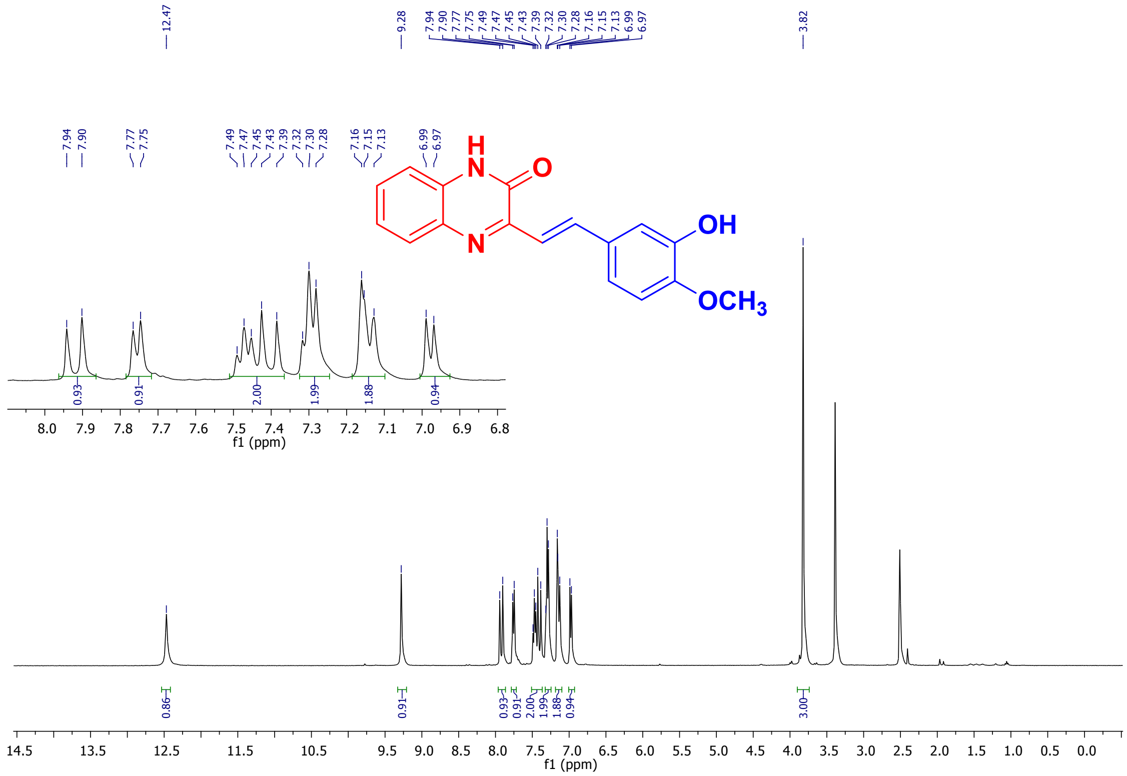

^{13}C NMR spectrum (101 MHz, DMSO-d_6)

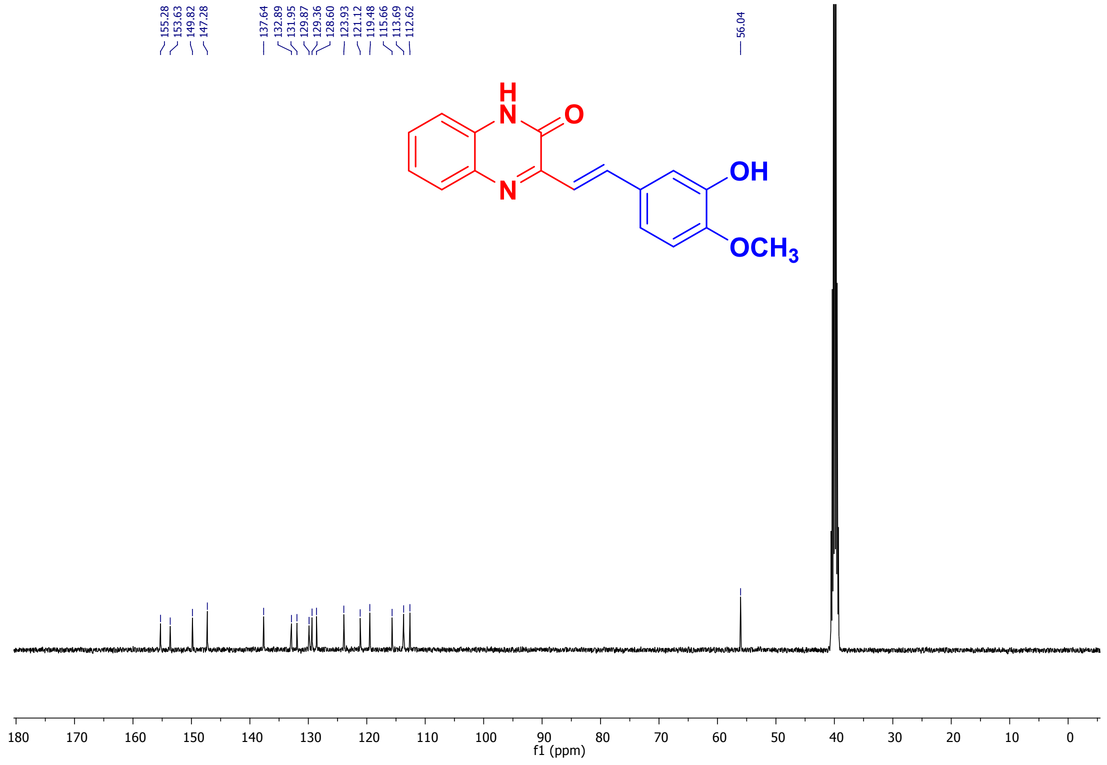

4ab. *(E)-3-(2-hydroxy-3-methoxystyryl)quinoxalin-2(1H)-one*

^{1}H NMR spectrum (400 MHz, DMSO-d$_6$)

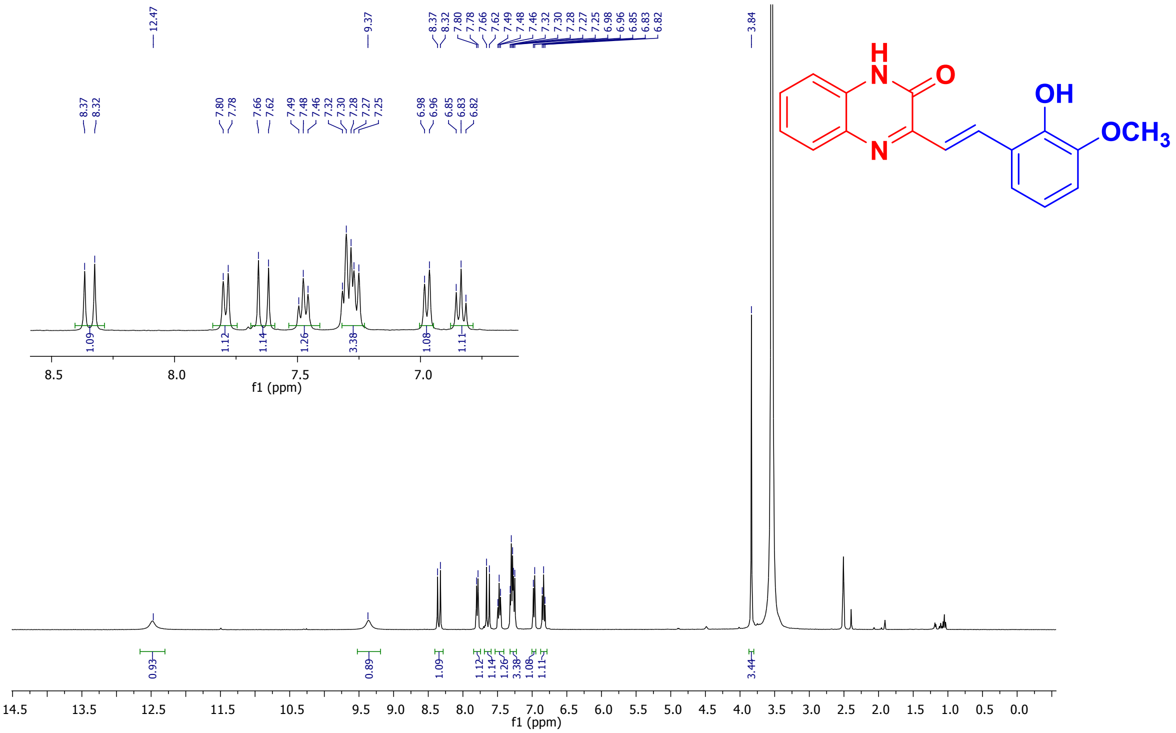

^{13}C NMR spectrum (101 MHz, DMSO-d$_6$)

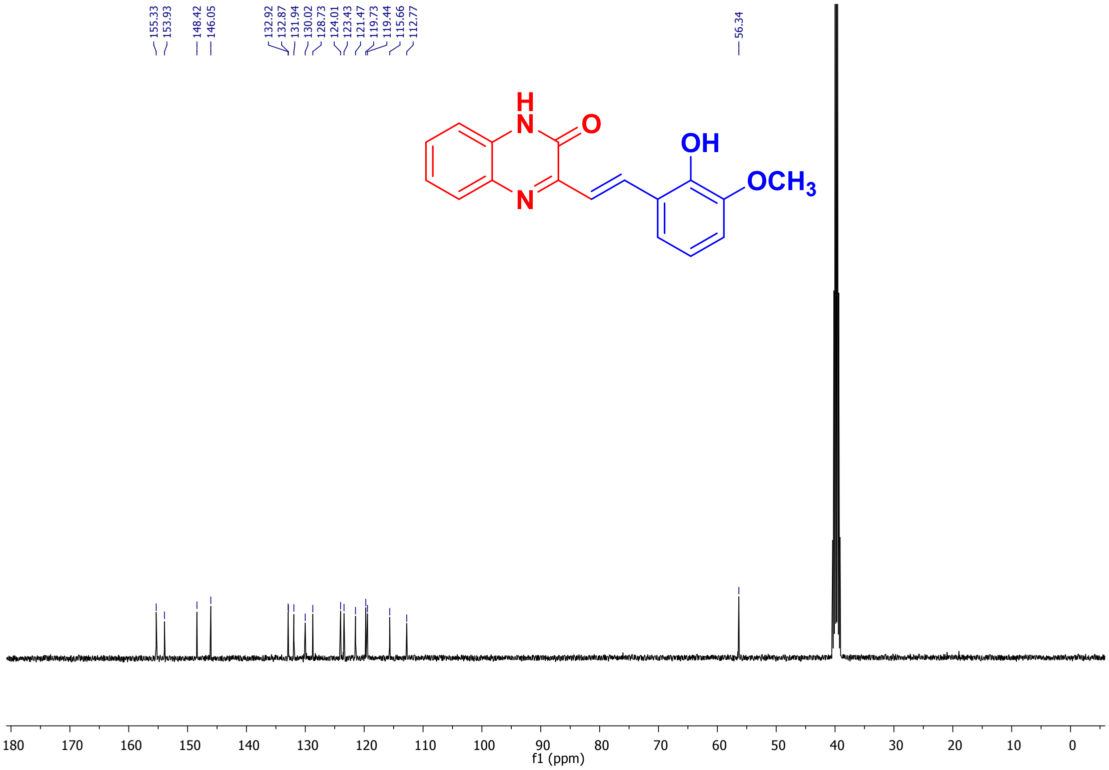

HPLC Analysis of **4ab**

The HPLC purity was checked using Shimadzu HPLC system, consisting of purosphere C_{18} (5 μ, 250 × 4.6 mm) column and a PDA detector. The flow rate was 0.6 mL/min with the injection volume of 10 μL. The total run time was 45 min with gradient elution using 0.1% v/v formic acid in water (A) and mobile phase of acetonitrile (B). The gradient (WRT % v/v of A and B) was as shown in the **Table 2** :

Table 2 : Parameters used in HPLC purity check.

**Time (in min.)**	**WRT % v/v of B**	**WRT % v/v of A**
0	0	100
10	10	90
20	30	70
30	60	40
35	80	20
40	0	100
45	Stop	Stop

==== Shimadzu LCsolution Analysis Report ====

C:\newcgmp\purity\4ab.lcd

Acquired by	: Admin
Sample Name	: 4ab
Sample ID	: 4ab
Tray#	: 1
Vail #	: 65
Injection Volume	: 5 uL
Data File Name	: 4ab.lcd
Method File Name	: purity.lcm
Batch File Name	: 17032020.lcb
Report File Name	: Default.lcr
Data Acquired	: 17-03-2020 10:53:22
Data Processed	: 17-03-2020 11:38:25

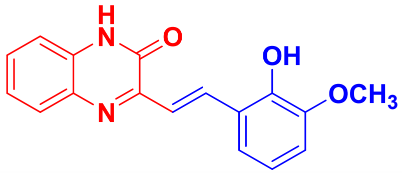

<Chromatogram>

C:\newcgmp\purity\4ab.lcd

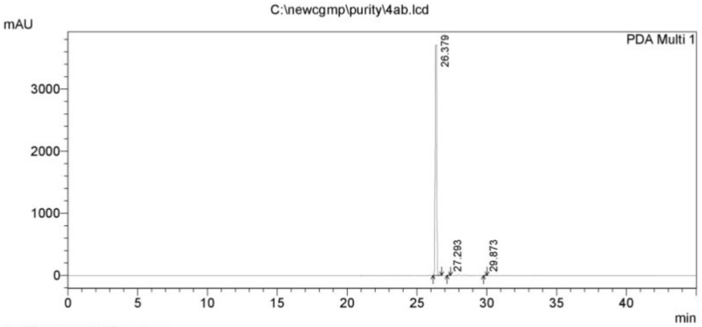

1 PDA Multi 1/387nm 4nm

PeakTable

PDA Ch1 387nm 4nm

Peak#	Ret. Time	Area	Height	Area %	Height %
1	26.379	29234547	3715062	99.275	99.264
2	27.293	120863	15285	0.410	0.408
3	29.873	92743	12260	0.315	0.328
Total		29448154	3742607	100.000	100.000

HRMS Analysis of **4ab**

LC HRMS- THERMOSCIENTIFIC- EXACTIVE
C18 COLUMN- Hypersil
MOBILE PHASE- methanol and water (0.1% formic acid)
Gradient method : 97% methanol and 3% water for 5 minutes.
Injected amount : 2Microlitre
Flow rate of solvent 150µl /minute
The source was operated in both positive and negative mode at an ion spray voltage of 3KV
Oven temperature was set to 30^{0}C

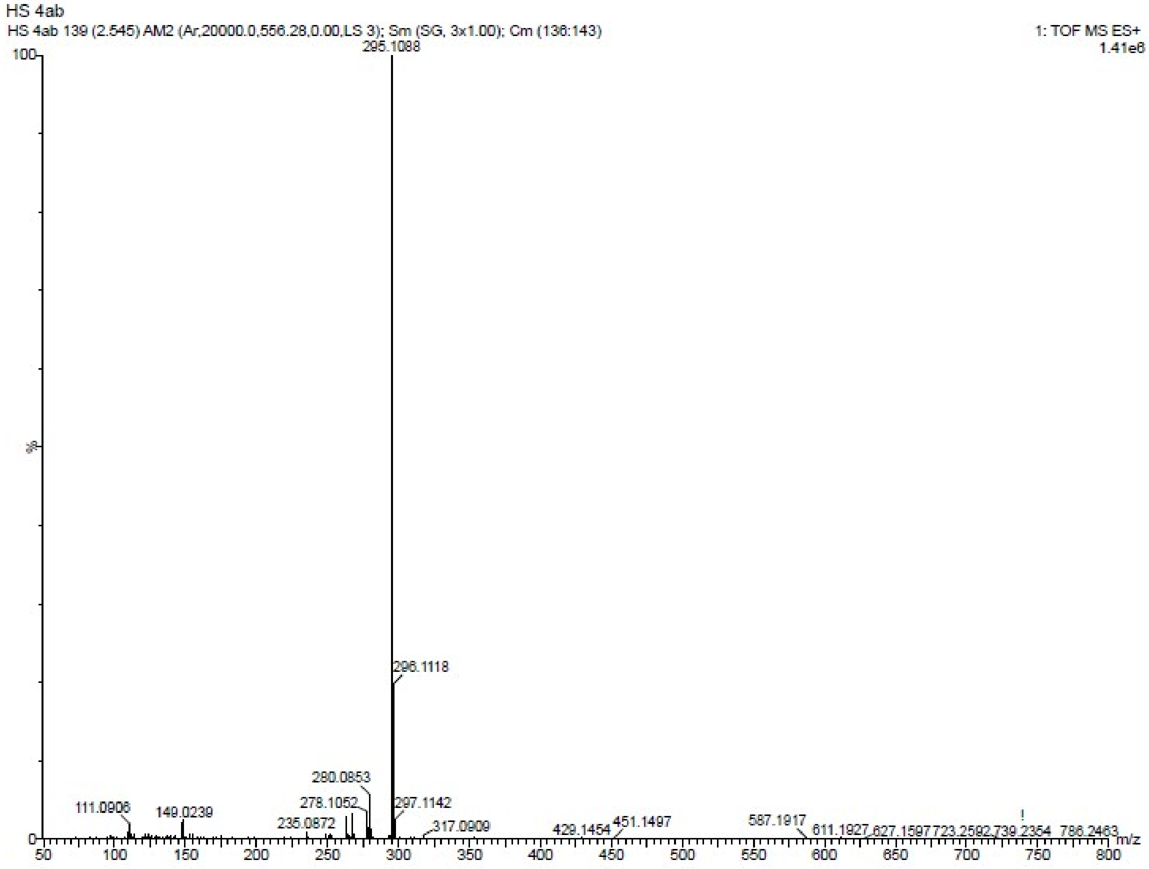

4ac. *(E)-3-(3-ethoxy-4-hydroxystyryl)quinoxalin-2(1H)-one*

^{1}H NMR spectrum (400 MHz, DMSO-d$_6$)

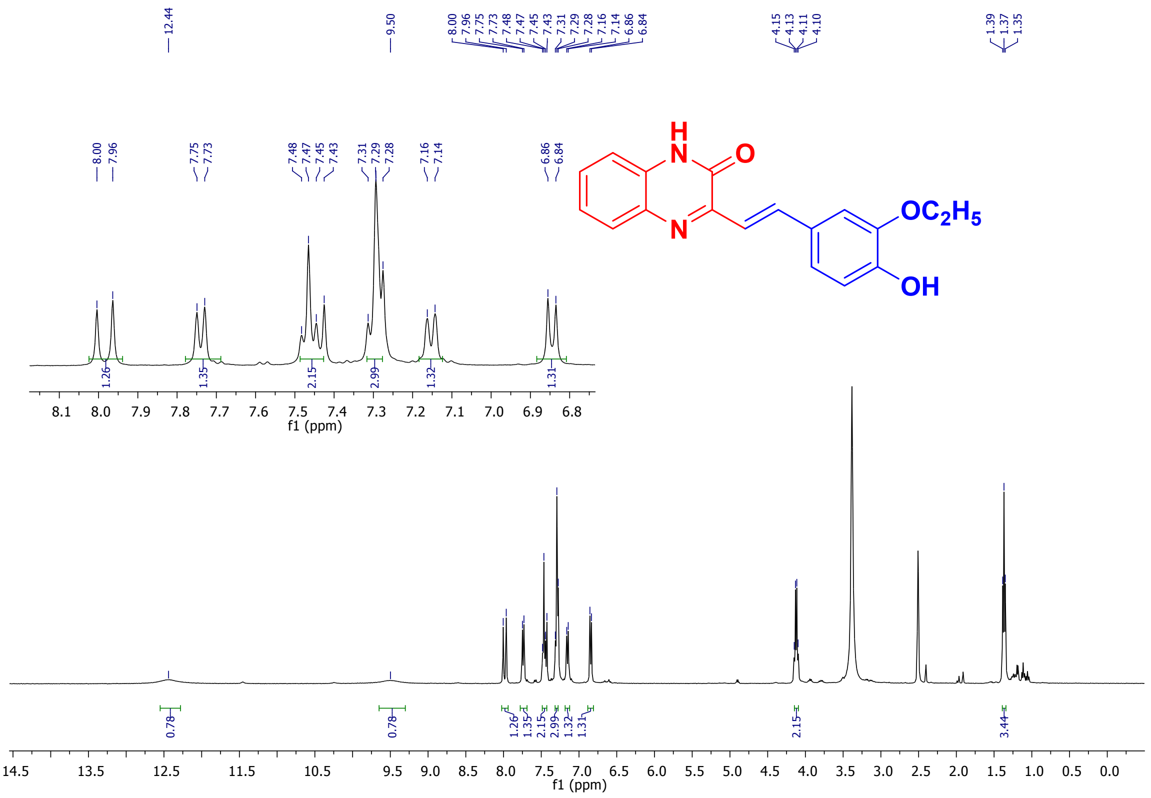

^{13}C NMR spectrum (101 MHz, DMSO-d$_6$)

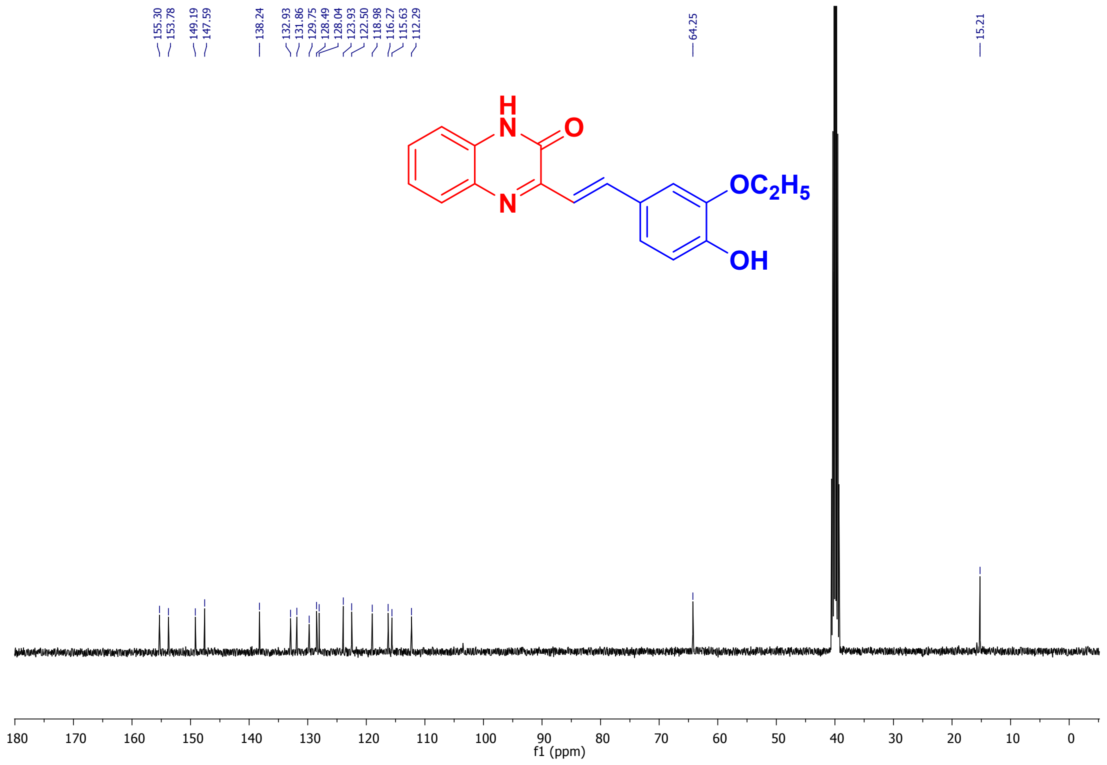

HPLC Analysis of **4ac**

The HPLC purity was checked using Shimadzu HPLC system, consisting of purosphere C_{18} (5 µ, 250 × 4.6 mm) column and a PDA detector. The flow rate was 0.6 mL/min with the injection volume of 10 µL. The total run time was 45 min with gradient elution using 0.1% v/v formic acid in water (A) and mobile phase of acetonitrile (B). The gradient (WRT % v/v of A and B) was as shown in the **Table 2** :

Table 2 : Parameters used in HPLC purity check.

Time (in min.)	WRT % v/v of B	WRT % v/v of A
0	0	100
10	10	90
20	30	70
30	60	40
35	80	20
40	0	100
45	Stop	Stop

19-03-2020 15:13:27 1 / 1

==== Shimadzu LCsolution Analysis Report ====

C:\newcgmp\purity\19-4ac.lcd

Acquired by	: Admin
Sample Name	: 19-4ac
Sample ID	: 19-4ac
Tray#	: 1
Vail #	: 19
Injection Volume	: 5 uL
Data File Name	: 19-4ac.lcd
Method File Name	: purity.lcm
Batch File Name	:
Report File Name	: Default.lcr
Data Acquired	: 19-03-2020 14:21:38
Data Processed	: 19-03-2020 15:06:39

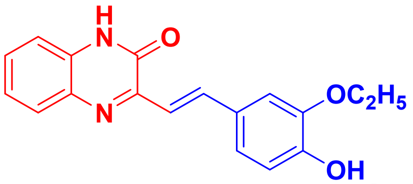

<Chromatogram>

C:\newcgmp\purity\19-4ac.lcd

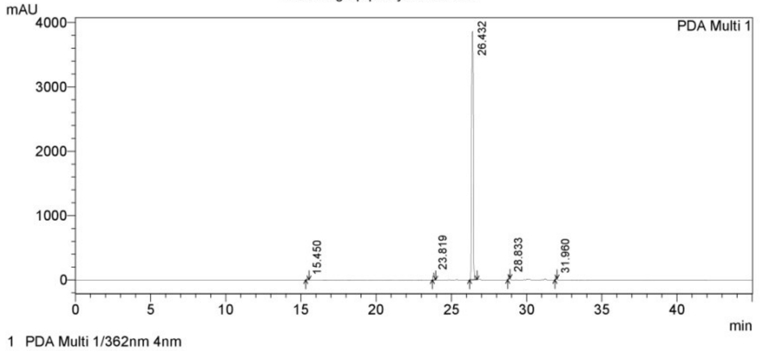

1 PDA Multi 1/362nm 4nm

PeakTable

PDA Ch1 362nm 4nm

Peak#	Ret. Time	Area	Height	Area %	Height %
1	15.450	215756	30873	0.668	0.752
2	23.819	597720	109428	1.850	2.665
3	26.432	30994112	3858272	95.911	93.980
4	28.833	293771	61265	0.909	1.492
5	31.960	214089	45596	0.662	1.111
Total		32315449	4105434	100.000	100.000

HRMS Analysis of **4ac**

LC HRMS- THERMOSCIENTIFIC- EXACTIVE
C18 COLUMN- Hypersil
MOBILE PHASE- methanol and water (0.1% formic acid)
Gradient method : 97% methanol and 3% water for 5 minutes.
Injected amount : 2Microlitre
Flow rate of solvent 150µl /minute
The source was operated in both positive and negative mode at an ion spray voltage of 3KV
Oven temperature was set to 30^{0}C

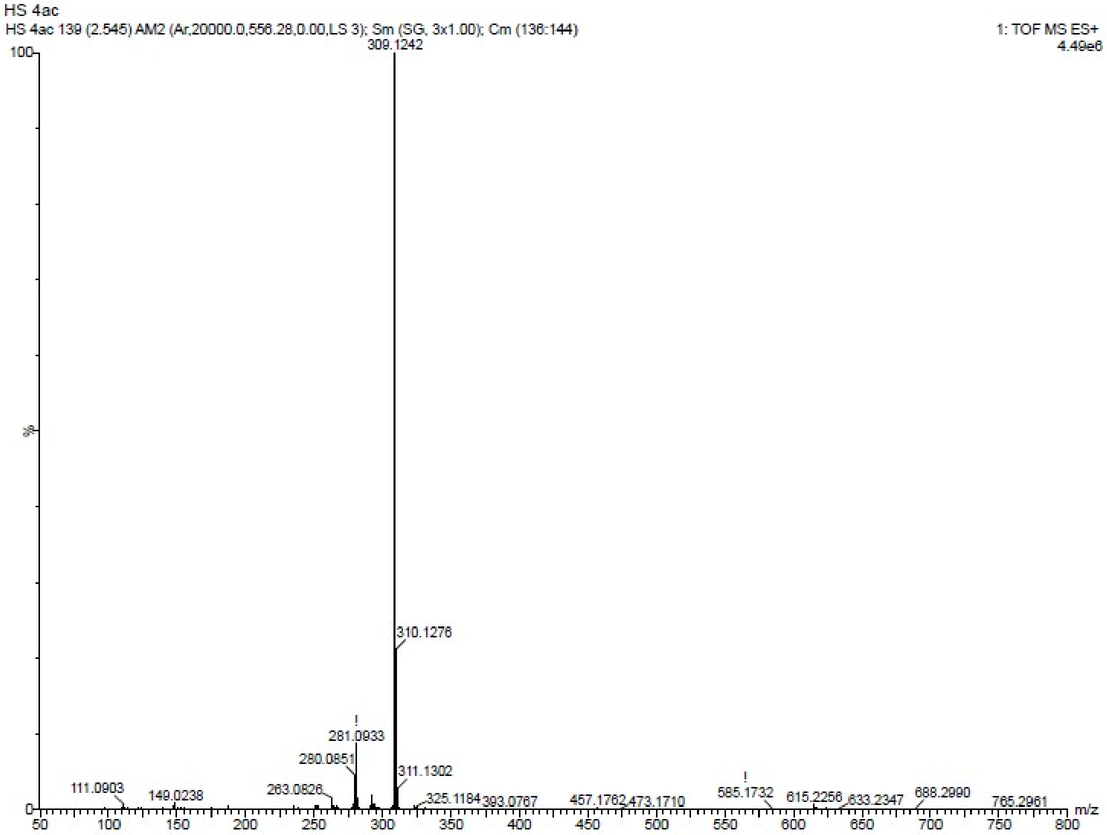

4ad. *(E)-3-(4-fluorostyryl)quinoxalin-2(1H)-one*

^{1}H NMR spectrum (400 MHz, DMSO-d$_6$)

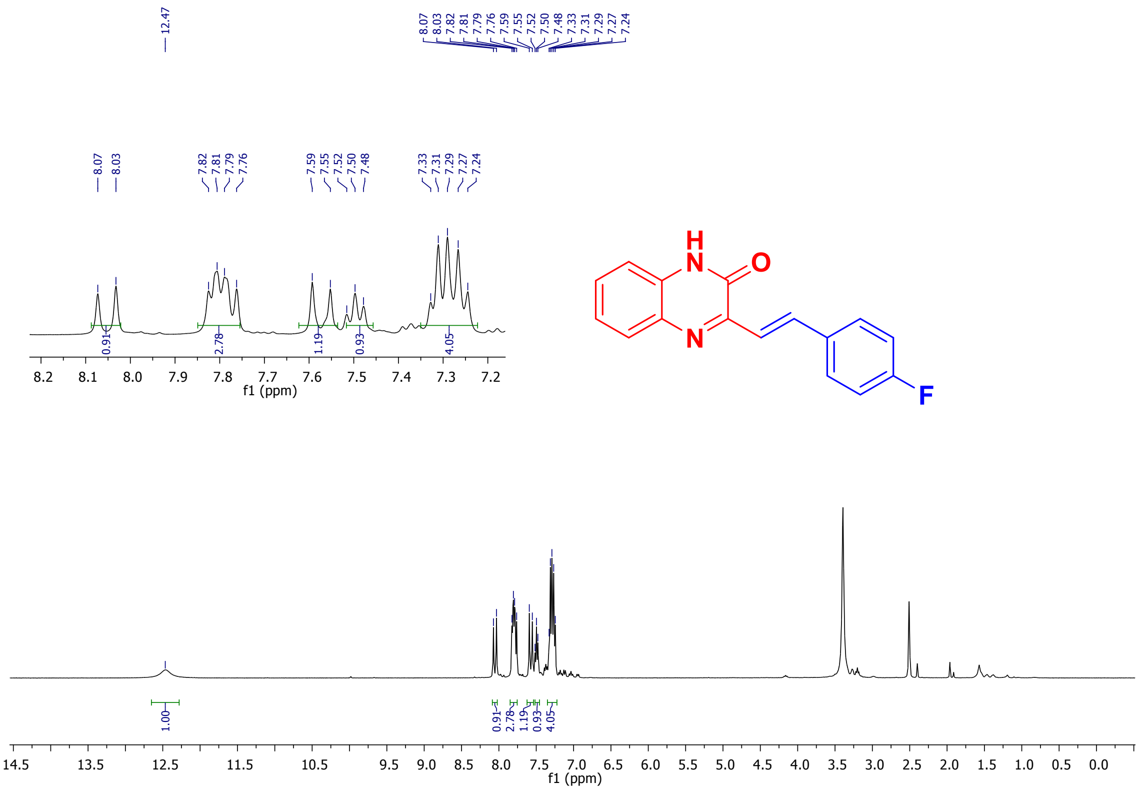

^{13}C NMR spectrum (101 MHz, DMSO-d$_6$)

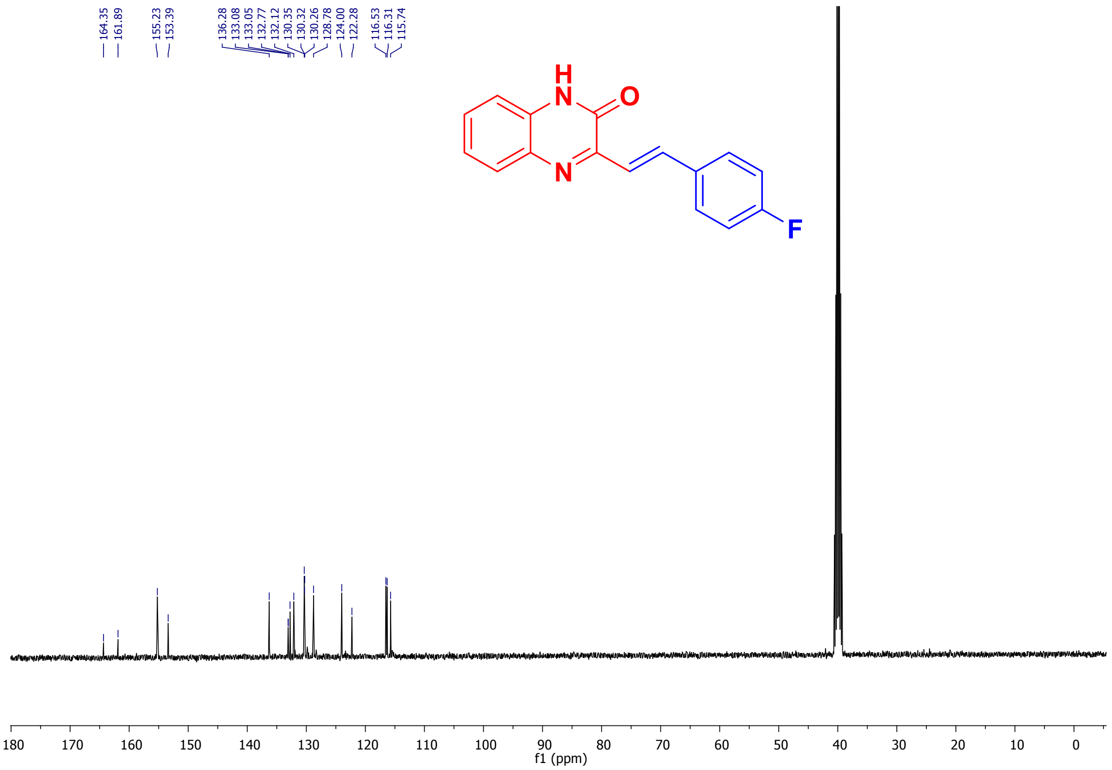

4ae. *(E)-2-styrylquinoxaline*

^{1}H NMR spectrum (400 MHz, $CDCl_3$)

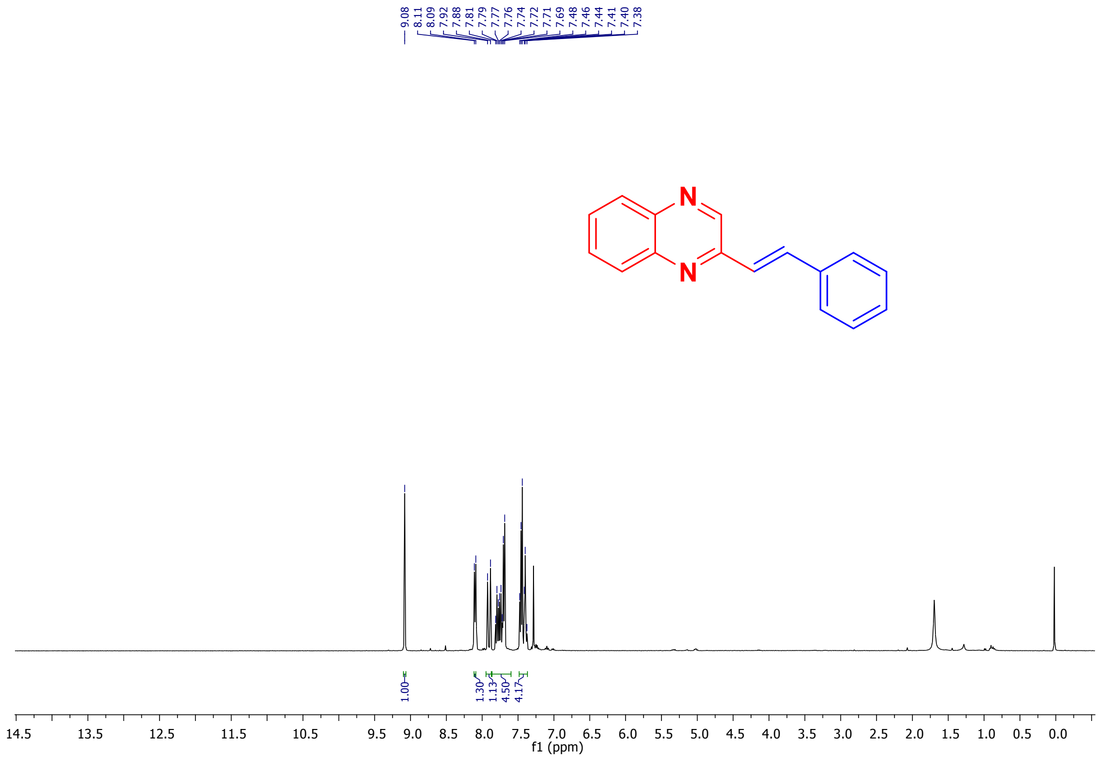

^{13}C NMR spectrum (101 MHz, $CDCl_3$)

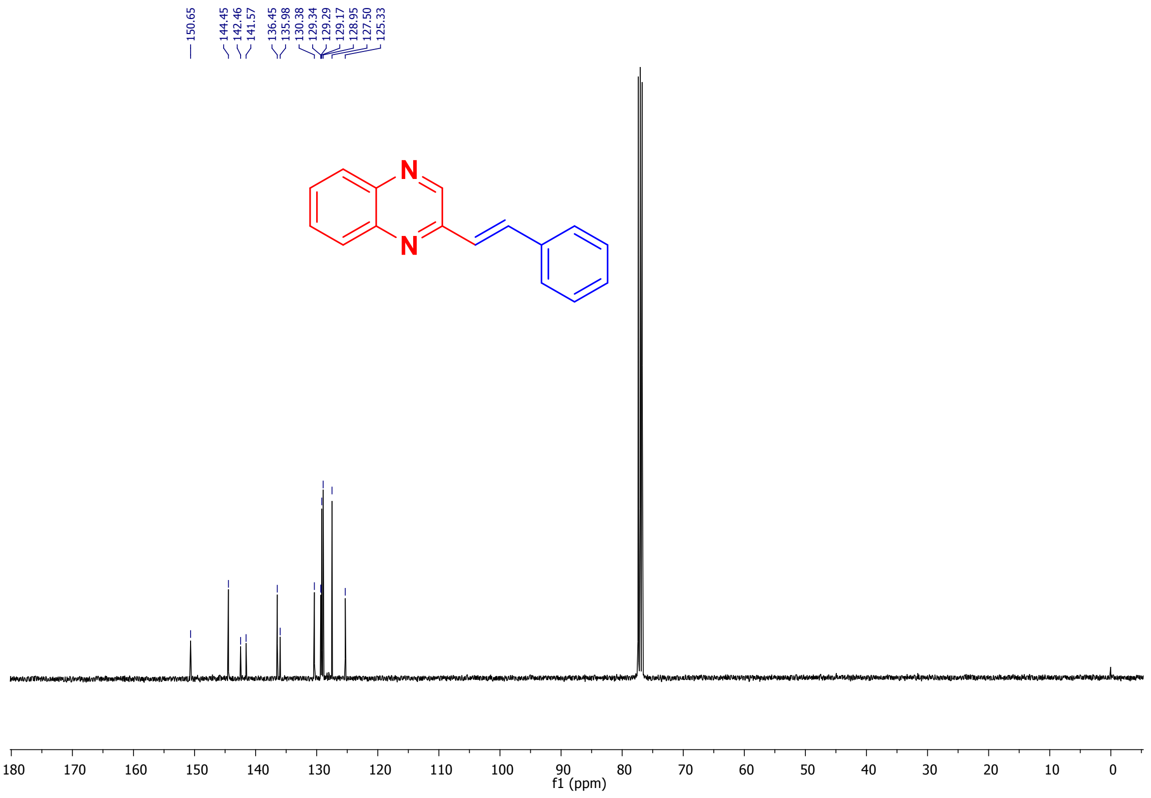

4af. *(E)-2-(4-methoxystyryl)quinoxaline*

<u>^{1}H NMR spectrum</u> (400 MHz, $CDCl_3$)

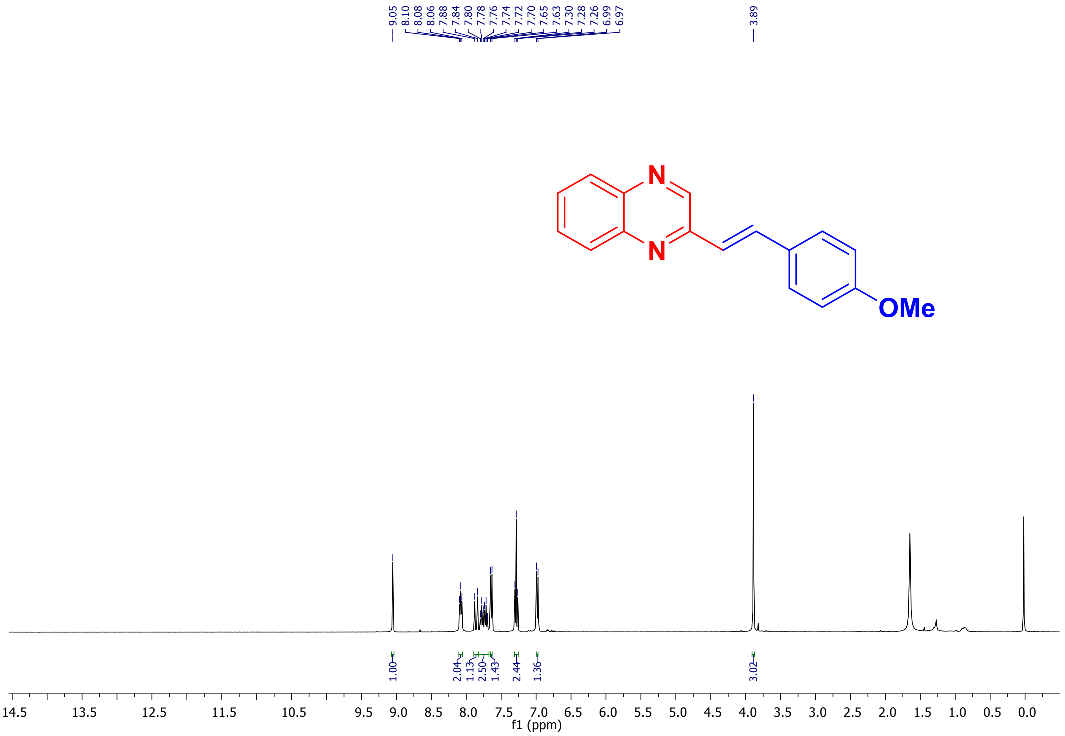

<u>^{13}C NMR spectrum</u> (101 MHz, $CDCl_3$)

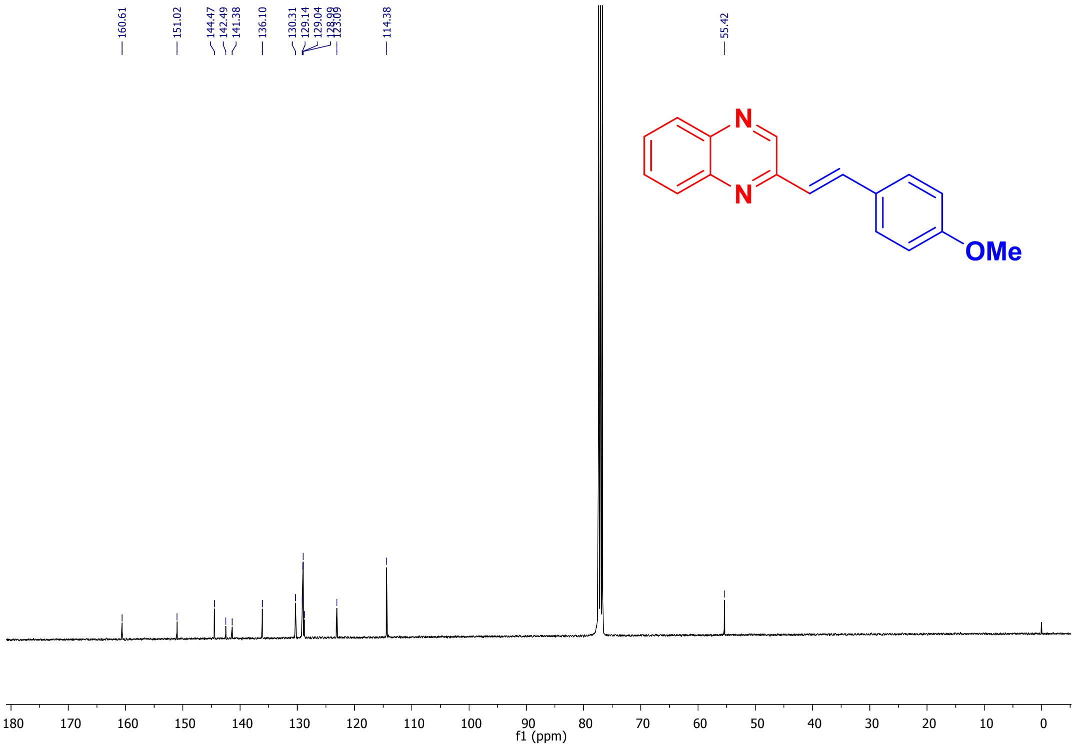

4ag. *(E)-2-(3,4-dimethoxystyryl)quinoxaline*

<u>^{1}H NMR spectrum</u> (400 MHz, $CDCl_3$)

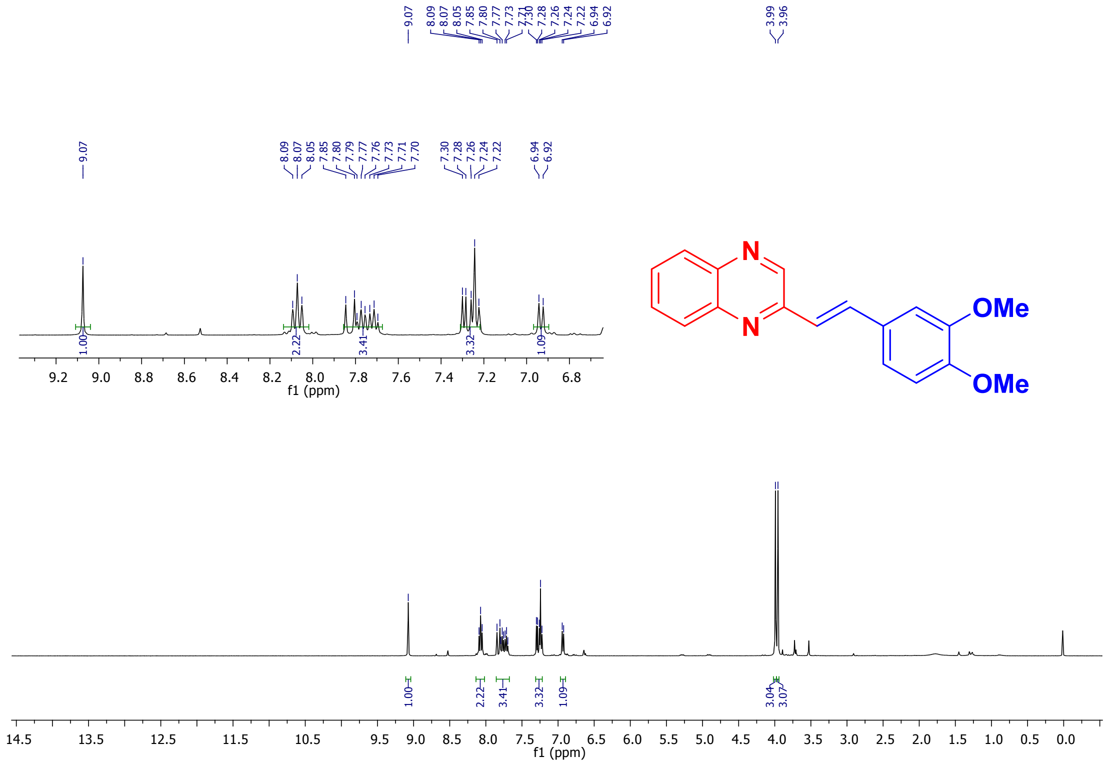

<u>^{13}C NMR spectrum</u> (101 MHz, $CDCl_3$)

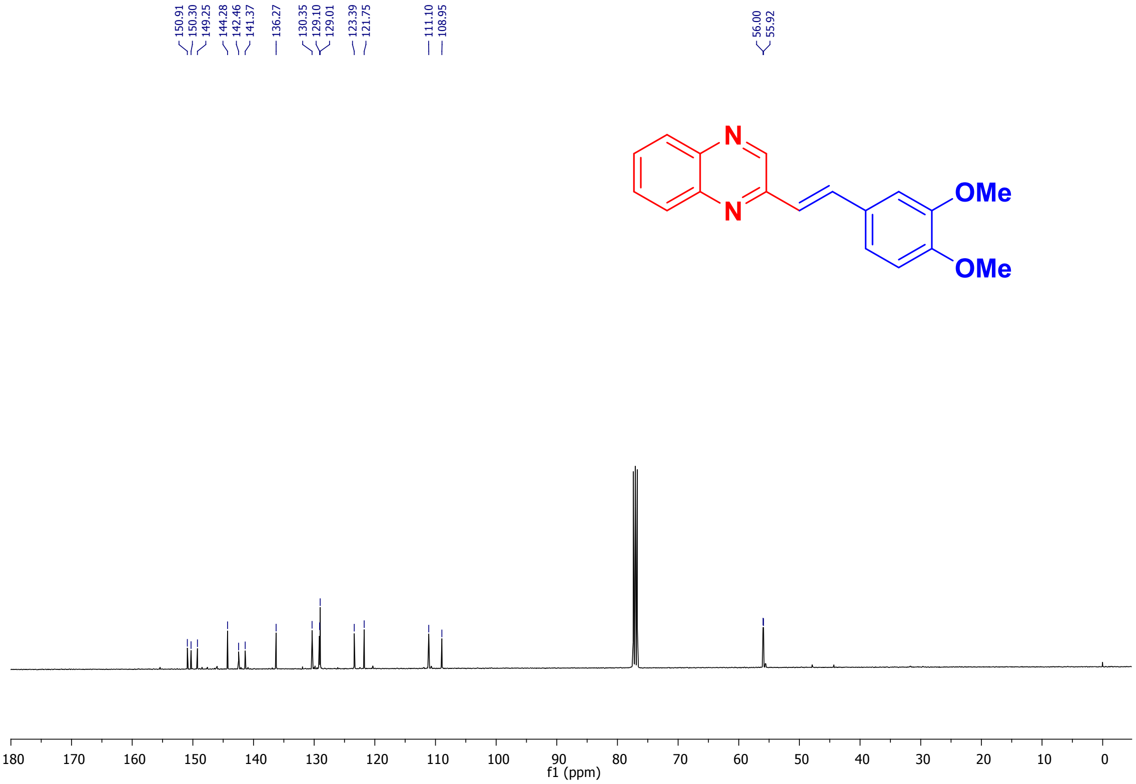

4ah. *(E)-2-styrylquinoline*

<u>^{1}H NMR spectrum</u> (400 MHz, DMSO-d$_6$)

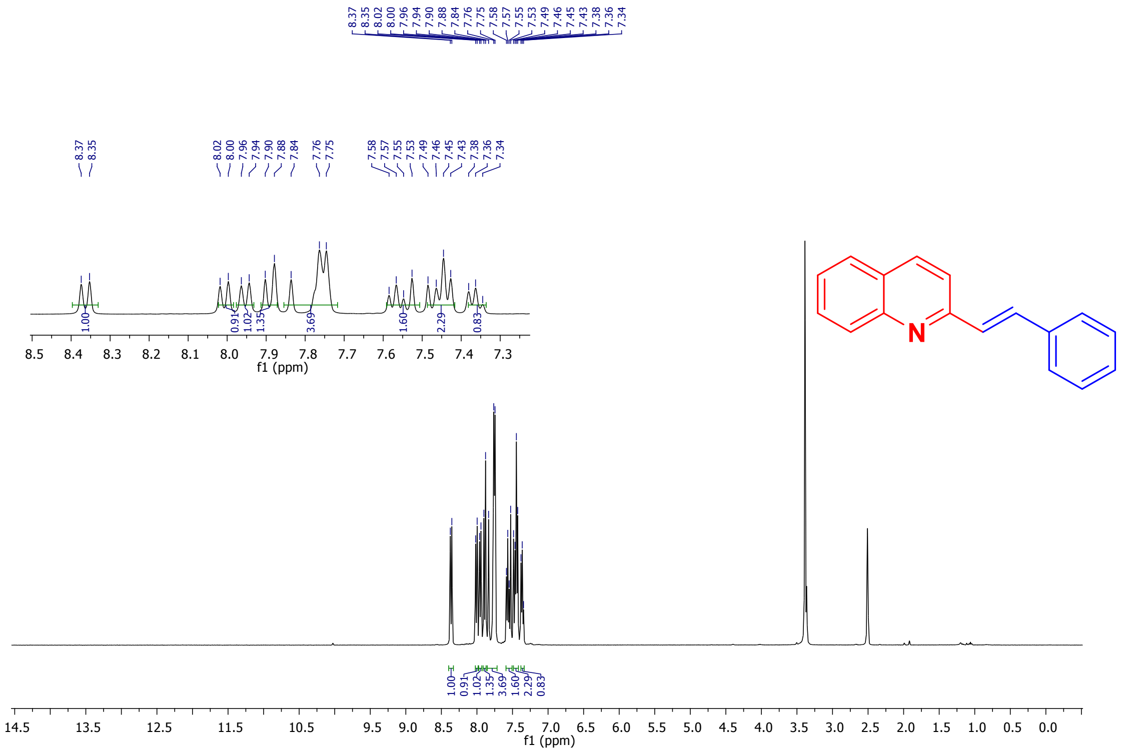

<u>^{13}C NMR spectrum</u> (101 MHz, DMSO-d$_6$)

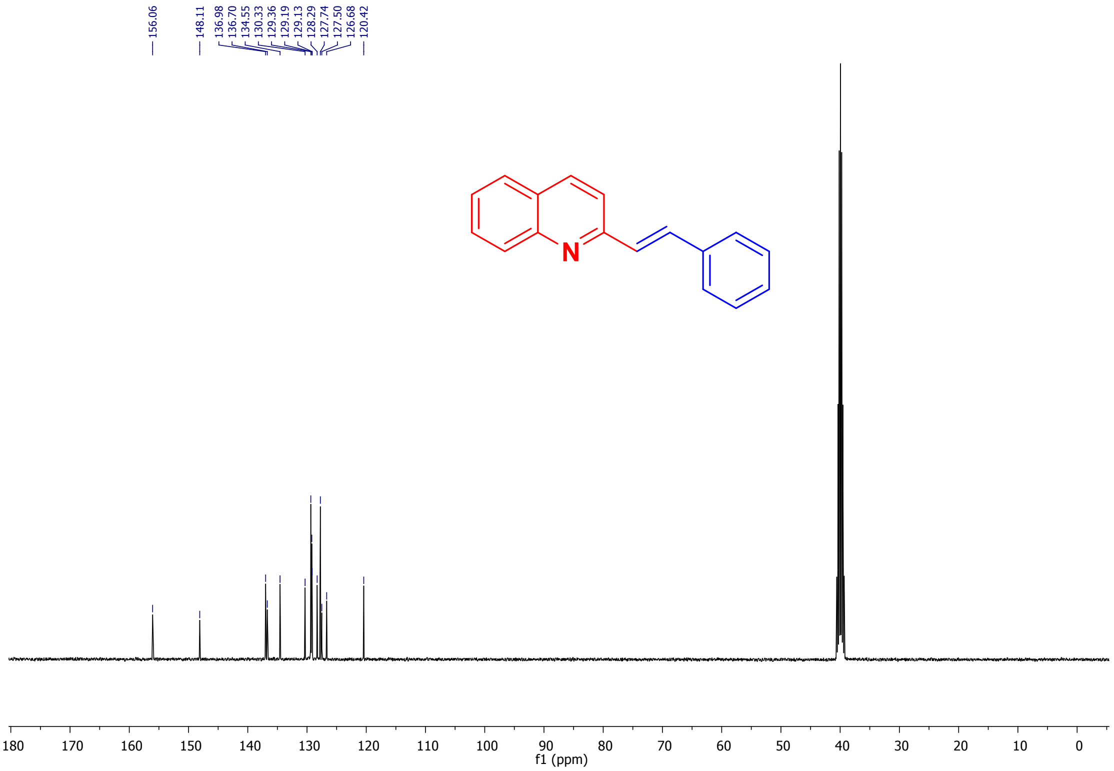

4ai. *(E)-2-(4-methoxystyryl)quinoline*

<u>^{1}H NMR spectrum</u> (400 MHz, DMSO-d_6)

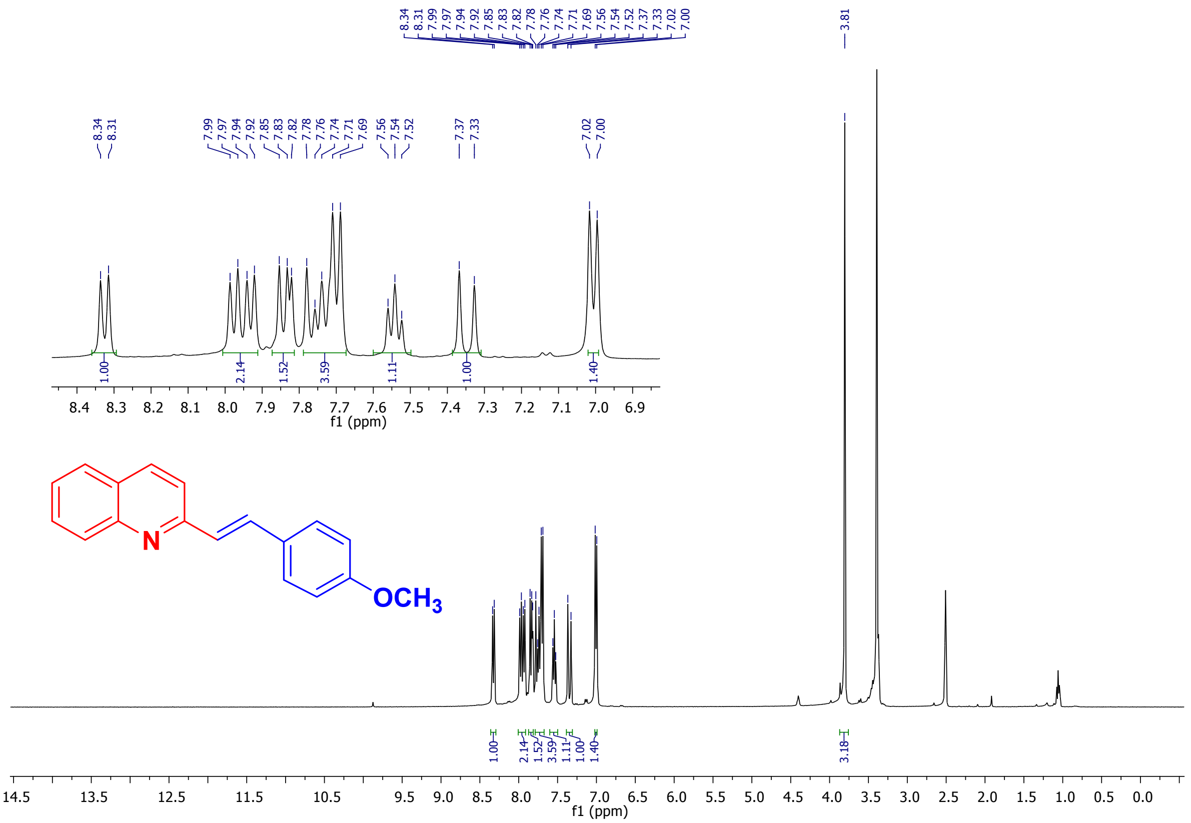

<u>^{13}C NMR spectrum</u> (101 MHz, DMSO-d_6)

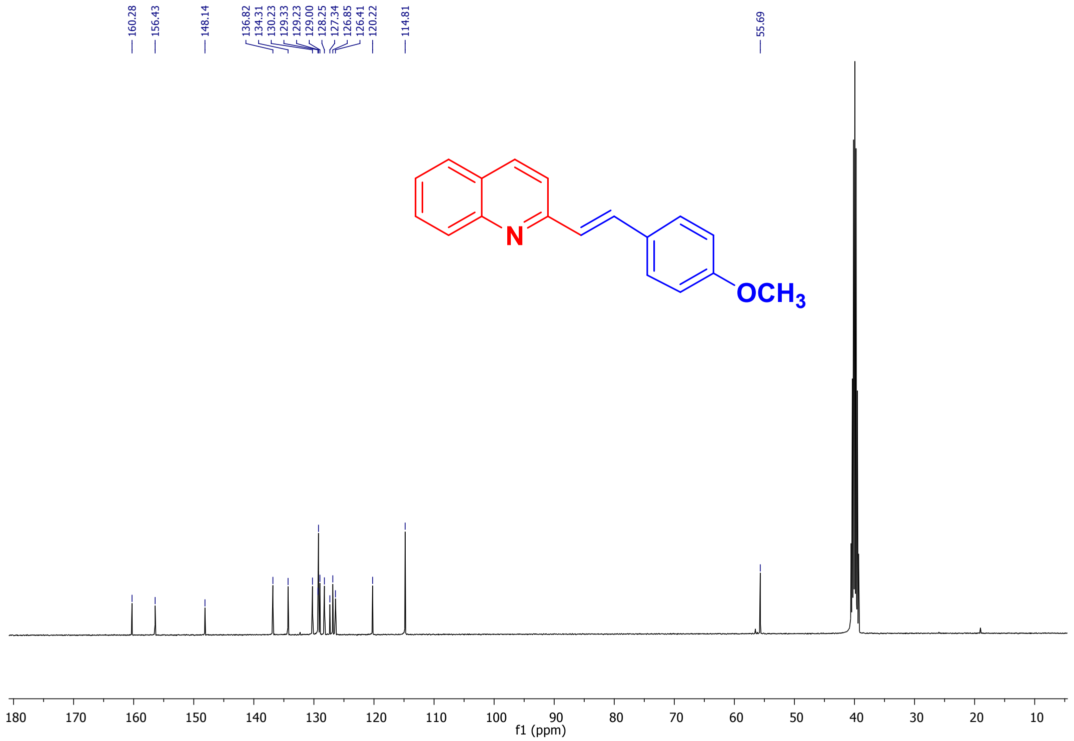

4aj. *(E)-2-(3,4,5-trimethoxystyryl)quinoline*

^{1}H NMR spectrum (400 MHz, DMSO-d_6)

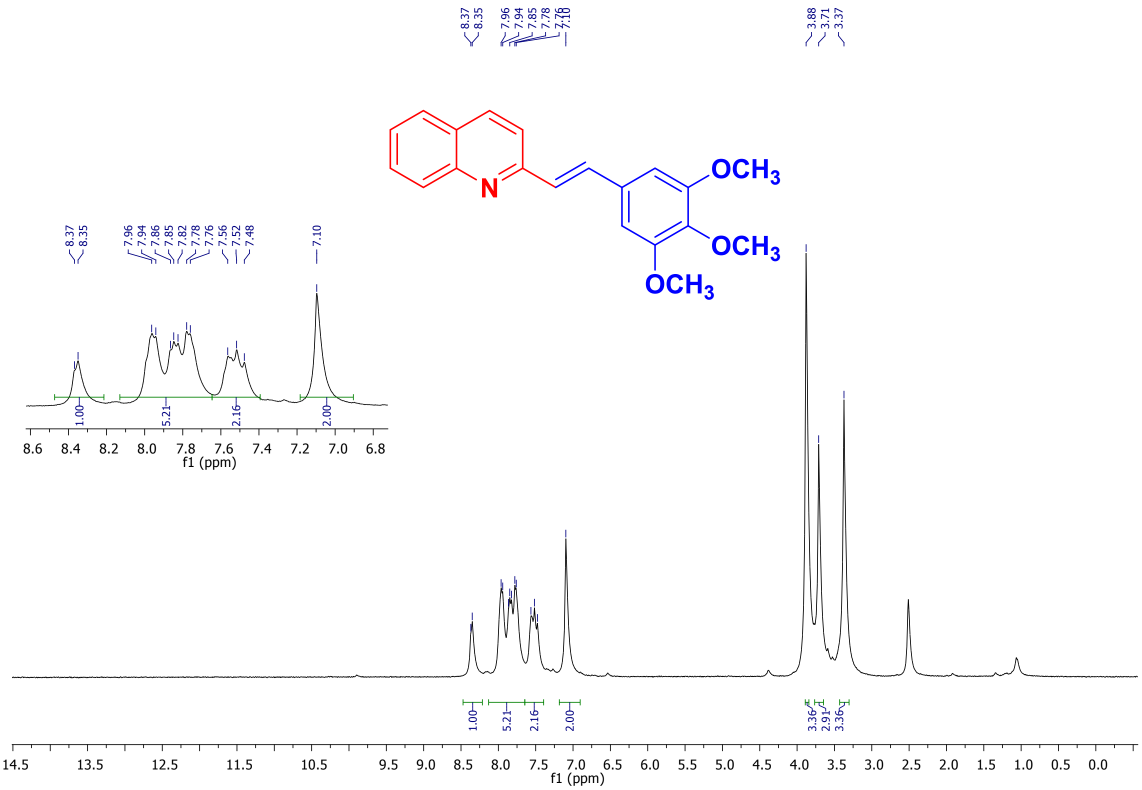

^{13}C NMR spectrum (101 MHz, DMSO-d_6)

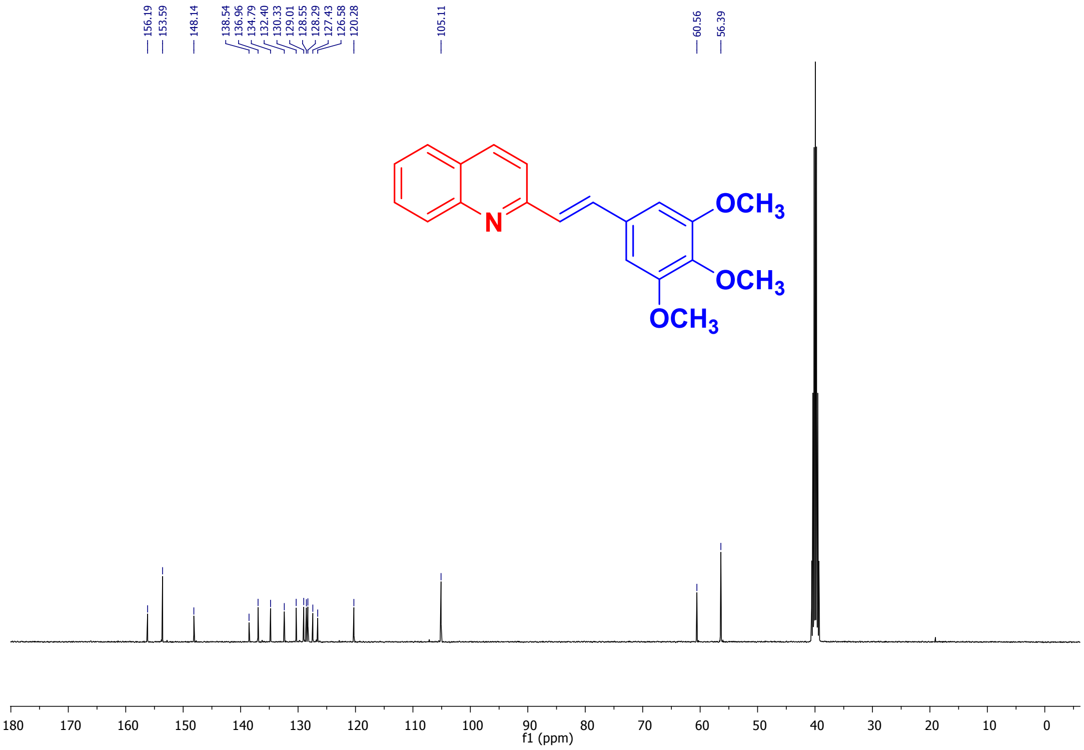

4ak. *(E)-2-(2-chlorostyryl)quinoline*

^{1}H NMR spectrum (400 MHz, DMSO-d_6)

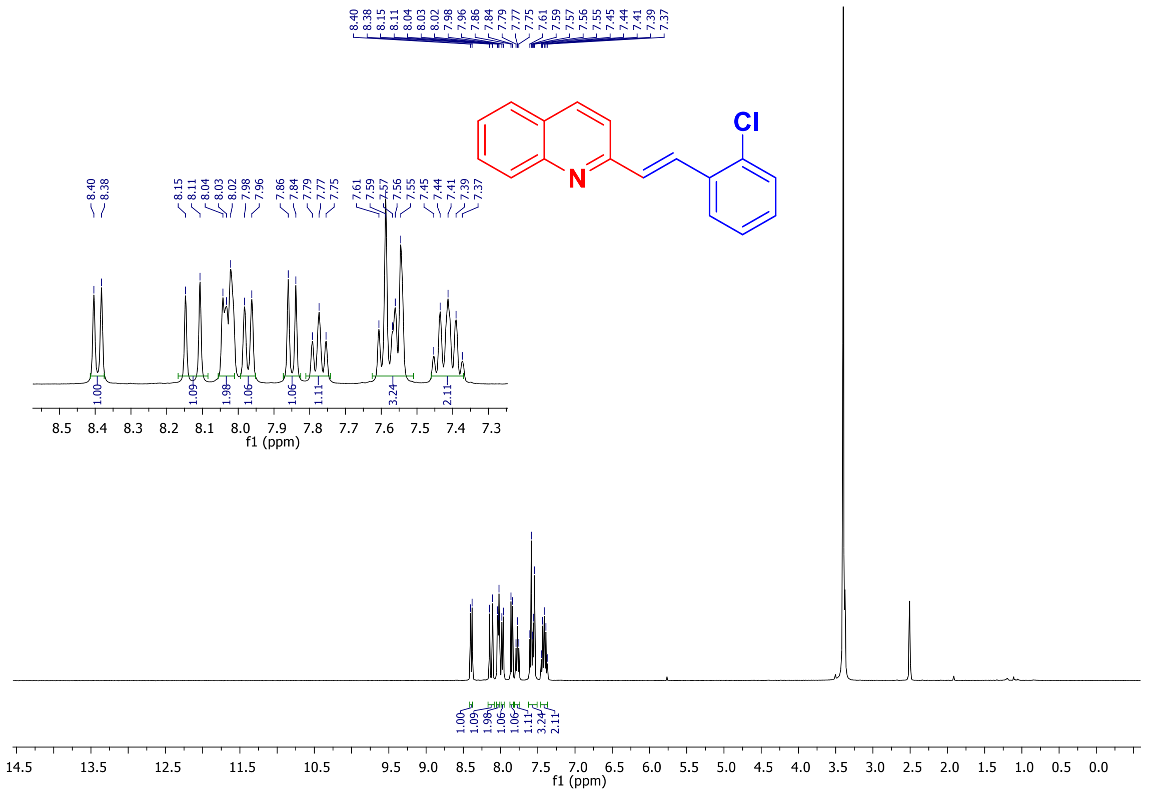

^{13}C NMR spectrum (101 MHz, DMSO-d_6)

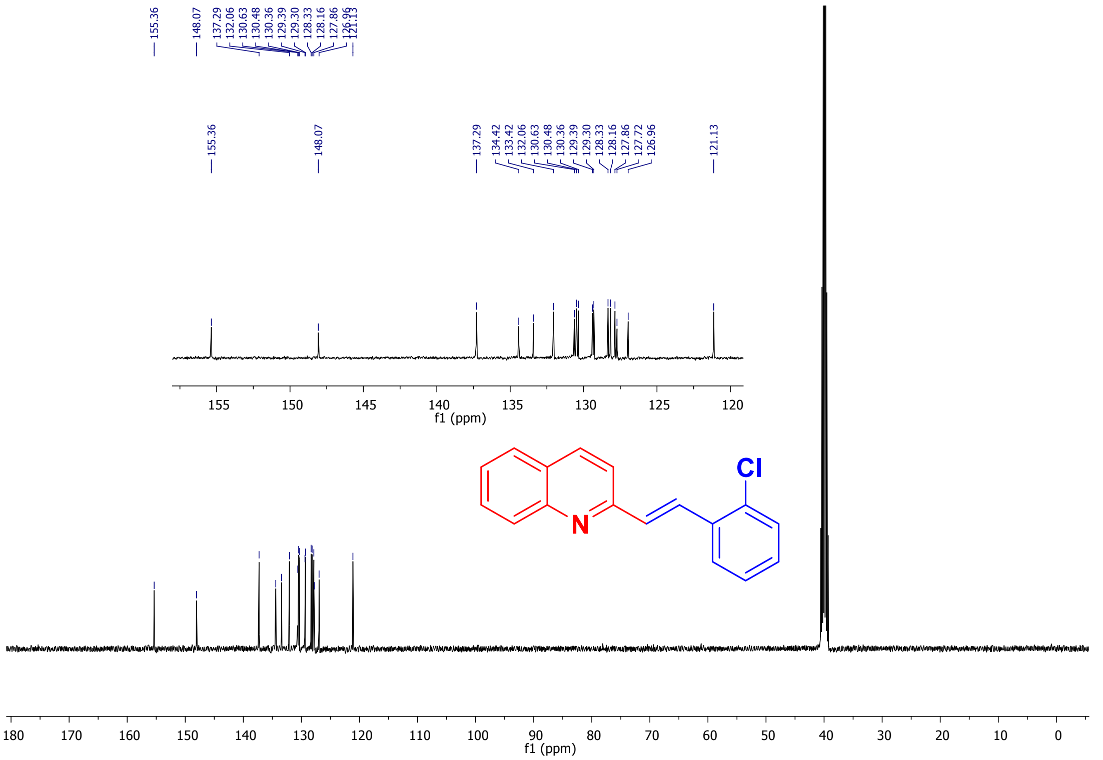

4al. *(E)-2-(2-(thiophen-2-yl)vinyl)quinoline*

^{1}H NMR spectrum (400 MHz, DMSO-d_6)

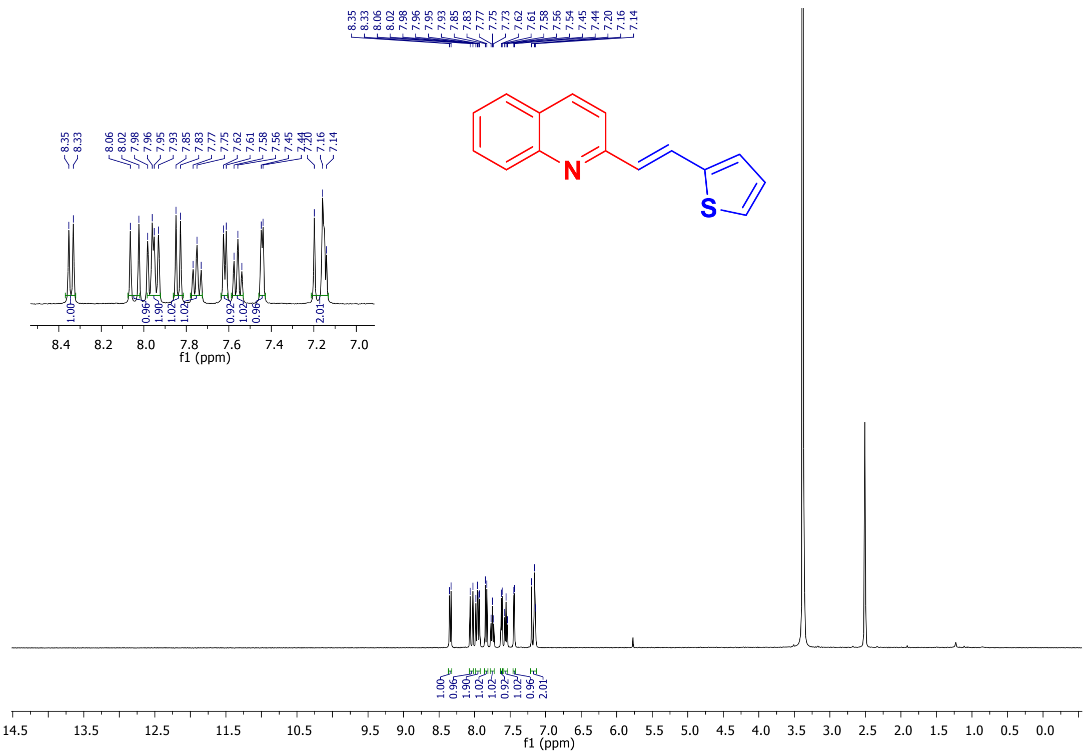

^{13}C NMR spectrum (101 MHz, DMSO-d_6)

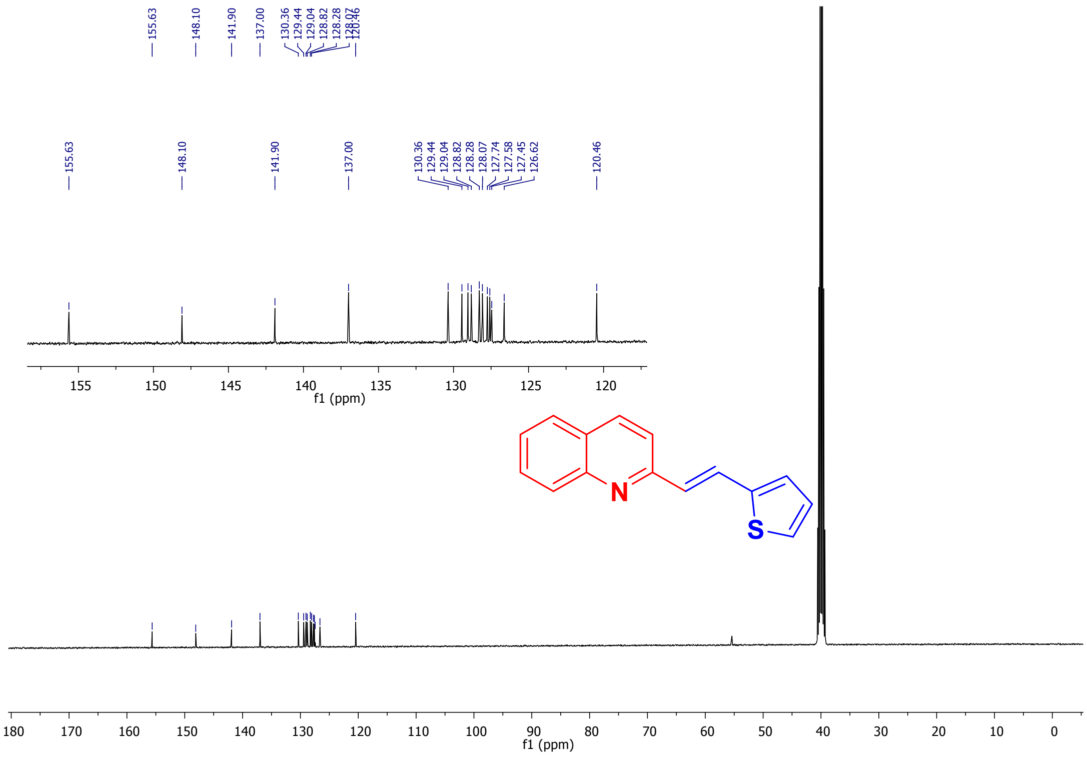

Supplement: RA-010-D0RA02816A-s001 [file RA-010-D0RA02816A-s001.pdf]
